# ACE-Breast-02: a randomized phase III trial of ARX788 versus lapatinib plus capecitabine for HER2-positive advanced breast cancer

**DOI:** 10.1038/s41392-025-02149-3

**Published:** 2025-02-17

**Authors:** Xichun Hu, Qingyuan Zhang, Leiping Wang, Jian Zhang, Quchang Ouyang, Xiaojia Wang, Wei Li, Weimin Xie, Zhongsheng Tong, Shusen Wang, Faliang Xu, Tao Sun, Wei Liu, Zhendong Chen, Jinsheng Wu, Ying Wang, Haixia Wang, Min Yan, Xinshuai Wang, Jingfen Wang, Feilin Cao, Yingying Du, Yongqiang Zhang, Lilin Chen, Ping Lu, Sanyuan Sun, Ruiwen Zhang, Aimin Zang, Xiuqing Nie, Yuan Lei

**Affiliations:** 1 Fudan University Cancer Hospital, Shanghai, 200032 China; 2 Harbin Medical University Cancer Hospital, Harbin, 150081 China; 3 Hunan Cancer Hospital, Changsha, 410013 China; 4 Zhejiang Cancer Hospital, Hangzhou, 310022 China; 5 The First Hospital of Jilin University, Changchun, 130031 China; 6 Guangxi Medical University Affiliated Tumor Hospital, Nanning, 530012 China; 7 Tianjin Medical University Cancer Institute & Hospital, Tianjin, 300060 China; 8 Sun Yat-sen University Cancer Center, Guangzhou, 510060 China; 9 Chongqing University Cancer Hospital, Chongqing, China; 10 Cancer Hospital of China Medical University, Liaoning Cancer Hospital & Institute, Shenyang, 110122 China; 11 The Affiliated Tumor Hospital of Xinjiang Medical University, Urumqi, Xinjiang China; 12 The Second Affiliated Hospital of Anhui Medical University, Hefei, China; 13 The First Affiliated Hospital of Hainan Medical University, Haikou, China; 14 Sun Yat-sen Memorial Hospital, Sun Yat-sen University, Guangzhou, China; 15 Hainan General Hospital, Haikou, China; 16 The Affiliated Cancer Hospital of Zhengzhou University & Henan Cancer Hospital, Zhengzhou, China; 17 The First Affiliated Hospital, College of Clinical Medicine, Medical College of Henan University of Science and Technology, Luoyang, China; 18 Linyi Cancer Hospital, Linyi, China; 19 Taizhou Hospital, Wenzhou Medical University, Taizhou, China; 20 The First Affiliated Hospital of Anhui Medical University, Hefei, China; 21 Beijing Hospital, Beijing, China; 22 First Affiliated Hospital of Xiamen University, Xiamen, China; 23 The First Affiliated Hospital of Xinxiang Medical College, Xinxiang, China; 24 Xuzhou Central Hospital, Xuzhou, China; 25 Sanmenxia Central Hospital, Sanmenxia, China; 26 Affiliated Hospital of Hebei University, Baoding, China; 27 NovoCodex Biopharmaceuticals, Shaoxing, China

**Keywords:** Breast cancer, Breast cancer

## Abstract

This phase III trial aimed to compare ARX788, a site-specific, construct-homogeneous antibody-drug conjugate, with lapatinib plus capecitabine in patients with human epidermal growth factor receptor 2 (HER2)-positive advanced breast cancer (ABC) who had progressed on one line of trastuzumab based regimen. Eligible patients were randomized (1:1) to receive ARX788 (1.5 mg/kg, IV, Q3W) or lapatinib plus capecitabine (LC: lapatinib 1250 mg QD; capecitabine 1000 mg/m^2^ BID, days 1–14, Q3W) and stratified by prior chemotherapy lines (0-1 versus >1) and visceral metastasis (yes versus no). The primary outcome was progression-free survival (PFS) assessed by a blinded independent central review (BICR). A total of 441 patients were randomly assigned to receive either ARX788 (*n* = 221) or LC (*n* = 220). The median PFS was 11.3 (95% confidence interval [CI], 8.4–13.8) months with ARX788 compared with 8.2 (95% CI, 6.9–8.7) months with LC, as per BICR (hazard ratio [HR] 0.64, *p* = 0.0006). Frequencies of treatment-related adverse events (TRAEs) of any grade were 98.6% and 99.1% for ARX788 and LC, respectively. Grade ≥3 TRAEs were 41.4% and 40.0%, respectively, the most common adverse events were blurred vision (12.3%), dry eye (9.1%), keratopathy (5.9%), and interstitial lung disease (ILD, 5.9%) with ARX788; hand-foot syndrome (18.1%) and hypokalemia (5.1%) with LC; all the hematological and gastrointestinal events of grade ≥3 with ARX788 were less than 3%. Six treatment-related deaths occurred, with three cases possibly related to ILD. ARX788 significantly improved PFS compared with LC in patients with HER2-positive ABC with a distinct toxicity profile, supporting it as a potential treatment option.

## Introduction

Breast cancer is one of the most common cancers in the world, and there has been a general increase in incidence rates over recent decades. According to global health statistics, breast cancer is the leading type of cancer and the leading cause of cancer death in women, affecting more than two million new cases each year.^1^ Among the different molecular subtypes of breast cancer, Human Epidermal Growth Factor Receptor 2 (HER2)-positive subtype is characterized by its more aggressive presentation, higher rates of recurrence in early-stage disease, and more rapid progression with shorter disease course in the advanced stage.^2,3^ Approximately 15–20% of all breast cancer cases are HER2-positive in Chinese populations as well as globally.^4,5^ The advent of HER2-targeted drugs, including anti-HER2 monoclonal antibodies such as trastuzumab, small molecule tyrosine kinase inhibitors such as lapatinib, and antibody-drug conjugates (ADCs), has become an important part in the treatment landscape for HER2-positive breast cancer. Compared with HER2-negative breast cancers, patients with HER2-positive breast cancer now have more favorable prognoses in both early-stage and advanced-stage settings, making HER2-positivity no longer a negative prognostic biomarker.^6^

Currently, the global standard of care for second-line treatment of HER2-positive metastatic breast cancer is trastuzumab deruxtecan (T-DXd).^7–9^ In China, however, treatment options have included capecitabine plus tyrosine kinase inhibitors (lapatinib or pyrotinib), or trastuzumab emtansine (T-DM1) since 2022 or T-DXd since 2023.^10,11^ There have been several trials evaluating anti-HER2 therapy in the second-line setting.^12–15^ Pertuzumab did not demonstrate a progression-free survival benefit (PFS) benefit,^12^ whereas tucatinib, lapatinib and pyrotinib had a PFS benefit but all without an overall survival (OS) benefit.^13–15^ T-DXd is the only agent showing benefits of both PFS and OS in initial outcome reporting,^16,17^ quickly establishing it as a new standard of care. However, DESTINY-Breast03 study reported a toxicity-related dose discontinuation rate of 13.6% with T-DXd and utilized strict inclusion criteria regarding history of pulmonary disease, and more than 50% of patients eventually experienced progression. It can be seen that, despite these therapeutic advances enhancing survival rates and quality of life, a majority of patients with HER2-positive advanced breast cancer will eventually experience resistance and progression and a minority of patients with HER2-positive early breast cancer will experience recurrence, highlighting an unmet need for further exploration of newer generations of medications and treatment strategies as an ongoing focus of research.^9,16^ One valuable exploration has been recently published about SYD985, a HER2-targeted ADC containing trastuzumab and vc-seco-DUBA, a cleavable linker, and duocarmycin payload, with a drug-to-antibody ratio (DAR) of 2.4–2.8. The phase III TULIP study evaluated SYD985 against physician’s choice of chemotherapy in patients with unresectable locally advanced or metastatic HER2-positive breast cancer with progression during or after two HER2-targeted therapies or after T-DM1. It met its primary endpoint with modest PFS prolongation (7.0 months versus 4.9 months, HR 0.64, *p* = 0.002), but without an OS benefit in its final analysis, and tolerability was affected by prevalent ocular toxicity, resulting in a discontinuation rate of up to 20.8%, suggesting its unlikely use in the earlier lines of therapy.^18^ In the third- or later-line settings, approved treatments, such as T-DXd, T-DM1, and tucatinib, all have demonstrated OS benefits.^19–21^

Antibody–drug conjugate (ADC) performance can be enhanced through key characteristics, including high construct homogeneity, high stability and wide therapeutic window. Multiple site-specific conjugation techniques are under development, including engineered cysteine, unnatural amino acid (UAA) incorporation, enzymatic ligation, and glycan modification.^22^ ARX788 is a next-generation ADC consisting of an anti-HER2 monoclonal antibody site-specifically conjugated with Amberstatin269 (AS269), a proprietary version of monomethyl auristatin F (MMAF) payload, through a UAA, para-acetyl phenylalanine (pAF). A proprietary EuCODE^TM^ technology platform forms a highly stable oxime bond resulting in a novel ADC with a DAR of approximately two.^23,24^ In preclinical experiments, when compared in vitro against T-DM1 across a panel of cancer cell lines, ARX788 has been shown to be more potent in the HER2-positive and HER2-low cell lines and no activity against normal cardiomyocyte cells. And breast cancer patient-derived xenograft studies confirmed more potent antitumor activities of ARX788 versus T-DM1 in HER2-positive and HER2-low expression tumors, as well as in a T-DM1 resistant model.^25,26^ The clinical benefit of ARX788 in patients with HER2-positive advanced breast cancer (ABC) was reported in a phase I trial conducted in China,^27^ demonstrating promising efficacy and a good safety profile, with an objective response rate (ORR) of 65.5% and a median PFS of 17.02 months at a dose of 1.5 mg/kg every 3 weeks in 29 patients who had progressed on at least two prior anti-HER2 treatments for advanced disease. These results facilitated a head-to-head second-line trial comparing ARX788 with lapatinib plus capecitabine, which constituted a standard regimen in China at the time of trial design.

Here, we present the results of a pre-specified interim analysis from a phase III trial conducted in China, focusing on HER2-positive ABC patients pretreated with trastuzumab and taxane.

## Results

### Patients and baseline characteristics

**Fig. 1:**
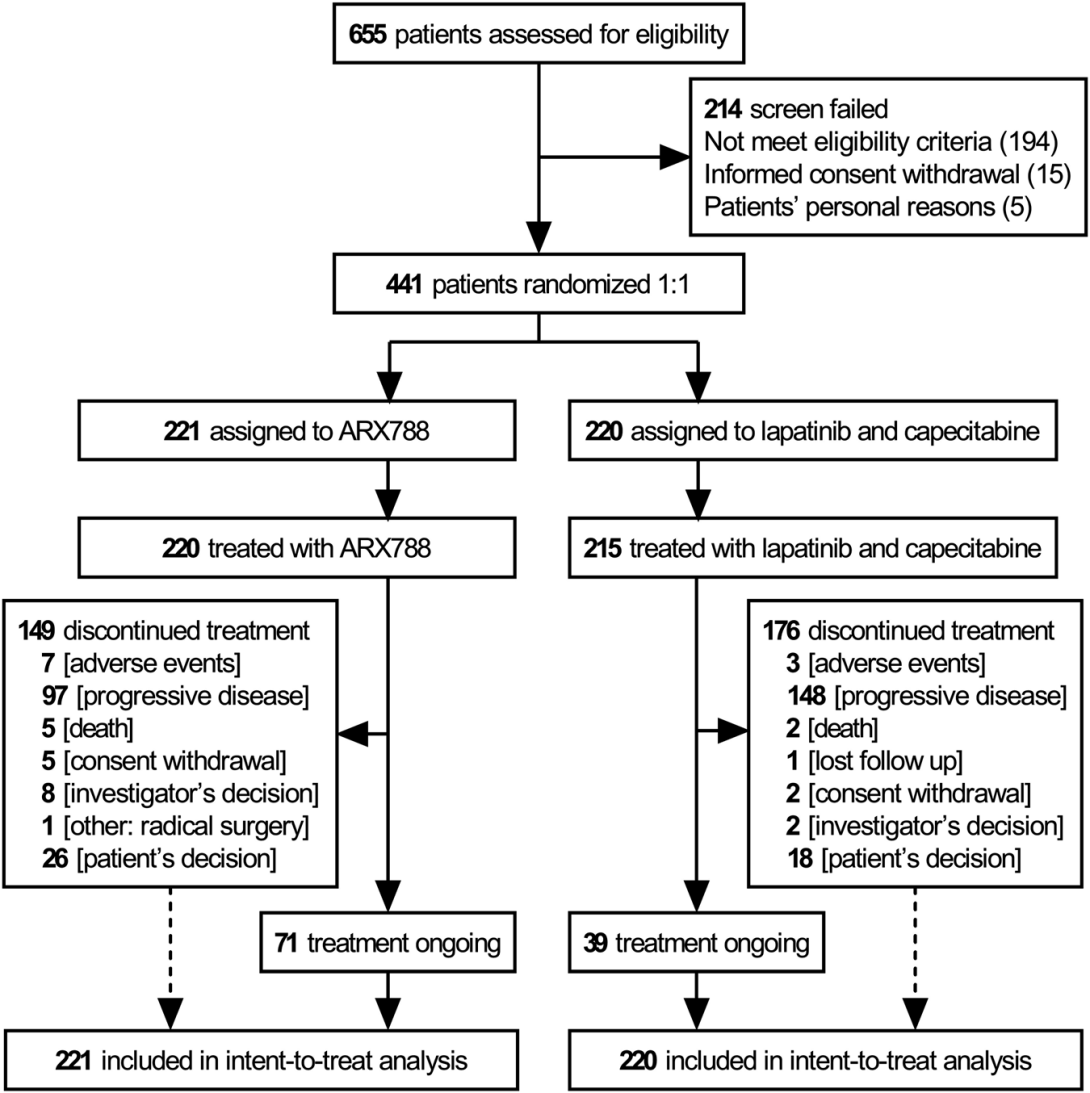
Trial profile

From September 12, 2020, to May 23, 2022, 655 patients from 83 nationwide sites were screened, and 441 eligible patients were randomized. Among these, 221 patients were assigned to the ARX788 group and 220 to the lapatinib plus capecitabine (LC) group. A total of 220 and 215 patients received at least one dose of ARX788 and LC, respectively (Fig. 1).

Baseline characteristics were well balanced between the two groups (Table 1), with a median age of 52 years. Five (2.3%) and eight patients (3.6%) with locally advanced breast cancer not amenable to radical local surgery or radiation were in the ARX788 and LC groups, respectively, all the remaining patients had metastatic disease. Stable brain metastasis was present in 12.7% and 11.8% of patients, while visceral metastasis was observed in 76.0% and 77.7%, respectively. In each group, 18.6% and 19.5% of patients had received more than one line of prior chemotherapy. All patients had previously received trastuzumab and taxane before enrollment, with 34.4% and 28.2% having received pertuzumab in the ARX788 and LC groups, respectively.

**Table 1:** Baseline demographics and characteristics

	ARX788 (*n* = 221)	LC (*n* = 220)
Median age, years	52	52
<60, n (%)	181 (81.9)	176 (80.0)
≥60, n (%)	40 (18.1)	44 (20.0)
ECOG performance status, n (%)		
0	103 (46.6)	98 (44.5)
1	118 (53.4)	122 (55.5)
Median time from first diagnosis of metastasis to randomization, months	15.0	13.7
Stable brain metastasis, n (%)	28 (12.7)	26 (11.8)
Visceral metastasis, n (%)	168 (76.0)	171 (77.7)
HER2 status*, n (%)		
IHC 1+ or IHC 2 + /FISH+	86 (38.9)	80 (36.4)
IHC 3+	135 (61.1)	140 (63.6)
Hormone receptor status, n (%)		
Positive	117 (52.9)	108 (49.1)
Negative	102 (46.2)	109 (49.5)
Missing	2 (0.9)	3 (1.4)
No. of prior chemotherapy lines in the metastatic setting		
No. of lines (range)	1 (1, 4)	1 (1, 4)
No. of lines, n (%)		
0-1	180 (81.4)	177 (80.5)
>1	41 (18.6)	43 (19.5)
No. of prior treatment lines in the metastatic setting, n (%)		
0	0 (0.0)	1 (0.5)
1	147 (66.5)	146 (66.4)
≥2	74 (33.5)	73 (33.2)
Previous anti-tumor treatment, n (%)		
Trastuzumab	221 (100.0)	220 (100.0)
Pertuzumab	76 (34.4)	62 (28.2)
Taxane	221 (100.0)	220 (100.0)
Anthracycline	142 (64.3)	147 (66.8)
Capecitabine	22 (10.0)	25 (11.4)
Anti-HER2 TKI	7 (3.2)	6 (2.7)
Other target therapy	6 (2.7)	5 (2.3)
Hormone therapy	89 (40.3)	76 (34.5)

^*^, HER2 status results were all from central laboratory

*LC* lapatinib plus capecitabine, *ECOG* Eastern Cooperative Oncology Group, *HER2* human epidermal growth factor receptor 2, *IHC* immunohistochemistry, *FISH* fluorescence in situ hybridization, *TKI* tyrosine kinase inhibitor

### Efficacy

**Fig. 2:**
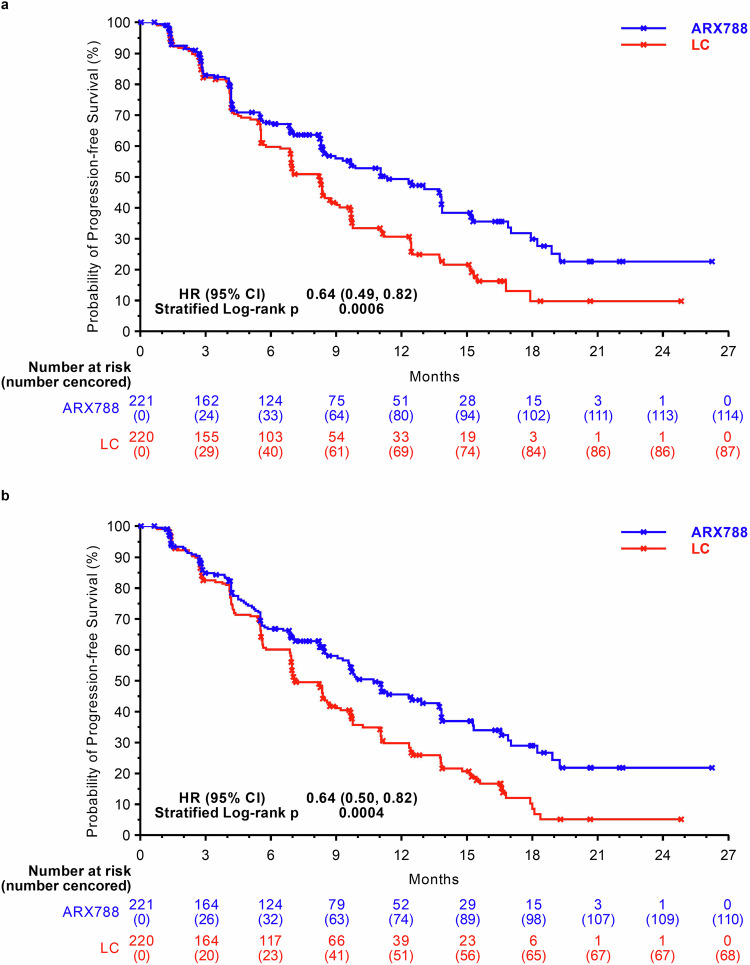
Kaplan–Meier curves for PFS. **a** PFS assessed by BICR. **b** PFS assessed by INV. PFS progression-free survival, BICR blinded independent central review, INV investigators, LC lapatinib plus capecitabine

**Fig. 3:**
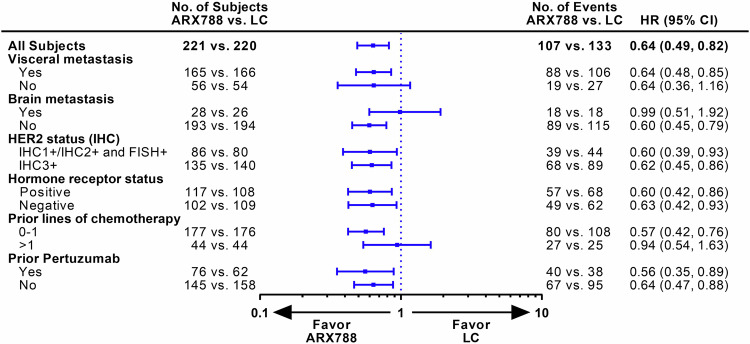
PFS assessed by BICR in key subgroups. PFS progression-free survival, LC lapatinib plus capecitabine

The data reported here are from the second interim analysis (data cutoff: December 21, 2022), conducted after 240 patients experienced disease progression or death, with a median follow-up of 11.1 months and a two-sided alpha level of 0.0162 (stopping boundary). The blinded independent central review (BICR) assessment identified 107 (48.4%) and 133 (60.5%) PFS events in the ARX788 and LC groups, respectively. The median PFS was significantly longer in the ARX788 group, at 11.3 (95% confidence interval [CI], 8.4–13.8) months, compared with 8.2 (95% CI, 6.9–8.7) months in the LC group (HR 0.64, 95% CI: 0.49–0.82, *p* = 0.0006 by stratified log-rank test) (Fig. 2a). One-year PFS rates were 49.3% (95% CI, 41.3–56.8%) and 30.7% (95% CI, 23.3–38.3%), respectively, with an absolute difference of 18.6%. The significance was also found in the PFS assessed by investigators, with a median PFS of 10.7 (95% CI, 9.1–13.7) months and 7.1 (95% CI, 6.9–8.6) months (HR 0.64, 95% CI: 0.50–0.82, *p* = 0.0004 by stratified log-rank test), respectively (Fig. 2b). PFS benefits with ARX788 were broadly observed in all key subgroups (Fig. 3), showing a positive treatment effect generally consistent with the overall treatment effect.

**Fig. 4:**
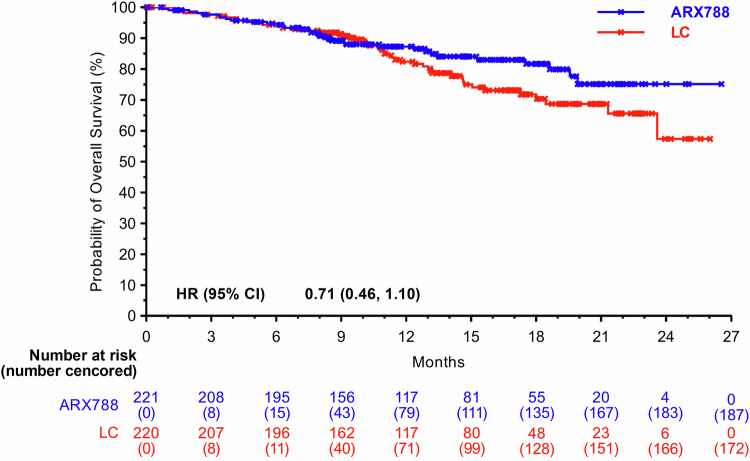
Kaplan–Meier curves for OS. OS overall survival, LC lapatinib plus capecitabine

**Fig. 5:**
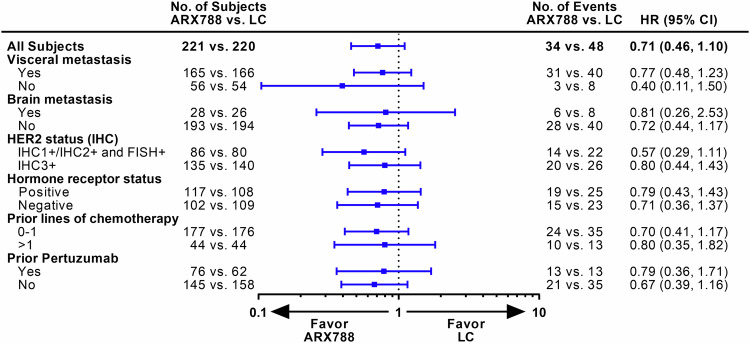
OS in key subgroups. OS overall survival, LC lapatinib plus capecitabine

The reported number of deaths was 34 (15.4%) out of 221 in the ARX788 group and 48 (21.8%) out of 220 patients in the LC group, respectively. The median OS was not reached, one-year OS rate were 87.3% (95% CI, 81.8–91.3%) and 82.4% (95% CI, 75.9–87.3%), respectively. A favorable trend in OS was indicated and consistent results were observed across all key subgroups (Figs. 4, 5).

The ORR was achieved in 144 of 221 patients (63.8%, 95% CI: 57.1–70.1%) in the ARX788 group and 116 of 220 patients (52.7%, 95% CI: 45.9–59.5%) in the LC group, with a difference of 11.1% (95% CI, 1.9–20.2%, *p* = 0.0186). Complete response (CR) was observed in 5.4% of patients in the ARX788 group and 3.6% in the LC group. The median duration of response (DoR) was significantly longer in the ARX788 group at 12.5 (95% CI, 10.9–15.1) months compared with 8.3 (95% CI, 6.9–11.1) months in the LC group (*p* = 0.0017).

### Subsequent anti-tumor therapy

At the data cutoff date, 67.4% of patients in the ARX788 group and 80.0% of patients in the LC group had discontinued study treatment. In the subsequent anti-tumor therapy (Table 2), there were higher rates of anti-HER2 monoclonal antibodies (27.3% versus 14.0%) and ADCs in the LC group (20.0% versus 1.4%), while tyrosine kinase inhibitors (TKIs) use was more common in the ARX788 group (38.0% versus 26.8%).

**Table 2:** Subsequent anti-tumor therapy after study treatment discontinuation

	ARX788 (*n* = 221)*	LC (*n* = 220)*
Any	104 (47.1)	140 (63.6)
Radiotherapy	10 (4.5)	16 (7.3)
Operation	1 (0.5)	2 (0.9)
Drug therapy	99 (44.8)	131 (59.5)
Chemotherapy	89 (40.3)	95 (43.2)
Targeted agents	93 (42.1)	122 (55.5)
Anti-HER2 monoclonal antibody	31 (14.0)	60 (27.3)
Anti-HER2 ADC	3 (1.4)	44 (20.0)
Anti-HER2 TKI	84 (38.0)	59 (26.8)
Others	9 (4.1)	10 (4.5)
Anti-CDK4/6 agents	4 (1.8)	3 (1.4)
Anti-VEGF agents	2 (0.9)	4 (1.8)
Anti-mTOR agents	1 (0.5)	0 (0.0)
Anti-PARP agents	0 (0.0)	1 (0.5)
Anti-HER2/CD3 agents	1 (0.5)	1 (0.5)
Anti FR/TRPV6 agents	1 (0.5)	1 (0.5)
Others	1 (0.5)	0 (0.0)
Immunotherapy	0 (0.0)	4 (1.8)
Hormone therapy	9 (4.1)	6 (2.7)
Other systemic therapy	4 (1.8)	7 (3.2)

Data are the n (%)

* ARX788 and LC groups had 71 (32.1%) and 39 (17.7%) patients receiving study assigned treatments at the data cutoff, respectively

*LC* lapatinib plus capecitabine, *HER2* human epidermal growth factor receptor 2, *ADC* antibody-drug conjugate, *TKI* tyrosine kinase inhibitor, *CDK4/6* cyclin-dependent kinase 4/6, *VEGF* vascular endothelial growth factor, *mTOR* mammalian target of rapamycin, *PARP* poly ADP-ribose polymerase, *FR* folate receptor, *TRPV6* Transient Receptor Potential Channel Subfamily V Member 6

### Safety

The median number of treatment cycles was 8.0 (range, 1–30) in the ARX788 group and 9.0 (range, 1–32) in the LC group. Treatment-related adverse events were reported in 217 (98.6%) of 220 patients in the ARX788 group and 213 (99.1%) of 215 patients in the LC group. Grade ≥3 treatment-related adverse events (TRAEs) occurred in 91 (41.4%) of 220 and 86 (40.0%) of 215 patients with ARX788 and LC, respectively. Dose reduction due to TRAEs occurred in 23.6% and 37.7% patients, respectively. Discontinuation due to TRAEs occurred in 6.8% of patients in the ARX788 group, primarily due to interstitial lung disease (ILD, 3.2%) and lung infection (2.7%). In the LC group, four patients discontinued treatment due to TRAEs, including two cases of gastrointestinal disease and one case of arrhythmia.

The most frequently reported TRAEs at any grade (>20%) and grade ≥3 in the ARX788 and LC groups are shown in Table 3. In the ARX788 group, TRAEs mainly included hepatic enzyme increased, dry eye, blurred vision, alopecia, and ILD. The most frequent grade ≥3 TRAEs in the ARX788 group were blurred vision (12.3%), dry eye (9.1%), keratopathy (5.9%), and ILD (5.9%). In the LC group, predominant grade ≥3 TRAEs were hand-foot syndrome (18.1%) and hypokalemia (5.1%). The rate of grade ≥3 increase of hepatic enzymes was less than 3%. The rate of grade ≥3 hematological toxicity was also less than 3%, and that of grade ≥3 gastrointestinal toxicity was less than 1%.

**Table 3:** Most common treatment-related adverse events in ARX788 and LC groups (≥20%) and hematological toxicity and GI toxicity

	ARX788 (*n* = 220)		LC (*n* = 215)	
	Any	≥Grade 3	Any	≥Grade 3
Hepatotoxicity				
Aspartate aminotransferase increased	156 (70.9%)	3 (1.4%)	113 (52.6%)	1 (0.5%)
Alanine aminotransferase increased	131 (59.5%)	5 (2.3%)	74 (34.4%)	4 (1.9%)
Blood bilirubin increased	14 (6.4%)	0 (0.0%)	114 (53.0%)	6 (2.8%)
Ocular toxicity				
Dry eye	121 (55.0%)	20 (9.1%)	2 (0.9%)	0 (0.0%)
Blurred vision	77 (35.0%)	27 (12.3%)	1 (0.5%)	0 (0.0%)
Keratopathy	62 (28.2%)	13 (5.9%)	0 (0.0%)	0 (0.0%)
Conjunctivitis	55 (25.0%)	5 (2.3%)	4 (1.9%)	0 (0.0%)
Skin and subcutaneous tissue disorders				
Alopecia	89 (40.5%)		3 (1.4%)	
Hand foot syndrome	0 (0.0%)	0 (0.0%)	128 (59.5%)	39 (18.1%)
Respiratory toxicity				
ILD	72 (32.7%)	13 (5.9%)	1 (0.5%)	0 (0.0%)
Pneumonitis	44 (20.0%)	2 (0.9%)	3 (1.4%)	1 (0.5%)
Metabolism disorders				
Hypokalemia	61 (27.7%)	10 (4.5%)	47 (21.9%)	11 (5.1%)
Weight loss	28 (12.7%)	3 (1.4%)	52 (24.2%)	0 (0.0%)
Hematological toxicity				
Platelet count decreased	65 (29.5%)	6 (2.7%)	13 (6.0%)	1 (0.5%)
White blood cell decreased	39 (17.7%)	1 (0.5%)	65 (30.2%)	1 (0.5%)
Neutrophil count decreased	34 (15.5%)	5 (2.3%)	59 (27.4%)	7 (3.3%)
Anemia	25 (11.4%)	2 (0.9%)	66 (30.7%)	4 (1.9%)
GI toxicity				
Nausea	29 (13.2%)	1 (0.5%)	38 (17.7%)	1 (0.5%)
Vomiting	20 (9.1%)	0 (0.0%)	33 (15.3%)	1 (0.5%)
Diarrhea	11 (5.0%)	1 (0.5%)	93 (43.3%)	9 (4.2%)

Data are the n (%)

*LC* lapatinib plus capecitabine, *ILD* interstitial lung disease, *GI* gastrointestinal

Ocular toxicity related to ARX788 occurred in 74.5% of patients, manifested mainly as dry eye (55%), blurred vision (35%), and keratopathy (28.2%) (Table 3). None of the patients developed perforation, ulceration, or blindness. No grade 4 events occurred. None of the patients discontinued treatment due to ocular toxicity, and ocular toxicity were mild to moderate and reversible following discontinuation of the treatment. The median time from the first dose to the onset was 29.5 days. Among the subset of patients with grade 3 toxicity (accounting for 19.1% of all patients), approximately 55% of them had self-care activity of daily life compromised, while the remaining 45% did not, and only experienced >3 lines of decreased vision from the baseline.

ILD was reported in 72 patients (32.7%) and 1 patient (0.5%) in the ARX788 and LC groups, respectively. Frequencies of grade 1 and 2 were 11.4% and 15.5%, respectively, accounting for the majority of ILD events. Grade ≥3 ILD occurred in 13 patients (5.9%), with three deaths (1.4%) judged to be possibly related to ILD. The median onset time of ILD was 129 days. When combined with pneumonitis, grade ≥3 ILD/pneumonitis occurred in 15 patients (6.8%), with four deaths (1.8%) judged to be related to ILD or pneumonitis. Thirty patients (13.6%) had pulmonary diseases at baseline, mainly including 17 cases of chronic pneumonitis, followed by cough or expectoration, pleural effusion, pulmonary emphysema, bronchiectasis, etc. These patients developed a statistically higher risk of pulmonary toxicity compared to the other 190 patients who did not, at any grade and grade ≥3 (*p* = 0.0365 and 0.0253, respectively).

Overall, in the ARX788 group, six (2.7%) were reported to have grade 5 TRAE, including three cases of ILD, two cases of respiratory failure (one with lung infection, the other with COVID-19 infection), and one case of pneumonitis. Among the six patients, three cases had chronic pneumonitis, and four had lung metastases at baseline. No treatment-related deaths were reported in the LC group.

## Discussion

ARX788 is one of the first site-specific construct-homogeneous ADCs and the first to be assessed in a phase III trial. This pivotal trial demonstrates a significant improvement in PFS with ARX788 monotherapy compared with lapatinib plus capecitabine in patients with HER2-positive ABC who had previously been treated with trastuzumab and taxane and had progressed on one line of trastuzumab-based treatment. The observed median PFS of 11.3 months with ARX788 versus 8.2 months with the control regimen (HR 0.64, *p* = 0.0006) met the statistical hypothesis in the pre-specified interim analysis. This PFS benefit was further confirmed by investigator assessment. Both response and its duration supported the clinical benefit of ARX788. Moreover, there appeared to be a trend of overall survival benefit towards favoring ARX788.

Before the advent of T-DXd, the median PFS was approximately 6–9 months for patients who were resistant to trastuzumab in HER2-positive ABC,^14,28^ whereas ARX788 achieved a median PFS of 11.3 months in this randomized trial, warranting further exploration of its clinical value. Moreover, broadly consistent benefits were demonstrated across all key subgroups. In terms of stratified factors, including visceral metastasis and prior chemotherapy lines, median HRs were less than one for both the non-visceral metastasis and prior more than one chemotherapy line subgroups, but the upper limits of the 95% CI crossed 1, which might be related to the relatively small sample size. Of note, a median PFS of 8.2 months in the control arm was slightly longer than reported in other randomized trials on lapatinib plus capecitabine.^15,25,28^ Interestingly, lapatinib-based regimens appear to have higher anti-tumor activities in Chinese breast cancers with ORR ranging from 52% to 57.1% in the second-line setting and 69% in the first-line setting (predominantly Chinese patients) in randomized trials.^15,29,30^ Despite this, ARX788 still demonstrated significant benefit over the LC regimen.

ARX788 was generally tolerated and the toxicity-related drug discontinuation rate was only 6.8%. The two treatment groups had similar rates of TRAEs in terms of both any grade and grade ≥3 toxicity in this trial. From a phase I study (NCT03255070) conducted in the USA, results of 42 heavily pretreated breast cancer patients were released at the San Antonio Breast Cancer Symposium (SABCS) 2023, ARX788 was reported to be generally well tolerated, with grade 3 TRAEs occurring in 23.8% of patients. Only one (2.4%) patient had grade 3 ILD, and there were no grade 4 or 5 events.^31^

Grade ≥3 toxicities of ARX788 were primarily ocular and pulmonary toxicities. Ocular toxicity was an early-onset event with a median time of about a month after the first dose of ARX788, and mainly manifested as dry eye and blurred vision. There would be almost no new cases nor any case of deterioration after three months following drug administration. No primary prophylactic measures were implemented until patients had experienced any grade of ocular toxicity. Fortunately, several measures, including the frequent use of ocular lubricants such as sodium hyaluronate, eyedrops containing calf serum, and measures indicated by ophthalmologists, have been shown to mitigate ocular toxicity and are routinely used in the administration of mirvetuximab soravtansine and belantamab mafodotin.^18,32–35^ In future clinical trials with ARX788, to improve patient quality of life, it is recommended to implement primary prophylactic measures and ensure early involvement by ophthalmologists although there was no dose discontinuation due to this toxicity in this trial.

Another common toxicity was ILD, with a rate of 32.7% at all grades and 5.9% at grade 3 or higher, which was numerically higher than 10.5% and 0.8% reported in the first interim analysis of the DESTINY-Breast03 trial with T-DXd, respectively.^16^ The causes of the higher rates reported in this study may include enrollment of patients with baseline chronic pulmonary disease, pulmonary toxicity being an adverse events of special interest, chest CT scans done every six weeks during the trial period as per protocol, greater awareness of anti-tumor drug-related pulmonary toxicity, and increased risk with targeted biologicals in Eastern Asian patients.^16,36,37^ In some trial protocols in the same clinical setting, patients who did not have metastatic lesions in the chest were not required to have chest CT scans at every-six-week efficacy assessments.^38^ In addition, under more frequent chest CT scans and more stringent diagnostic criteria, 52.3% of everolimus-related pneumonitis were reported, with predominantly low grades.^39^ Moreover, different approaches in the management of grade 1 or 2 ILD may also contribute to the reporting of higher-grade events, since in this trial our patients could continue ARX788 at reduced doses. Among the 13 patients with grade ≥3 ILD, the early use of high-dose steroids, with at least 360 mg of methylprednisolone per day as the starting dose in four patients, successfully reversed ILD with no deaths. While three deaths in the other nine patients, treated with lower doses or delayed use of steroids, were judged to be possibly related to ILD. The failure to implement protocol-suggested steroid doses in patients in this trial was partially attributable to the current ILD guidelines in China, which cover all etiologies.^40,41^ Additional interventions for ILD used in this trial encompassed educations aimed at investigators, patients, and their families; vigilant monitoring, such as frequent assessments of oxygen saturation levels below 95% utilizing an fingertip pulse oximeter, regardless of location and time; and proactive management strategies, including the administration of anti-infection medications when clinical indicators suggested a potential infection, in conjunction with steroid therapy. Of note, ARX788-related ILD was a late-onset event, with a median time of approximately four months after the first dose of ARX788, requiring hypervigilance, proactive monitoring, and prompt, adequate steroid use throughout the entire treatment course.

One notable feature regarding the safety of ARX788 was low rates of hematological and gastrointestinal toxicity under no pre-medication, which compared favorably with other ADCs approved in this setting, although direct comparisons of different clinical trials may introduce bias.^17,29^ One possible explanation for the low toxicity is that the payload itself is less emetogenic and less myelosuppressive. Another contributing factor might be the low free payload blood concentration. Zhang et al. showed that the ratio of free payload to the total antibody of ARX788 in the peak concentration in the circulation was 0.1%, which was possibly the lowest among the three ADCs.^27^ Its high stability in the blood, probably resulting from site-specific, construct-homogeneous conjugation technology, might also contribute to its low hematological and gastrointestinal toxicities.

A limitation of this study is the difficulty in interpreting it in the context of standard second-line T-DXd treatment. A phase I study (NCT03255070) of ARX788 monotherapy, conducted by Frentzas et al. in 42 heavily pretreated breast cancer patients in the United States,^31^ reported that the median number of prior lines was six, and the ORR was 54.5% in HER2-positive patients and 23.3% in HER2-low patients. Of eight patients with prior T-DXd exposure, six also had prior T-DM1, and its anti-tumor activity was primarily observed. Based on these promising results,^31^ a phase II study (NCT 04829604) is ongoing to evaluate the efficacy and safety of ARX788 in HER2-positive breast cancer patients with heavy pre-treatment, including T-DXd. This study, reported at SABCS 2022 for seven patients pretreated with T-DM1, showed a confirmed ORR of 57.1%, an unconfirmed ORR of 71.4%, and a disease control rate (DCR) of 100%,^42^ which may provide some valuable insight. Of particular note, its low toxicity in hematological and gastrointestinal systems may provide an alternative option.

In conclusion, ARX788, a site-specific construct-homogeneous ADC, demonstrated a significant PFS benefit in the second-line treatment of HER2-positive ABC patients who had progressed on one line of trastuzumab based regimen and had been pretreated with taxane. The two common toxicities, pulmonary and ocular, were generally manageable. Given its potency and distinct safety profile, ARX788 has the potential to be an alternative second-line treatment option for patients. Trials focusing on HER2-low and brain metastatic patients in China (CTR20222247, NCT05018702), as well as post-T-DXd and neoadjuvant use in the I-SPY II platform in the USA (NCT 04829604, NCT 01042379) are all actively ongoing.

## Materials and methods

### Study design and participants

ACE-Breast-02 is a multicenter, open-label, randomized, controlled phase III trial conducted in 83 centers in China. Eligible patients were aged 18-75 years, with recurrent or metastatic breast cancer or locally advanced breast cancer not amenable to radical local surgery or radiation, had centrally confirmed HER2-positive (HER2 IHC 3+ or IHC 1 + /2+ but FISH + ) breast cancers, had progressed on at least one line of trastuzumab based regimen for recurrent or metastatic disease or had relapsed or progressed during or within 12 months after neoadjuvant or adjuvant trastuzumab lasting ≥9 weeks, had been pretreated with taxane, had an Eastern Cooperative Oncology Group (ECOG) performance status of 0 or 1, had at least 1 measurable lesion according to the Response Evaluation Criteria in Solid Tumors (RECIST) version 1.1, and had adequate organ function. Patients who had previously used T-DM1 or other HER2-targeted ADCs were excluded from this trial. Written informed consent was obtained from all participants before study enrollment. The protocol and all amendments were approved by a local institutional review board or independent ethics committee at each study site.

### Randomization and masking

Patients were randomly assigned (1:1) to ARX788 or LC using a stratified permuted block method of size 4, with chemotherapy lines (0-1 versus >1) and visceral metastasis (yes versus no) as the stratification factors. The randomization statistician, independent of the sponsor and the study team (including study statistician) generated the random number tables using SAS 9.4. Eligible patients were allocated to the treatment group directly via a central interactive web response system (IWRS). Due to different administration routes of ARX788 and LC, blinding to patients or investigators was not feasible. A BICR was established to centrally perform imaging assessments. All the central reviewers were masked to the treatment assignments.

### Procedures

Patients received intravenous ARX788 1.5 mg/kg every three weeks or lapatinib plus capecitabine (LC: lapatinib 1250 mg, QD; capecitabine 1000 mg/m2 twice daily, days 1–14, every three weeks). Treatment was continued until disease progression, intolerable toxicity, or withdrawal of informed consent, whichever occurred first. ARX788 was not pre-medicated with any prophylactic measures until any degree of toxicity was evident, such as sodium hyaluronate and calf serum for any grade of ocular toxicity. Adverse events were classified and graded using the National Cancer Institute Common Terminology Criteria for Adverse Events (NCI CTCAE) Version 5.0. Protocol-defined dose reduction of ARX788 during treatment to manage toxicity was permitted, with reduction at most to three dose levels including 1.3 mg/kg, 1.1 mg/kg, and 0.88 mg/kg. Only patients who had experienced grade ≥3 ILD had to discontinue treatment, while patients with grade 1 or 2 ILD could continue treatment with a dose delay or a dose reduction to one lower dose level.

All participants were required to complete the relevant visits and clinical examinations or observations within the specified time in the protocol. The frequency of patient visits was every cycle until the last treatment visit within 28 to 35 days after the last treatment. Blood tests (blood biochemistry and blood routine) were monitored before each dose and at the last treatment visit, and as well as on cycle 1, day 7 and day 14. Tumor imaging, which included chest, abdomen, and pelvis CT/MRI, and brain MRI at baseline and later when indicated, was performed at baseline and every six weeks thereafter until disease progression, death, visit refusal or the end of the study, whichever occurred first. All lesions were assessed according to RECIST version 1.1. After disease progression or visit refusal, survival follow-up was conducted via telephone every three months.

### Outcomes

The primary outcome was PFS assessed by BICR. Secondary outcome measures included OS, PFS assessed by investigators, ORR, DCR, DoR, and safety.

### Statistical analysis

For the primary endpoint of PFS, a total of 335 events were needed to detect an HR of 0.70 using a log-rank test at the two-sided 0.05 level with 90% power. Considering the median PFS for the control group was 6.4 months, assuming an accrual period of 24 months and an annual drop-out rate of 5%, 440 patients (220 in each group) were needed to achieve the targeted number of events approximately 8 months after the last participant was randomized.

Two interim analyses were planned to be performed to allow early stopping for futility or efficacy. The first pre-specified interim analysis was planned at the time when 160 patients who completed the 4^th^ cycle visit (approximately 74 PFS events achieved), and if the observed ORR of the ARX788 group was <2% improved compared to LC, the trial would be early terminated. The second interim analysis was planned to be performed at two-thirds of the required PFS events had occurred to allow early stopping for efficacy. To maintain an overall α of 0.05, the Lan-DeMets α-spending function, to approximate O’Brien-Fleming was implemented based on the number of reported PFS events at each analysis. An independent data monitoring committee (IDMC) reviewed the trial data (including efficacy and safety) at the interim analyses and provided recommendations to the sponsor.

Efficacy was evaluated in the intent-to-treat (ITT) population (all patients randomized to ARX788 or LC, regardless of whether the assigned study treatment was received). The Kaplan-Meier method was used to estimate median PFS and the PFS probability at different time points. PFS was compared between ARX788 and LC using the stratified log-rank test; HR and 95% CI were estimated by Cox regression, with both treatment groups and stratification factors included in the model. The stratification factors used in the log-rank test and Cox regression were the same as the randomization stratification factors. Subgroup analyses, defined by demographics, baseline characteristics, and stratification factors, were conducted to evaluate the consistency of the PFS benefits across different patient subgroups. OS and DOR were analyzed similarly to PFS. Stratified Cochran Mantel-Haenszel test was used for the treatment group comparison in ORR, the difference in ORR and 95% CI was estimated based on normal approximation method. The safety population was defined as patients who received at least one dose of investigational drug and had post-baseline safety evaluations. For the assessment of safety, patients were analyzed based on the actual treatment received.

Statistical analyses were conducted using SAS (version 9.4). This trial is registered with Chinadrugtrials.org.cn, CTR20201708.

## Supplementary information

protocol of ACE-Breast-02

## Data Availability

The data are available from the corresponding author upon reasonable request.
