# Supplementary material for: ACE-Breast-02: a randomized phase III trial of ARX788 versus lapatinib plus capecitabine for HER2-positive advanced breast cancer
Source: Signal Transduct Target Ther. 2025 Feb 17;10:56. doi: 10.1038/s41392-025-02149-3 (PMC11830773; doi:10.1038/s41392-025-02149-3)
Supplement: Supplementary file 1 — protocol of ACE-Breast-02 [file 41392_2025_2149_MOESM1_ESM.docx]

**A Randomized, Open-Label, Positive Control, Phase III Clinical Trial of Recombinant Humanized Anti-HER2 Monoclonal Antibody-AS269 Conjugate (ARX788) in the Treatment of HER2-Positive Locally Advanced or Metastatic Breast Cancer**

|  | Leading Site: | Fudan University Shanghai Cancer Center |
| --- | --- | --- |
|  | Principal Investigator of the Leading Site: | HU Xichun |
|  | Sponsor: | NovoCodex Biopharmaceuticals Co., Ltd. |
|  | Address of the Sponsor: | 2/F, Auxiliary Building, Environmental Protection Center, No. 58 Changhe Road, Binhai New City, Shaoxing |
|  | Contact Person/Contact Number of the Sponsor: | LIANG Xuejun/(0575)85211809 |
|  | Protocol No.: | ZMC-ARX788-211 |
|  | Protocol Version/Version Date: | 3.0/July 14, 2020 |
|  | | |

**Confidentiality Statement**

The ownership of the information contained in this Protocol belongs to NovoCodex Biopharmaceuticals Co., Ltd. This document contains confidential information belonging to the applicant of the trial. By receiving or accessing this Protocol, you agree to keep the information confidential, and may not copy or disclose it to others (unless required by applicable laws) or use it for unauthorized purposes, unless otherwise agreed in writing. Any violation or suspected violation of this principle of confidentiality shall be reported immediately to the applicant of the trial.

# Signature Page of the Protocol

We have read the Protocol of “A Randomized, Open-Label, Positive Control, Phase III Clinical Trial of Recombinant Humanized Anti-HER2 Monoclonal Antibody-AS269 Conjugate (ARX788) in the Treatment of HER2-Positive Locally Advanced or Metastatic Breast Cancer”, with Protocol No. ZMC-ARX788-211 and Version No. 3.0 (Version Date: July 14, 2020). We have confirmed this Protocol and agree to conduct this clinical trial in accordance with the Protocol and the Good Clinical Practice.

We will keep this Protocol and related contents confidential.

Sponsor: NovoCodex Biopharmaceuticals Co., Ltd.

Signature of Trial Lead: [signature]

Date: (Month) (Day), (Year)

# Signature Page of the Protocol

| Protocol Title: | A Randomized, Open-Label, Positive Control, Phase III Clinical Trial of Recombinant Humanized Anti-HER2 Monoclonal Antibody-AS269 Conjugate (ARX788) in the Treatment of HER2-Positive Locally Advanced or Metastatic Breast Cancer |
| --- | --- |
| Trial No.: | ZMC-ARX788-211 |
| Investigational Product: | Recombinant Humanized Anti-HER2 Monoclonal Antibody-AS269 Conjugate (ARX788) |
| Protocol Version No.: | 3.0 |
| Version Date: | July 14, 2020 |

**Protocol writing:**

|  |  |  |
| --- | --- | --- |
| Name: DU Qingqing  Position: Senior Medical Writing Specialist |  | Date: (MM/DD/YYYY)  Company: Nanjing CR Medicon Pharmaceutical Technology Co., Ltd. |

# Principal Investigator’s Statement

1. I agree to personally participate in or direct this clinical trial.
2. I have received the Investigator’s Brochure and I have been informed of the preclinical studies of the investigational product.
3. I have read the Protocol of “A Randomized, Open-Label, Positive Control, Phase III Clinical Trial of Recombinant Humanized Anti-HER2 Monoclonal Antibody-AS269 Conjugate (ARX788) in the Treatment of HER2-Positive Locally Advanced or Metastatic Breast Cancer”, with Protocol No. ZMC-ARX788-211 and Version No. 3.0 (Version Date: July 14, 2020). The study will be conducted in accordance with the ethical and scientific principles stipulated in the Declaration of Helsinki and the GCP. I agree to conduct this clinical trial in accordance with the design and stipulations outlined in this Protocol, and to make changes to the Protocol only after notification to the Sponsor. Consent of the Ethics Committee is required before implementation, unless measures are necessary to protect the safety, rights and interests of the participants.
4. I will ensure that all participants sign the written informed consent form before entering the trial in accordance with the GCP requirements.
5. I will be responsible for making medical decisions related to the clinical trial to ensure that participants receive timely and appropriate treatment in the event of an adverse event during the trial. I will also document and report serious adverse events in accordance with the relevant national regulations.
6. I guarantee that the data are entered into the study medical records in a truthful, accurate, complete, and timely manner. I will accept the monitoring and audit by the monitor or auditor dispatched by the Sponsor and the audit and inspection by the drug regulatory authority to ensure the quality of the clinical trial.
7. I undertake to keep confidential the participant information and related matters. I have been informed that I will bear the legal liability arising from any breach of my commitments.
8. I agree to disclose my full name and occupation to the Sponsor, and to disclose the expenses related to the clinical trial upon request. I agree to prohibit commercial and economic practices related to this trial.

Affiliation of Principal Investigator:

Print Name of Principal Investigator:

Signature of Principal Investigator:

Date: (Month) (Day), (Year)

# Table of Contents

[Signature Page of the Protocol 2](#_Toc98520245)

[Signature Page of the Protocol 3](#_Toc98520246)

[Principal Investigator’s Statement 4](#_Toc98520247)

[Table of Contents 5](#_Toc98520248)

[Abbreviations 9](#_Toc98520249)

[Protocol Synopsis 11](#_Toc98520250)

[Body of the Trial Protocol 26](#_Toc98520251)

[1 Study Background 26](#_Toc98520252)

[1.1 Disease Background 26](#_Toc98520253)

[1.1.1 Diagnosis and Staging of Breast Cancer 26](#_Toc98520254)

[1.1.2 HER2 Expression and Testing in Breast Cancer 26](#_Toc98520255)

[1.1.3 Current Status of Breast Cancer Treatment 27](#_Toc98520256)

[1.2 Current Status of HER2-Targeted Drug Therapy in Metastatic Breast Cancer 28](#_Toc98520257)

[1.2.1 Trastuzumab 28](#_Toc98520258)

[1.2.2 Pertuzumab 29](#_Toc98520259)

[1.2.3 Ado-trastuzumab Emtansine (T-DM1) 30](#_Toc98520260)

[1.2.4 Lapatinib 31](#_Toc98520261)

[1.2.5 Pyrotinib Maleate 31](#_Toc98520262)

[1.3 Information of Investigational Product 33](#_Toc98520263)

[1.3.1 General Information of Investigational Product 33](#_Toc98520264)

[1.3.2 Pharmacodynamic Studies 33](#_Toc98520265)

[1.3.3 Non-Clinical Pharmacokinetic Studies 34](#_Toc98520266)

[1.3.4 Toxicologic Study 36](#_Toc98520267)

[1.3.5 Previous Clinical Trials 41](#_Toc98520268)

[1.4 Risk Benefits Analysis 46](#_Toc98520269)

[2 Study Objectives 48](#_Toc98520270)

[2.1 Primary Objective 48](#_Toc98520271)

[2.2 Secondary Objectives 49](#_Toc98520272)

[3 Trial Design 49](#_Toc98520273)

[3.1 Overall Design 49](#_Toc98520274)

[3.2 Selection of Trial Population and Justification 50](#_Toc98520275)

[3.3 Selection of Administration Dose and Justification 51](#_Toc98520276)

[3.4 Toxicity-Based Dose Modification 53](#_Toc98520277)

[3.5 Population Pharmacokinetic Study 55](#_Toc98520278)

[3.6 Immunogenicity 55](#_Toc98520279)

[3.7 Blinding and Unblinding 56](#_Toc98520280)

[3.8 Randomization 56](#_Toc98520281)

[3.9 End of Trial 56](#_Toc98520282)

[4 Study Population 56](#_Toc98520283)

[4.1 Inclusion Criteria 56](#_Toc98520284)

[4.2 Exclusion Criteria 58](#_Toc98520285)

[4.3 Criteria for Treatment Discontinuation/Withdrawal 60](#_Toc98520286)

[4.4 Criteria for Early Discontinuation of Trial 61](#_Toc98520287)

[4.5 Participant Assignment and Number 61](#_Toc98520288)

[5 Investigational Product Information 61](#_Toc98520289)

[5.1 Basic Information of Investigational Product 61](#_Toc98520290)

[5.2 Information on Other Investigational Products 62](#_Toc98520291)

[5.2.1 Control Drug - Lapatinib 62](#_Toc98520292)

[5.2.2 Control Drug - Capecitabine 62](#_Toc98520293)

[5.3 Investigational Product Administration 63](#_Toc98520294)

[5.4 Packaging and Labeling of Investigational Product 63](#_Toc98520295)

[5.5 Management of Investigational Products 63](#_Toc98520296)

[5.6 Concomitant Medications 64](#_Toc98520297)

[6 Study Procedures 65](#_Toc98520298)

[6.1 Screening Period (D-28 - D-1) 65](#_Toc98520299)

[6.2 Treatment Period - Day 1 (C1D1) 67](#_Toc98520300)

[6.3 Treatment Period - Day 7 (C1D7) 67](#_Toc98520301)

[6.4 Treatment Period - Day 14 (C1D14) 68](#_Toc98520302)

[6.5 Treatment Period - (C2D1 - discontinuation) 68](#_Toc98520303)

[6.6 Follow-up Period After Treatment Discontinued (within 28+7 days after discontinuation) 70](#_Toc98520304)

[6.7 Long-term follow-up period 72](#_Toc98520305)

[6.8 Unscheduled Visit 73](#_Toc98520306)

[6.9 Population Pharmacokinetic Study 73](#_Toc98520307)

[7 Study Evaluation 73](#_Toc98520308)

[7.1 Efficacy Evaluation 73](#_Toc98520309)

[7.1.1 Anti-tumor Efficacy Evaluation 73](#_Toc98520310)

[7.1.2 Telephone Visit 75](#_Toc98520311)

[7.1.3 Efficacy Evaluation Indicators 75](#_Toc98520312)

[7.2 Safety Evaluation Indicators 76](#_Toc98520313)

[7.2.1 Vital Signs 76](#_Toc98520314)

[7.2.2 Physical Examination 77](#_Toc98520315)

[7.2.3 Weight Measurement 77](#_Toc98520316)

[7.2.4 12-Lead ECG 77](#_Toc98520317)

[7.2.5 Echocardiography 78](#_Toc98520318)

[7.2.6 ECOG Score 78](#_Toc98520319)

[7.2.7 Routine Laboratory Tests 78](#_Toc98520320)

[7.2.8 Serum Virology Testing 79](#_Toc98520321)

[7.2.9 Pregnancy Test 79](#_Toc98520322)

[7.3 Population Pharmacokinetic Evaluation 79](#_Toc98520323)

[7.4 Immunogenicity Evaluation 80](#_Toc98520324)

[7.5 Other Assessment 80](#_Toc98520325)

[7.5.1 Demographic Information 80](#_Toc98520326)

[7.5.2 Medical History 80](#_Toc98520327)

[7.5.3 Previous Medications and Concomitant Medications and Treatments 80](#_Toc98520328)

[8 Adverse Events 81](#_Toc98520329)

[8.1 Definition of Adverse Events 81](#_Toc98520330)

[8.2 Monitoring of Adverse Events 82](#_Toc98520331)

[8.3 Recording of Adverse Events 83](#_Toc98520332)

[8.4 Abnormal Laboratory Findings 83](#_Toc98520333)

[8.5 Severity of Adverse Events 84](#_Toc98520334)

[8.6 Adverse Event Causality Assessment 84](#_Toc98520335)

[8.7 Suspected Unexpected Serious Adverse Reactions 85](#_Toc98520336)

[8.8 Definition of Serious Adverse Events 85](#_Toc98520337)

[8.9 Safety Reporting 86](#_Toc98520338)

[8.10 Adverse Events of Special Interest 87](#_Toc98520339)

[8.11 Pregnancy Reporting 88](#_Toc98520340)

[8.12 Follow-up of Adverse Events 88](#_Toc98520341)

[9 Risk Management Plan 88](#_Toc98520342)

[10 Statistical Analysis 96](#_Toc98520343)

[10.1 Statistical Hypotheses and Sample Size Calculation 96](#_Toc98520344)

[10.2 Interim Analysis 96](#_Toc98520345)

[10.3 Statistical Analysis Population 98](#_Toc98520346)

[10.3.1 Intent-to-Treat (ITT) Analysis Set 98](#_Toc98520347)

[10.3.2 Per-Protocol Set (PPS) 98](#_Toc98520348)

[10.3.3 Safety Analysis Set (SS) 98](#_Toc98520349)

[10.3.4 Population PK Analysis Set 98](#_Toc98520350)

[10.3.5 Immunogenicity Analysis Set 98](#_Toc98520351)

[10.4 Statistical Analysis Methods 98](#_Toc98520352)

[10.4.1 Demographic Data and Other Baseline Characteristics 98](#_Toc98520353)

[10.4.2 Efficacy Analysis 98](#_Toc98520354)

[10.4.3 Safety Analysis 99](#_Toc98520355)

[10.4.4 Population Pharmacokinetic Analysis 99](#_Toc98520356)

[10.4.5 Immunogenicity Analysis 100](#_Toc98520357)

[11 Data Processing 100](#_Toc98520358)

[11.1 Raw Data and Original Documents 100](#_Toc98520359)

[11.2 Data Management 101](#_Toc98520360)

[11.3 Independent Data Monitoring Committee 102](#_Toc98520361)

[11.4 Independent Endpoint Review Committee 102](#_Toc98520362)

[12 Clinical Monitoring 102](#_Toc98520363)

[13 Quality Assurance and Control 105](#_Toc98520364)

[14 Ethical Standards and Informed Consent 105](#_Toc98520365)

[14.1 Review and Approval of Ethics Committee 105](#_Toc98520366)

[14.2 Informed Consent of Participants 106](#_Toc98520367)

[15 Changes in Trial Procedures 108](#_Toc98520368)

[15.1 Amendment to the Study Protocol 108](#_Toc98520369)

[15.2 Deviation from the Study Protocol 108](#_Toc98520370)

[15.3 Early Discontinuation of the Trial 108](#_Toc98520371)

[16 Data and Information Storage 108](#_Toc98520372)

[16.1 Confidentiality and Ownership of Trial Data 109](#_Toc98520373)

[16.2 Data Publication and Public Disclosure 109](#_Toc98520374)

[16.2.1 Publication Policy 109](#_Toc98520375)

[16.2.2 Public Disclosure Policy 109](#_Toc98520376)

[16.3 Study Data Retention 110](#_Toc98520377)

[17 Responsibilities of the Parties and Other Relevant Provisions 110](#_Toc98520378)

[18 Study Site and Trial Participants 113](#_Toc98520379)

[18.1 Sponsor 113](#_Toc98520380)

[18.2 Leading Site 113](#_Toc98520381)

[18.3 Data Management and Statistics Unit 113](#_Toc98520382)

[18.4 Contract Research Organization 113](#_Toc98520383)

[19 References 113](#_Toc98520384)

[Appendix 1 Clinical Trial Flow Chart 116](#_Toc98520385)

[Appendix 2 ECOG PS Score 121](#_Toc98520386)

[Appendix 3 Response Evaluation Criteria in Solid Tumors RECIST 1.1 Quick Reference 122](#_Toc98520387)

[Appendix 4 Management of Infusion-Related Reactions 129](#_Toc98520388)

[Appendix 5 New York Heart Association Functional Classification of Heart Failure 131](#_Toc98520389)

**List of Tables**

[Table 1 Summary of pharmacokinetic parameters in the first-in-human trial of ARX788 46](#_Toc98520390)

[Table 2 Analysis of accumulation factors of intact ADC pharmacokinetic parameters in human body of Chinese participants with advanced breast cancer treated with ARX788 49](#_Toc98520391)

[Table 3 Toxicity-based dose modification in participants in the ARX788 group 57](#_Toc98520392)

[Table 4 ARX788 dose modification levels 59](#_Toc98520393)

[Table 5 Sponsor and mailing address for SAE reporting 91](#_Toc98520394)

[Table 6 Treatment-related AEs in ≥ 2 participants by system organ class and preferred terms 93](#_Toc98520395)

[Table 7 SAEs summarized by MedDRA system organ class and preferred terms 95](#_Toc98520396)

[Table 8 Adverse events of special interest (AESIs) in the first-in-human trial in China 96](#_Toc98520397)

# Abbreviations

| **Abbreviation** | **Expanded Term** | **Abbreviation** | **Expanded Term** |
| --- | --- | --- | --- |
| ADA | Anti-drug antibody | IDMC | Independent Data Monitoring Committee |
| ADC | Antibody drug conjugate | IEC | Independent Ethics Committee |
| AE | Adverse event | IHC | Immunohistochemistry |
| A/G | Albumin/globulin | IMP | Investigational medicinal product |
| AKI | Acute kidney injury | INR | International normalized ratio |
| ALP | Alkaline phosphatase | IRC | Independent Review Committee |
| ALT | Alanine aminotransferase | ITT | Intent-to-treat analysis set |
| ANC | Absolute neutrophil count | LVEF | Left ventricular ejection fraction |
| AST | Aspartate aminotransferase | MedDRA | Medical Dictionary for Regulatory Activities |
| ARX788 | Recombinant humanized anti-HER2 monoclonal antibody-AS269 conjugate | MRI | Magnetic resonance imaging |
| C | Cycle | MTD | Maximum tolerated dose |
| CI | Confidence interval | NCI | National Cancer Institute |
| CK | Cytokeratin | NHP | Non-human primate |
| CL | Clearance | ORR | Objective response rate |
| C_max_ | Maximum concentration | OS | Overall survival |
| CNS | Central nervous system | PFS | Progression-free survival |
| CR | Complete response | PPS | Per protocol analysis set |
| CRO | Contract Research Organization | PR | Partial response |
| CSCO | Chinese Society of Clinical Oncology | PS | Performance status |
| CSR | Clinical study report | PTT | Partial thromboplastin time |
| CT | Computerized tomography | Q3W | Every 3 weeks |
| CTCAE | Common Terminology Criteria for Adverse Events | Q4W | Every 4 weeks |
| DCR | Disease control rate | QTc | QT corrected for heart rate |
| DLT | Dose-limiting toxicity | RDW | Red cell distribution width |
| DOR | Duration of response | RECIST | Response Evaluation Criteria in Solid Tumors |
| ECHO | Echocardiography | SAE | Serious adverse event |
| ECOG | Eastern Cooperative Oncology Group | SAF | Safety analysis set |
| eCRF | Electronic case report form | SAP | Statistical analysis plan |
| FAS | Full analysis set | SBP | Systolic blood pressure |
| FDA | Food and Drug Administration | Scr | Serum creatinine |
| FIH | First-in-human trial | SMC | Safety Monitoring Committee |
| FISH | Fluorescent in situ hybridization | SUSAR | Suspected unexpected serious adverse event |
| GCP | Good Clinical Practice | TG | Triglycerides |
| HER2 | Human epidermal growth factor receptor 2 | TP | Total protein |
| HNSTD | Highest non-severely toxic dose | T_max_ | Time to C_max_ |
| IB | Investigator’s Brochure | TTP | Time to progression |
| IC50 | Half-maximal (50%) inhibitory concentration | ULN | Upper limit of normal |
| ICF | Informed consent form | V_z_ | Volume of distribution |

# Protocol Synopsis

| **Basic Information of the Clinical Trial** | Registration Category: Therapeutic biological product  Clinical Trial Protocol No.: ZMC-ARX788-211  Protocol Version/Version Date: 3.0/July 14, 2020  Trial Phase: Phase III  Investigational Product: Recombinant Humanized Anti-HER2 Monoclonal Antibody-AS269 Conjugate (ARX788)  Basic Design: A randomized, open-label, positive control, phase III clinical trial  Sponsor: NovoCodex Biopharmaceuticals Co., Ltd. |
| --- | --- |
| **Study Title** | A Randomized, Open-Label, Positive Control, Phase III Clinical Trial of Recombinant Humanized Anti-HER2 Monoclonal Antibody-AS269 Conjugate (ARX788) in the Treatment of HER2-Positive Locally Advanced or Metastatic Breast Cancer |
| **Study Objective** | **Primary Objective**:  To evaluate the efficacy of ARX788 versus lapatinib in combination with capecitabine in the treatment of HER2-positive locally advanced or metastatic breast cancer based on progression-free survival (PFS) evaluated by the Independent Endpoint Review Committee (IRC).  **Secondary Objectives:**   1. To further evaluate the efficacy of ARX788 versus the control drug in the treatment of HER2-positive locally advanced or metastatic breast cancer based on the participant’s overall survival (OS), investigator-assessed PFS, objective response rate (ORR, including partial response [PR] and complete response [CR]), disease control rate (DCR), and duration of response (DOR) 2. To further observe the safety of ARX788 in participants with HER2-positive locally advanced or metastatic breast cancer 3. To evaluate the immunogenicity of ARX788 in participants with HER2-positive locally advanced or metastatic breast cancer 4. To evaluate the population pharmacokinetic (PK) profile of ARX788 in participants with HER2-positive locally advanced or metastatic breast cancer |
| **Trial Design** | **Overall Design:**  This trial is a randomized, open-label, positive control, phase III clinical trial of ARX788 in participants with HER2-positive locally advanced or metastatic breast cancer.  This trial plans to enroll 440 participants with HER2-positive locally advanced or metastatic breast cancer who have previously received treatment with taxane and trastuzumab, and the participants will be randomized in a 1:1 ratio to the investigational product group or the control drug group to receive the investigational product monotherapy or lapatinib in combination with capecitabine.  ARX788 will be administered at a dose of 1.5 mg/kg once every 3 weeks (Q3W) in the investigational product group, and standard doses of lapatinib and capecitabine will be administered in the control drug group. All participants will receive long-term administration until intolerable toxicity or disease progression or death or voluntary withdrawal or the end of this trial (defined as trial completion or early trial discontinuation). If, after obtaining updated information on additional clinical trials of ARX788, the Investigator and Sponsor agree that other doses may have better efficacy and a manageable safety profile, some or all of the participants in the investigational product group may be administered with other dosing regimens.  During the treatment period, all participants will be assessed for anti-tumor efficacy every six weeks based on their disease status as determined by the IRC and the Investigator respectively as per RECIST1.1 criteria until disease progression or death or refusal to come to the hospital for follow-up or the end of the trial (including the completion and early discontinuation of the trial), whichever occurs first. After a participant experiences disease progression or refuses to come to the hospital for follow-up, the Investigator will conduct a telephone visit every 3 months (30 days per month) to obtain the participant’s survival information and whether he/she is receiving other anti-tumor treatments. PK samples will be collected and tested in participants in the investigational product group to assess the population PK profile of ARX788 in patients with HER2-positive advanced breast cancer. If conditions permit, population PK samples should be collected and tested in all participants in the investigational product group. All participants in the investigational product group will receive immunogenicity testing to assess the potential effect of the immunizing antigen production on the efficacy and safety of the investigational product.  In this trial, the primary analysis of PFS will be conducted when 335 PFS events assessed by the Independent Review Committee (IRC) occur. Two interim analyses will be set up during the trial. The first interim analysis will be performed when 160 participants complete the Cycle 4 visit, and futility will be determined based on the ORR difference to decide whether to continue the enrollment; the second interim analysis will be performed when 224 (2/3) IRC-assessed PFS events occur, and superiority test and sample size re-estimation will be performed.  **Selection of Trial Population and Justification**  This trial plans to enroll HER2-positive locally advanced or metastatic breast cancer participants, i.e., participants who have received ≤ two lines of chemotherapy (excluding hormone therapy) for recurrent or metastatic diseases before enrollment. HER2-positive is defined as FISH+ or IHC 3+.  Breast cancer is the most common malignant tumor in women worldwide. In 2015, there were about 272,000 new cases of breast cancer and about 70,000 deaths in China. About 3% to 10% of new breast cancer cases each year have distant metastases at the time of diagnosis. Among early-stage patients, 30%-40% may develop into advanced breast cancer, with a 5-year survival rate of only 20% and an overall median survival time of 2-3 years. HER2 overexpression in 15-20% of breast cancer patients is an important adverse prognostic factor for this disease.  Since trastuzumab was approved for treatment of HER2-positive breast cancer, the treatment of such patients has been significantly improved. Trastuzumab binds to the extracellular ligand-binding domain IV of HER2 protein to exert its anti-tumor effect by blocking HER2-mediated mitogenic signaling. The study data on the use of trastuzumab in the first-line treatment of metastatic breast cancer have shown that the time to progression (TTP) of the trastuzumab combined with chemotherapy group was significantly better than that of the chemotherapy group (7.2 vs 4.5 months). However, the study data have also suggested that most patients with metastatic breast cancer developed drug resistance and disease progression after receiving trastuzumab treatment, presenting higher requirements for targeted therapy in patients with HER2-positive metastatic breast cancer.  T-DM1 (Kadcyla^®^) is an antibody-drug conjugate (ADC) composed of trastuzumab combined with small molecule microtubule inhibitor DM1. T-DM1 produces the same physiological activity as trastuzumab, and blocks HER2-mediated mitogenic signaling by binding to extracellular ligands of HER2 protein. T-DM1 also enters the cells via endocytosis by target binding to HER2 and releases the active cytotoxic payload DM1 within HER2-expressing cells after lysosomal degradation, thus greatly reducing the toxicity produced by whole-body exposure of DM1. T-DM1 was approved by the U.S. Food and Drug Administration (FDA) on February 22, 2013 for the treatment of HER2-positive metastatic breast cancer after trastuzumab and taxane chemotherapy. In 2018, the Guidelines for the Diagnosis and Treatment of Breast Cancer (2018.V1) of the Chinese Society of Clinical Oncology (CSCO) pointed out that T-DM1 is the international standard second-line anti-HER2 treatment regimen. T-DM1 has not yet been marketed in China, but patients should be encouraged to enter clinical trials to achieve the best survival benefit.  Similar to the mechanism of action of T-DM1, the investigational product ARX788 (or known as WBP265 ADC) is an antibody-drug conjugate composed of anti-HER2 monoclonal antibody and toxin small molecule AS269. The anti-HER2 monoclonal antibody can specifically bind to human HER2 and AS269 is a highly effective microtubule inhibitor that inhibits cell growth. The results from the phase I trial on the safety, tolerability and pharmacokinetics of ARX788 monotherapy in Chinese patients with HER2 advanced breast cancer showed a favorable safety profile of the investigational product and a tumor treatment response in the target dose group following efficacy assessment.  Taken together, ARX788 is expected to have a good therapeutic effect in patients with HER2-positive locally advanced or metastatic breast cancer, and can provide a new option for the treatment of such patients.  **Selection of Administration Dose and Justification:**  The phase I clinical trials have been conducted in Australian and Chinese participants with HER2-positive advanced breast cancer, respectively, to fully explore the tolerability, safety, efficacy and pharmacokinetic profile of ARX788. The preliminary results from the two phase I trials are summarized as follows:   - Phase I trial in Australia: In the dose-escalation, phase I clinical trial of ARX788 monotherapy in the treatment of HER2-positive (in situ hybridization [FISH]+ or IHC 3+) advanced breast cancer (clinicaltrial.gov registration number NCT02512237) in Australia, the dose groups explored and the corresponding number of participants were 0.33 mg/kg (1 participant), 0.66 mg/kg (1 participant), 1.3 mg/kg (3 participants), 2.20 mg/kg (3 participants), and 2.9 mg/kg (1 participant), respectively. The efficacy results showed that 1 participant (100%) in the 0.66 mg/kg group, 1 participant (33%) in the 1.3 mg/kg group, and 1 participant (33%) in the 2.2 mg/kg group were determined to have partial response (PR) after efficacy assessment. The dose-limiting toxicity (DLT) first appeared in the 2.9 mg/kg dose group, which was Grade 3 mucosal inflammation. Delayed toxicity occurred after 4 to 5 cycles of drug administration in the ≥1.3 mg/kg dose groups (the delayed toxicity was subsequently determined as delayed DLT):   - One participant in the 1.3 mg/kg group experienced Grade 2 non-infectious pneumonia at Cycle 4 treatment that improved after drug discontinuation and steroid therapy;   - Two participants in the 2.2 mg/kg group developed Grade 2 and Grade 5 non-infectious pneumonia after 4 to 5 cycles of treatment, respectively. - Phase I trial in China: The phase I clinical trial of ARX788 monotherapy in the treatment of participants with HER2-positive advanced breast cancer in China (drug clinical trial registration number CTR20171162) is ongoing, with 51 (100%) of the 51 participants treated with at least one study drug meeting criteria for efficacy and safety assessments as of January 20, 2020. The dosing regimens completed/being explored in the study and the corresponding sample sizes are as follows: - 0.33 mg/kg Q3W (3 participants); - 0.66 mg/kg Q3W (3 participants); - 0.88 mg/kg Q3W (3 participants); - 1.10 mg/kg Q3W (8 participants); - 1.30 mg/kg Q3W (12 participants); - 1.50 mg/kg Q3W (11 participants); - 0.88 mg/kg Q4W (4 participants); - 1.10 mg/kg Q4W (3 participants); - 1.30 mg/kg Q4W (4 participants).   There were no DLTs among the participants in the groups that had completed dose limiting toxicity (DLT) assessment. No adverse events leading to the death of participants were reported, and 4 participants experienced Grade 3 or above treatment emergent adverse events (TEAEs) related to the investigational product: 1 in the 0.88 mg/kg Q3 group, 2 in the 1.1 mg/kg Q3W group, and 1 in the 1.5 mg/kg Q3W group. Of the 49 participants who experienced TEAEs related to the investigational product, 91.8% had a maximum severity of Grade 2, and there was no apparent increasing trend in the incidence of AEs as the dose escalated.  The best response in participants who have been assessed was PR, and PR did not occur in participants in the 0.66 mg/kg Q3W and below dose groups. The PR rate was 33.3% (1/3 participants) in the 0.88 mg/kg Q3W group, 37.5% (3/8 participants) in the 1.1 mg/kg Q3W group, 50% (6/12 participants) in the 1.3 mg/kg Q3W group, and 63.6% (7/11 participants) in the 1.5 mg/kg Q3W group. The PR rate showed a tendency to increase with dose. Based on the above safety and efficacy data, the dosing regimen of ARX788 in this phase III clinical trial is preliminarily set as 1.5 mg/kg Q3W. Alternative dosing regimens may be used for some or all participants in this trial if, after obtaining updated information from other clinical trials of the study drug, the Investigator and the Sponsor mutually agree that alternative dosing and/or dosing regimens may have better efficacy and a manageable safety profile.  **Toxicity-Based Dose Modification:**  Participants who experience clinically significant adverse events (AEs), or intolerable toxicity, may receive necessary interventions and supportive treatment. The Investigator will determine whether a participant in the investigational product group requires dose modification of ARX788, delayed dosing, or withdrawal from the trial based on the grade of adverse events occurring after treatment, recovery, and correlation with ARX788 as per the relevant criteria specified in the Protocol. Toxicity-based dose modifications for participants in the ARX788 group can be found in the following table:   \| **AE** \| **Grade** \| **Dose Modification**  **& Adjustment Principle** \| \| --- \| --- \| --- \| \| Elevated transaminase (AST/ALT) \| Grade 2 (> 3, ≤ 5 times the upper limit of normal [ULN]) \| No dose modification \| \| Grade 3 (> 5, ≤ 20 times ULN) \| Withhold ARX788 until AST/ALT level recovers to ≤ Grade 2, and continued at a reduced dose level \| \| Grade 4 (> 20 times ULN) \| Permanently discontinue treatment with ARX788 \| \| Elevated bilirubin \| Grade 2 (> 1.5, ≤ 3 times ULN) \| Withhold ARX788 and resume at the original dose when the total bilirubin level recovers to ≤ Grade 1 \| \| Grade 3 (> 3, ≤ 10 times ULN) \| Withhold ARX788 and continue at a reduced dose when the total bilirubin level recovers to ≤ Grade 1 \| \| Grade 4 (> 10 times ULN) \| Permanently discontinue treatment with ARX788 \| \| Platelets decrease \| Grade 2 (50,000-75,000/mm^3^) \| Withhold ARX788 if the condition does not recover to ≤ Grade 1 or worsens to Grade 3 within 2 weeks. And it continues at the original dose if the platelet level recovers to ≤ Grade 1 (≥ 75,000/mm^3^). \| \| Grade 3 (25,000-50,000/mm^3^)  Grade 4 (< 25,000/mm^3^) \| Withhold ARX788 and continue at the original dose when the platelet level recovers to ≤ Grade 1 (≥ 75,000/mm^3^).  Withhold ARX788 and continue at a reduced dose when the platelet level recovers to ≤ Grade 1 (≥ 75,000/mm^3^). \| \| Left ventricular dysfunction \| Symptomatic congestive cardiac failure  LVEF < 40% \| Permanently discontinue ARX788 use  Withhold ARX788 use, and permanently discontinue ARX788 use if LVEF < 40% after re-assessment of LVEF within 3 weeks. \| \| LVEF of 40% to ≤45%, ≥10% reduction from baseline \| Withhold ARX788 use, and permanently discontinue ARX788 use if LVEF does not recover to within 10% from baseline after re-assessment of LVEF within 3 weeks. \| \| LVEF of 40% to ≤45%, <10% reduction from baseline \| Continue with the original dose of ARX788 and intensify LVEF monitoring. \| \| LVEF > 45% \| Continue with the original dose of ARX788 \| \| Pulmonary toxicity (interstitial lung disease or pneumonitis, etc.) \| Grade 1~2 \| ARX788 should be on hold, as determined by the investigator until the event recovers to ≤ Grade 1 within 12 weeks. Otherwise, permanently discontinue ARX788 treatment. \| \| Grade 3 or 4 \| Permanently discontinue ARX788 treatment. \| \| Ocular toxicity (keratitis or dry eye, etc.) \| Grade 2 \| If a Grade 2 ocular adverse event persists for more than 2 weeks, continue ARX788 administration at a reduced dose subsequently at the Investigator’s discretion. If the Grade 2 ocular adverse event does not resolve after the dose reduction, withheld the administration of ARX788, and if the adverse event level recovers to ≤ Grade 1 within 12 weeks, continue administration at a reduced dose at the Investigator’s discretion. \| \| Grade 3 \| Withhold ARX788 use and continue administration at a lower dose if the adverse event levels recover to ≤ Grade 1 within 12 weeks, as determined by the Investigator. If a Grade ≥2 ocular adverse event occurs after dose reduction, permanently discontinue ARX788 treatment and the participant withdraws from the trial. \| \| Grade 4 \| Permanently discontinue ARX788 treatment and the participant withdraws from the trial. \| \| Other adverse events \|  \| The Investigator determines ARX788 dose modification, delayed administration or withdrawal from the trial according to the participant’s clinical symptoms. \|   For the participants in the control drug group, refer to the package inserts of lapatinib and capecitabine as well as clinical routine procedures for the management of TEAEs, dose modification or delayed administration.  If the participant experiences a dose reduction due to toxicity during the treatment period, then the reduced dose can be used for subsequent continuous treatment. Dose recovery is not allowed after the recovery of toxicity in the investigational product group participants and the control drug group participants. Participants are allowed a maximum of 3 dose level adjustments in the investigational product group, with the following dose level adjustments referenced:   \| **Dose level** \| **Dose** \| \| --- \| --- \| \| 0 \| 1.5 mg/kg Q3W \| \| -1 \| 1.3 mg/kg Q3W \| \| -2 \| 1.1 mg/kg Q3W \| \| -3 \| 0.88 mg/kg Q3W \| \| Dose modification at lower level \| Permanent discontinuation \|   **Population Pharmacokinetic Study:**  PK data from the phase I study of ARX788 in the treatment of advanced breast cancer showed that exposure to intact ADC, total antibody and metabolite pAF-AS269 in the serum of participants administered at 1.5 mg/kg Q3W increased with doses of ADC (ARX788) in both Cycle 1 and Cycle 3, with intact ADC at T_1/2_ of 154 h in Cycle 3. PK samples will be collected and tested in participants in the investigational product group to assess the population PK profile of ARX788 in patients with HER2-positive advanced breast cancer. If conditions permit, population PK samples should be collected and tested in all participants in the investigational product group.  **Immunogenicity:**  The ARX788 contains an anti-HER2 monoclonal antibody, and participants with locally advanced or metastatic breast cancer enrolled in this trial have previously received trastuzumab targeted therapy. Therefore, some participants may have corresponding antibodies against trastuzumab in the body before ARX788 treatment, and antibodies may also be produced after ARX788 treatment. Antibodies before and after treatment may affect the efficacy and safety of treatment. Therefore, blood samples will be collected during the screening period and at different times after drug administration for immunogenicity study in participants in the investigational product group, and whether there is a potential link between the antibody production and the safety and efficacy will be analyzed.  In the trial, blood samples will be collected from all participants in the investigational product group for immunogenicity study before the first dose, every 2 treatment cycles after the first dose, and at the last visit. |
| **Study Population** | HER2-positive locally advanced or metastatic breast cancer participants, who have received ≤ two lines of chemotherapy (excluding hormone therapy) for recurrent or metastatic diseases before enrollment. HER2-positive is defined as FISH+ or IHC 3+. |
| **Inclusion Criteria** | **Any participant who meets all of the following criteria can be enrolled:**   1. The participant or his/her legal representative agree to voluntarily sign the informed consent form in writing; 2. 18 to 75 years old (including upper and lower limits), male or female; 3. Has a cytological or histological diagnosis of breast cancer (BC), with unresectable locally advanced, recurrent or metastatic BC; 4. Has previously received ≤ two lines of chemotherapy (excluding hormone therapy) for recurrent or metastatic BC; 5. Must meet at least one of the following conditions:    1. (Neoadjuvant) adjuvant therapy, receiving treatment containing trastuzumab (or marketed trastuzumab biosimilars) ± pertuzumab for ≥ 9 weeks, with disease recurrence or progression during treatment or within 12 months after the end of treatment;    2. Recurrent or metastatic disease treated with at least one treatment regimen containing trastuzumab (or marketed trastuzumab biosimilars) ± pertuzumab, with disease progression during or after treatment. 6. Has previously received treatment with taxane; 7. Has at least one measurable target lesion as per RECIST1.1 criteria; 8. Can provide tissue samples for HER2 testing that are determined to be qualified by the central laboratory during the screening period; 9. Tissue samples determined to be HER2 positive (defined as IHC3+ or FISH+) by central laboratory testing; 10. ECOG Performance Status Score of 0-1; 11. LVEF ≥ 50% as shown by echocardiography during the screening period; 12. Has recovered from any AE (≤ Grade 1) related to prior surgery and prior cancer treatment, except for the following: a. alopecia; b. pigmentation; c. long-term toxicity caused by radiotherapy, which cannot be recovered based on the assessment of the Investigator; d. Grade 2 or lower neurotoxicity caused by platinum; e. hemoglobin 90-100 g/L (including boundary values). 13. Adequate bone marrow, liver, kidney and coagulation function (please refer to the upper limit of normal of each site):     - Bone marrow (no blood transfusion or use of adjuvant drugs to increase white blood cells and platelets within 2 weeks prior to screening):       - Absolute neutrophil count (ANC) ≥ 1.5 × 10^9^/L;       - Platelet ≥ 100 × 10^9/^L;       - Hemoglobin ≥ 90 g/L;     - Liver function:  - Bilirubin total ≤ 1.5 times the upper limit of normal (ULN); - Alanine aminotransferase (ALT) and aspartate aminotransferase (AST) ≤ 3 times the ULN, alkaline phosphatase (ALP) ≤ 2.5 times the ULN; ALT and AST ≤ 5 times the ULN in the presence of liver metastasis, and ALP ≤ 5 times the ULN in the presence of bone metastasis; - Renal function: Creatinine ≤ 1.5 times the ULN; - Coagulation: International normalized ratio (INR) ≤ 1.5 times the ULN, and activated partial thromboplastin time (APTT) ≤ 1.5 times the ULN;  1. Expected survival ≥ 3 months. |
| **Exclusion Criteria** | **Any participant who meets any of the following criteria can not be enrolled:**   1. Has known history to be allergic to any active ingredient or excipient of ARX788; a clear history of allergy to protein drugs; a history of specific allergies (asthma, rheumatism, eczematous dermatitis); or has experienced other serious allergic reactions and is not suitable for treatment with ARX788 as determined by the Investigator; 2. Has known hypersensitivity or delayed allergic reactions to certain components of capecitabine and lapatinib or similar drugs, or known contraindications to capecitabine, mainly including previous severe and unexpected reactions to fluoropyrimidine or known allergy to fluorouracil and known complete deficiency of dihydropyrimidine dehydrogenase (DPD) activity; 3. Has previously used any treatment regimen containing capecitabine and/or anti-HER2 TKI, except for any of the following:    1. (Neoadjuvant) adjuvant therapy containing capecitabine and/or anti-HER2 tyrosine kinase inhibitor (TKI), with disease recurrence more than 12 months after completion of full treatment with capecitabine and/or anti-HER2 TKI. The neoadjuvant treatment regimen must be effective, which refers to clinical CR or PR or pathological CR. In addition, patients have not been exposed to capecitabine or anti-HER2 TKI during the recurrence or metastasis setting;    2. Recurrent or metastatic disease, treated effectively with a regimen containing capecitabine and/or anti-HER2 TKI, but discontinued not for disease progression or drug-related adverse reaction and at a period of more than 6 months with no evidence of disease progression. Moreover, patients have not been exposed to capecitabine or anti-HER2 TKI during the (neoadjuvant) adjuvant treatment phase. 4. Has previously received T-DM1 or other HER2-ADC drugs; 5. Suffered from another malignant tumor in the past 5 years, except for cervical cancer in situ or non-melanoma skin cancer that has received curative treatment; 6. Has primary central nervous system (CNS) malignancy or CNS metastasis that has failed local treatment, except that patients who have asymptomatic brain metastasis, or have stable clinical symptoms that do not require steroids and other treatment for brain metastases for ≥ 28 days prior to the first administration of the investigational product are allowed to be enrolled; 7. Has interstitial lung disease requiring steroid therapy, a history of drug-induced interstitial lung disease, a history of radiation pneumonitis, or any evidence indicating clinically active interstitial lung disease; 8. Has any eye disease that require medical intervention such as keratitis, corneal disorder, retinal disorder or active eye infection; 9. Is unwilling or unable to stop wearing corneal contact lens during the trial; 10. Has cardiac insufficiency, including but not limited to congestive cardiac failure, transmural myocardial infarction, angina pectoris requiring drug therapy, clinically significant heart valve disorders, and high-risk arrhythmia, or clinically significant QTc abnormalities in the ECG examination during the screening period (QTc > 450 msec [male] or QTc > 470 msec [female] corrected on ECG at rest); 11. Uncontrolled hypertension (at rest: systolic blood pressure > 160 mmHg or diastolic blood pressure > 100 mmHg); 12. Has evidence of severe or uncontrollable systemic diseases (e.g., unstable or uncompensated respiratory, cardiac, hepatic, or renal disease) as determined by the Investigator; 13. Has used chemotherapy, radiotherapy or immunotherapy within 4 weeks prior to the first administration of the investigational product (the use of physiological replacement dose of corticosteroid [prednisone or equivalent < 15 mg/day] is permitted); 14. Has received breast cancer endocrine therapy within 2 weeks before the first administration of the investigational product; 15. Has received palliative radiotherapy for bone metastases within 2 weeks prior to the first administration of the investigational product; 16. Prior exposure to anthracyclines accumulated to doses of:     - Doxorubicin or liposomal doxorubicin > 500 mg/m^2^;     - Epirubicin > 900 mg/m^2^;     - Mitoxantrone > 120 mg/m^2^;     - Others (i.e., liposomal doxorubicin or other anthracyclines > the equivalent of 500 mg/m^2^ of doxorubicin);     - If more than one anthracycline is used, then the cumulative dose must not exceed the equivalent of 500 mg/m^2^ of doxorubicin. 17. Has any uncontrolled infection, or other situations that may limit trial compliance or interfere with the evaluation; 18. Is positive for hepatitis B surface antigen and HBV DNA ≥ ULN, or positive for any one of hepatitis C virus antibody, syphilis spirochete antibody, or human immunodeficiency virus antibody test result; 19. Plans to receive major surgical treatment or experiences severe traumatic injury within 2 weeks before the first use of the investigational product or during the trial; 20. A pregnant or breastfeeding female participant; 21. A woman of childbearing potential (WOCBP) (WOCBPs include: any woman participant who has had her menarche, and has not received successful artificial sterilization surgery [hysterectomy, bilateral tubal ligation, or bilateral oophorectomy] or has not menopausal); or a male participant who is unwilling or unable to use an acceptable method of contraception throughout the entire treatment period of this trial and within 8 months after the last dose of the investigational product; 22. Participated in any other clinical trial and used any other investigational product within 4 weeks prior to the first administration of the investigational product; 23. Has any mental or cognitive disorder that may restrict his/her understanding and execution of the informed consent form; 24. Other conditions that the Investigator considers inappropriate for participation in this trial, such as poor compliance. |
| **Criteria for Treatment Discontinuation/Withdrawal** | During the trial, a participant may voluntarily withdraw from the study due to reasons such as loss to follow-up or withdrawal of consent and request for withdrawal. The Investigator may allow the participant to discontinue the trial treatment or even withdraw from the trial if the following occurs:   1. The Investigator considers that the participant should discontinue the investigational product treatment for safety considerations (e.g., the participant experiences an intolerable adverse events); 2. The participant is pregnant; 3. The participant experiences disease progression; 4. The participant experiences a significant protocol violation that, in the opinion of the Investigator, significantly affects the evaluation of the primary endpoints of this trial, and the Investigator believes that the participant should discontinue treatment; 5. Other conditions that the Investigator determines to make the participant unfit to stay in the trial. |
| **Criteria for Early termination of Trial** | Early discontinuation of the trial means that the clinical trial has not yet been completed for all participants according to the Protocol and the entire trial or a part of the trial is discontinued midway. The purpose of trial discontinuation is mainly to protect the rights and interests of the participants, ensure the quality of the trial, and avoid unnecessary economic losses.  Under normal circumstances, the trial will not be prematurely terminated at will. However, the entire trial or a part of the trial (such as the trial in a site) may be prematurely terminated if any of the following occurs:   1. The total sample size for competitive enrollment has met the requirements of the trial, but the site has not completed the planned enrollment as contracted; 2. The investigator at the site cannot follow the Protocol, GCP, etc.; 3. Access to new information leads to an unfavorable risk-benefit evaluation of ARX788, including sufficient evidence to suggest a lack of efficacy or an unacceptable safety profile; 4. The collaborating institution does not consider it appropriate to continue this trial due to medical, ethical or commercial reasons, etc.; 5. The enrollment of participants is very poor and it is impossible to complete the trial in an acceptable time frame; 6. The National Medical Products Administration or the Ethics Committee orders the discontinuation of the trial for some reason.   Any early discontinuation of the clinical trial shall be promptly notified in writing to all parties (Sponsor, Research Institution, Ethics Committee, clinical trial institution and administrative authority). |
| **Investigational Product Information** | **Basic Information of Investigational Product**  ARX788 is an antibody-drug conjugate formed by covalent binding of a humanized anti-HER2 monoclonal antibody and AS269 (small molecule cytotoxin drug), and the conjugation ratio is fixed at 1:2. The investigational product has two dosage forms, namely, injection solution and sterile powder for injection. In this trial, only the sterile powder for injection is used. The main drug information is as follows:   - **Name:** Recombinant humanized anti-HER2 monoclonal antibody-AS269 conjugate for injection - **Code**: ARX788 - **Strength**: 50 mg/vial - **Dosage and administration**: Intravenous infusion, 1.5 mg/kg, Q3W - **Storage conditions**: 2°C-8°C, protected from light - **Transportation and storage conditions**: Cold chain transportation at 2°C-8°C - **Shelf-life**: Tentatively 18 months - **Excipients**: Histidine, histidine hydrochloride, trehalose and polysorbate 80 - **Provider**: NovoCodex Biopharmaceuticals Co., Ltd.   **Control Drug - Lapatinib**   - **Name:** Lapatinib mesylate tablet - **Strength**: 250 mg - **Dosage form:** Tablet - **Dosage and administration**: The recommended dose is 1,250 mg, taken orally once a day, every 21 days as a cycle. It is recommended to take the daily dose in one lump, not in separate doses. It should be taken at least 1 hour before meal or at least 1 hour after meal. In combination with lapatinib, the dose of capecitabine is 2,000 mg/m^2^/d, taken orally in 2 doses, with an interval of about 12 hours, administered for 14 consecutive days and rest for 7 days, with 21 days as a cycle. It should be taken with food or within 30 minutes after a meal. - **Method of storage:** See the package insert for details - **Provider**: NovoCodex Biopharmaceuticals Co., Ltd.   **Control Drug - Capecitabine**   - **Name:** Capecitabine tablet - **Strength**: 0.5 g - **Dosage form:** Tablet - **Dosage and administration**: See the dosage and administration of lapatinib - **Method of storage:** See the package insert for details - **Provider**: NovoCodex Biopharmaceuticals Co., Ltd. |
| **Evaluation Indicators** | **Efficacy Evaluation Indicators**  All participants will be evaluated for anti-tumor efficacy every six weeks after randomization.  Imaging of primary lesions and/or metastatic lesions will be performed at each evaluation, and anti-tumor efficacy will be evaluated as per RECIST1.1.  **Primary Efficacy Evaluation Indicators**   - Progression-free survival (PFS) based on the Independent Review Committee (IRC) assessment, defined as the time from randomization to IRC-assessed disease progression (PD) or death due to any reason, whichever occurs first.   **Secondary Efficacy Evaluation Indicators**   - Overall survival (OS), defined as the time from randomization to death due to any reason; - Progression-free survival (PFS) based on the Investigator assessment, defined as the time from randomization to Investigator-assessed disease progression (PD) or death due to any reason, whichever occurs first; - Objective response rate (ORR, including PR and CR), defined as the proportion of patients with complete response or partial response. Anti-tumor efficacy will be evaluated as per RECIST1.1. For patients with PR or CR for the first time, efficacy must be confirmed after at least 4 weeks. Confirmation of efficacy can be carried out at planned tumor efficacy assessment every 6 weeks, as described in Appendix 3; - Disease control rate (DCR), defined as the proportion of patients with response (PR+CR) and stable disease (SD). For patients evaluated with SD, the efficacy needs to be evaluated as SD at least once after at least 12 weeks after randomization, as described in Appendix 3; - Duration of response (DOR), defined as the time to disease progression or death after the first evaluation as CR or PR.   **Safety Evaluation Indicators**  During the trial, participants need to undergo safety tests at the specified time, including laboratory tests (blood routine, urinalysis, blood biochemistry, etc.), vital signs, 12-lead electrocardiogram (ECG), and physical examination. The clinical manifestation, severity, occurrence time, end time, duration, treatment measures and outcome of any adverse event should be recorded, and its correlation with the investigational product should be determined. AEs are graded as per the NCI CTCAE V5.0 criteria, except for cardiac failure (graded as per the NYHA criteria). The correlation between any adverse event and the investigational product will be assessed by the Investigator as per the attribution evaluation criteria specified in the Protocol.  **Population Pharmacokinetic Evaluation Indicators**  PK data will be summarized using descriptive statistics (sample size, mean, geometric mean, standard deviation, minimum, median, maximum, coefficient of variation), if applicable. An appropriate model will be selected for PK analysis, if applicable, and the specific method is detailed in the independent statistical analysis plan.  **Immunogenicity Evaluation Indicators**  Anti-drug antibody (ADA) positive rate, and neutralizing antibody (NAb) positive rate. |
| **Statistical Hypotheses and Sample Size Calculation** | This trial intends to enroll 440 participants with HER2-positive locally advanced, recurrent or metastatic breast cancer, and assign the participants randomly to either the investigational product group or the control drug group in a 1:1 ratio, using the number of prior chemotherapy lines (0-1 vs. >1) received for recurrent or metastatic lesions and visceral metastases (yes vs. no) as stratification factors.  In the key phase III clinical trial of T-DM1, the median PFS of patients receiving lapatinib combined with capecitabine in the control group was 6.4 months; in the pivotal phase II clinical trial of pyrotinib maleate, the median PFS of patients receiving lapatinib combined with capecitabine in the control group was 5.6 months.  In combination with the above data, the efficacy of the study group will be compared with that of the control drug group using the IRC-assessed PFS as the primary endpoint in this trial. Assuming that the median PFS of the control drug group is 6.4 months, HR = 0.7, and the two-sided test level is 0.05, two interim analyses will be performed when 160 participants complete the Cycle 4 visit and then when the number of PFS events reaches 2/3 (see the Interim Analysis section for details) using Lan-DeMets α-spending function to approximate O'Brien-Fleming. Under these hypotheses, 335 IRC-assessed PFS events are required to gain a statistical power of 90%. Assuming 24 months of enrollment and an annual dropout rate of 5%, if we want to reach the target number of events 8 months after the last participant is enrolled, we approximately need a sample size of 220 participants/group, with a total of 440 participants in the trial. |
| **Interim Analysis** | In this trial, the primary analysis of PFS will be conducted when 335 PFS events assessed by the Independent Review Committee (IRC) occur. Two interim analyses will be planned during the trial. The interim analysis will be performed by the Independent Data Monitoring Committee (IDMC). Details will be specified in the IDMC Charter.   - The first interim analysis will be performed at the completion of the Cycle 4 visit in 160 participants (approximately 80 in each group), and the difference in point estimate of ORR between the two groups at that time will be calculated. If this difference is less than 2% during the interim analysis, a futility judgment should be considered to decide whether to continue the enrollment. Whether to continue the enrollment will be decided by the IDMC in combination with other safety and efficacy data at that time. Considering that in the key phase III clinical trial of T-DM1, the difference in the best ORR between the two groups was 12.7% (95% two-sided confidence interval (6.0%, 19.4%)), if the difference between the two groups in this trial is similar to that in the key phase III clinical trial of T-DM1, there is approximately a 10% probability that the two-group difference will be less than 2%. No test of superiority will be performed in this interim analysis, but for conservative reasons, Lan-DeMets α-spending function to approximate O'Brien-Fleming will be used. Conservatively, considering that the correlation coefficient between ORR and PFS will be 1 then (the actual correlation coefficient should be far less than 1), the efficacy boundaries will be calculated based on the actual number of PFS events. Under the same hypotheses as the calculation of sample size, the first interim analysis is expected at 12 months. The number of events is approximately 74 (22%). In this case, the efficacy boundary will be two-sided α=0.0002 at the first interim analysis. - The second interim analysis will be performed when 224 (2/3) IRC-assessed PFS events occur, and the superiority test will be performed and the sample size will be re-estimated. Using Lan-DeMets α-spending function to approximate O'Brien-Fleming, the efficacy boundary will be about two-sided α=0.0123 at the second interim analysis. The conditional power will be calculated based on the hazard ratio (HR) trend at that time. When the conditional power is not high enough (specific boundaries are specified in the IDMC Charter, roughly 60%-80%), the sample size will be re-estimated. - If the sample size is re-estimated at the second interim analysis, the hypothesis test statistics for the final analysis will be adjusted according to the method of Wassmer (2006) and Cui, Hung, Wang (1999). As per Lan-DeMets α-spending function to approximate O'Brien-Fleming, the efficacy boundary will be about two-sided α=0.0462 at the final analysis. If the final number of events or the number of events in the first and second interim analyses is slightly different from the plan (possibly due to the fact that multiple events occur on the same day when the number of events is reached), the efficacy boundaries will be adjusted accordingly based on the actual number of events. |
| **Statistical Methods** | Statistical analysis will be performed using SAS 9.4 or later. Continuous variables will be summarized using descriptive statistics, including number of participants, mean, median, standard deviation, maximum and minimum values. Categorical variable will be described by the number and percentage of participants in each category. Unless otherwise specified, the hypothesis test will use a two-sided test at α = 0.05.  **Statistical Analysis Population**   - **Intent-to-Treat (ITT) Analysis Set:** All randomized participants will be included in the ITT analysis set. The ITT analysis set will be used for the analysis of participant disposition, demographic and baseline characteristics, and will also be used as the primary analysis set for all efficacy endpoints. - **Per-Protocol Set (PPS):** All participants who are randomized and treated with at least one dose of investigational product or control drug without major protocol violations constitute the per-protocol set for this trial. The PPS will be used for the supporting analysis of the primary efficacy endpoints. - **Safety Analysis Set (SS):** All participants who are randomized and treated with at least one dose of investigational product or control drug and perform at least one post-treatment safety evaluation are included in the SS. The SS is the safety evaluation population of this trial. - **Population PK Analysis Set**: All participants who receive at least one dose of the investigational product treatment and have at least one post-dose PK data are included in the population PK analysis set. - **Immunogenicity Analysis Set:** All participants who receive at least one dose of the investigational product treatment and have at least one post-dose immunogenicity evaluation data are included in the immunogenicity analysis set.   **Statistical Analysis Methods**  **Demographic Data and Other Baseline Characteristics**  Demographic data and other baseline characteristics will be tabulated, summarized and descriptively analyzed using descriptive statistics.  **Efficacy Analysis**  The primary endpoint of this trial is the IRC-assessed PFS, and the primary analysis will be performed in the ITT population. The primary hypothesis test will first be performed on the difference in the IRC-assessed PFS between the treatment groups. If the IRC-assessed PFS is statistically different between the treatment groups, a hypothesis test will be further performed on the OS of the treatment groups at a two-tailed level of 0.05.  All time-to-event endpoints (such as PFS, DOR, OS, etc.) will be summarized and estimated using the Kaplan-Meier method. Survival functions of the two treatment groups will be compared using the Log-rank test stratified by randomization stratification factors. A Cox proportional risk model will also be used to calculate the hazard ratio (HR) between the two treatment groups and their corresponding confidence intervals using the treatment group and randomization stratification factors (the number of prior chemotherapy lines (0-1, >1) received for recurrent or metastatic lesions and whether they have concomitant visceral metastases (yes, no)) as independent variables.  The ORR of the investigational product group and the control drug group and its 95% confidence interval (estimated by the Clopper-Pearson method) will be calculated, respectively. The difference in ORR between the treatment groups and its 95% confidence interval will be calculated. The stratified Cochran-Mantel-Haenszel (CMH) method adjusted by randomization stratification factors will be used to calculate the P values of the treatment groups. DCR will be analyzed using a statistical method similar to that for ORR.  **Safety Analysis**  Adverse events will be coded using the Medical Dictionary for Regulatory Activities (MedDRA). They will be analyzed based on treatment emergent adverse events (TEAEs). TEAE is defined as an adverse event that newly occurs or worsens (of the adverse event that occurs before medication or the pre-existing medical conditions) during the period from the start of use of the investigational products to the last visit. TEAEs, TEAEs related to the investigational products, and serious adverse events (SAEs) in both groups will be summarized and analyzed by system organ class (SOC) and preferred term (PT), and the number of participants and incidence rates will be calculated.  Descriptive statistics will be used to summarize vital signs, physical examination, ECG and laboratory tests as well as their changes from baseline. Changes in laboratory tests relative to baseline will be described using a shift table.  **Population Pharmacokinetic Analysis**  PK data will be summarized using descriptive statistics (sample size, mean, geometric mean, standard deviation, minimum, median, maximum, coefficient of variation), if applicable. An appropriate model will be selected for PK analysis, if applicable, and the specific method is detailed in the independent statistical analysis plan.  **Immunogenicity Analysis**  The incidence of ADA will be summarized by visit. For ADA-positive participants, descriptive analysis will also be performed on the Nabs test results. If applicable, the effect of ADAs on the efficacy and safety of ARX788 will be evaluated. |
| **Trial Progress** | Estimated to be from January 2020 to August 2023 |

# Body of the Trial Protocol

### Study Background

#### Disease Background

Breast cancer is one of the most predominant malignant tumors in China, and is the most prevalent tumor in women. Epidemiological statistics^[1]^ showed that in 2011, the number of female breast cancer cases in China was about 249,000, with an incidence rate of 37.86/100,000, and the incidence had been on the rise in the last decade. The number of annual deaths was about 60,000, with a mortality rate of 9.21/100,000, and breast cancer mortality had been on the rise in the last decade. The 1-year, 3-year and 5-year observed survival rates in breast cancer patients were 90.5%, 80.0% and 72.7%, respectively, and the 5-year relative survival rate was 73.0% (95% CI was 0.712-0.749). The 2015 Chinese epidemiological prediction data for cancer^[2]^ suggested that breast cancer would remain the most prevalent tumor in women. In 2015, there were about 272,000 new cases of breast cancer and about 70,000 deaths in China. About 3% to 10% of new breast cancer cases each year have distant metastases at the time of diagnosis. Among early-stage patients, 30%-40% may develop into advanced breast cancer, with a 5-year survival rate of only 20% and an overall median survival time of 2-3 years^[3]^.

##### Diagnosis and Staging of Breast Cancer

The Guidelines for the Diagnosis and Treatment of Breast Cancer (2018.V1)^[4]^ published by the Chinese Society of Clinical Oncology (CSCO) in 2018 pointed out that the diagnostic examinations for breast cancer patients mainly include confirmatory diagnosis examination, pathological examination, and molecular typing examination. To be specific, the confirmatory diagnosis examination is performed to confirm the diagnosis and staging of breast cancer through evaluation of different sites such as the primary tumor, regional lymph nodes, and distant lesions, and the Guidelines recommend that clinical staging of breast cancer should refer to the 8th edition AJCC Cancer Staging Manual prepared by the American Joint Committee on Cancer. The pathological examination of tumor size, histological staging, histological grading, and presence of vascular invasion can guide the treatment of tumor patients and affect their prognosis. The molecular typing examination includes human epidermal growth factor receptor 2 (HER2), estrogen receptor (ER), progesterone receptor (PR) and Ki-67 genes, which are important for treatment selection of participants.

##### HER2 Expression and Testing in Breast Cancer

HER2, also known as c-erB2, is located on chromosome 17q12 and is a proto-oncogene. HER2 protein is a transmembrane protein with tyrosine protein kinase activity and is a member of the human epidermal growth factor receptor (EGFR) family. It is composed of three parts, namely, a extracellular ligand-binding domain, a single-chain transmembrane domain and an intracellular protein tyrosine kinase domain. HER2 protein binds to its ligand mainly by forming heterodimers with other members in the family. When binding to the ligand, it activates the activity of the tyrosine kinase mainly through receptor dimerization and autophosphorylation in the intracytoplasmic tyrosine kinase domain. The HER2 protein-mediated signal transduction pathways mainly include the Ras/Raf/mitogen-activated protein kinase (MAPK) pathway, the phosphatidylinositol 3 hydroxy kinase (PI3K)/Akt pathway, the signal transduction and activator of transcription (STAT) pathway, and the PLC pathway, which ultimately affect cell proliferation, survival, exercise and adhesion^[5]^.

King et al.^[6]^ reported that DNA from human breast cancer has amplification of this gene, and a study^[7]^ showed that HER2 is amplified in approximately 15%-20% of breast cancers, and that HER2 amplification is a major pathway of HER2 receptor overexpression and is a major driver of tumor development and progression in some breast cancers. HER2 receptor overexpression is an important adverse prognostic factor for breast cancer and a valuable therapeutic target. China’s Guidelines for the Diagnosis and Treatment of Breast Cancer (2018.V1) pointed out that with the increasing importance of driver genes, the clear determination of HER2 status has become an important principle for analytical and typing diagnosis. All invasive breast cancers should be tested for HER2 status.

Currently, the most commonly used methods for HER2 expression detection are immunohistochemistry (IHC) and in situ hybridization (ISH). It is recommended in the Guidelines for the Diagnosis and Treatment of Breast Cancer (2018.V1) that HER2 testing should follow the Chinese Guidelines for HER2 Testing of Breast Cancer (Version 2014)^[8]^ and the Expert Consensus on the Clinical Diagnosis and Treatment of Human Epidermal Growth Factor Receptor 2 Positive Breast Cancer 2016^[9]^. Currently, the Chinese guidelines for the HER2 testing of breast cancer have been updated based on the Guidelines for HER2 Testing of Breast Cancer (Version 2014), and are revised to the Guidelines for HER2 Testing of Breast Cancer (Version 2019)^[10]^.

##### Current Status of Breast Cancer Treatment

Traditional treatments for breast cancer include surgical treatment, radiotherapy, endocrine therapy, chemotherapy and targeted drug therapy. With the development of medical technology, the treatment of breast cancer has gradually evolved from local treatment to a combination of multiple treatments.

Surgery is the basic treatment for breast cancer, and the surgical method is determined by the size of the mass, the depth of infiltration and the lymph node metastasis. Radiotherapy is mainly used as an adjuvant therapy before or after radical breast cancer surgery, and as a palliative treatment for advanced breast cancer. Endocrine therapy is mainly used in patients with positive estrogen receptor (ER) and/or progesterone receptor (PR). Chemotherapy is an active treatment for all stages of breast cancer. Currently, the two main drugs in breast cancer chemotherapy are anthracyclines and taxanes, and other commonly used chemotherapy drugs include: vinorelbine, capecitabine, platinum, alkylating agents, etc. Targeted therapy is mainly targeted at patients with HER2-positive breast cancer. Currently, the main HER2-targeted drug recommended in the Guidelines for the Diagnosis and Treatment of Breast Cancer (2018.V1) is trastuzumab, which is widely used in patients with HER2-positive breast cancer at different stages of progression.

#### Current Status of HER2-Targeted Drug Therapy in Metastatic Breast Cancer

Currently, the advent of HER2-targeted therapy drugs has changed the HER2-positive breast cancer from an invasive disease with poor prognosis to a highly treatable disease with prolonged survival even in patients with metastatic disease. A better understanding of HER2 biology has enhanced the development of targeted therapies. The summary data^[11]^ showed that four HER2-targeted therapeutic drugs have been approved by the FDA for the treatment of HER2-positive metastatic breast cancer, i.e., trastuzumab, pertuzumab, T-DM1, and lapatinib, of which trastuzumab and lapatinib have also been approved for the treatment of metastatic breast cancer by the Chinese drug regulatory authority. Meanwhile, pyrotinib maleate, a double inhibitor of EGFR and HER2 tyrosine kinase that is independently developed in China, has been approved by the NMPA for production.

##### Trastuzumab

Trastuzumab (Herceptin^®^), developed by Roche, was approved by the FDA in 1998 for the treatment of patients with HER2 overexpressing metastatic breast cancer; it was approved by the former CFDA in 2002 as a single drug for the treatment of patients with metastatic breast cancer who have received one or more chemotherapy regimens or in combination with paclitaxel or docetaxel for the treatment of patients with metastatic breast cancer who have not received chemotherapy.

Clinical trials of trastuzumab in the treatment of metastatic breast cancer have explored its efficacy and safety in first-line, second-line and third-line treatment of HER2-positive metastatic breast cancer. The results in the package insert^[12]^ showed that in 2 efficacy validation studies, the trastuzumab combination treatment group had a significantly improved overall response rate (ORR) and median duration of response in participants compared with the chemotherapy treatment group.

In the H0648g study of the efficacy and safety of trastuzumab in first-line treatment of metastatic breast cancer, 496 patients with HER2-positive (IHC3+ or 2+) metastatic breast cancer who had not received chemotherapy were randomized to receive chemotherapy alone or trastuzumab in combination with chemotherapy. Trastuzumab was administered as a first loading dose of 4 mg/kg, followed by a weekly maintenance dose of 2 mg/kg by intravenous (IV) infusion. For patients who had received anthracyclines in the adjuvant treatment, chemotherapy was administered with paclitaxel (175 mg/m^2^, IV infusion for at least 3 hours, 21 days as 1 course, for at least 6 courses); for other patients, chemotherapy was administered with anthracyclines plus cyclophosphamide (AC: doxorubicin 60 mg/m^2^ or epirubicin 75 mg/m^2^ + cyclophosphamide 600 mg/m^2^, 21 days as 1 course, for 6 courses). In this trial, 65% of patients randomized to receive chemotherapy alone were treated with trastuzumab at the time of disease progression, as part of an independent extension study.

The study results showed that for participants in the trastuzumab combination group and the chemotherapy group, the median time to progression was 7.2 vs. 4.5 months, P < 0.0001; the objective response rate (ORR) was 45% vs. 29%, P < 0.001; the median time to response was 8.3 vs. 5.8 months; and the median survival was 25.1 (95% CI: 22,30) vs 20.3 (95% CI: 17,24) months.

##### Pertuzumab

Pertuzumab (Perjeta^®^) is another monoclonal antibody that targets HER2 and was approved by the FDA in 2012 to be used in combination with trastuzumab and docetaxel in patients with HER2-positive metastatic breast cancer who have not received anti-HER2 or chemotherapy drugs. The preferred regimen recommended in the National Comprehensive Cancer Network (NCCN) Guidelines for Breast Cancer (2019.V1) for the first-line treatment of patients with HER2-positive recurrent or metastatic breast cancer is trastuzumab combined with pertuzumab and docetaxel. According to the CSCO Guidelines for the Diagnosis and Treatment of Breast Cancer (2018.V1), the current international standard first-line treatment for HER2-positive advanced breast cancer is pertuzumab and trastuzumab dual-targeted combination with docetaxel. Pertuzumab has not been marketed in China, so currently the first-line preferred regimen in China is still trastuzumab combined with taxane drugs. However, patients are encouraged to enter the clinical trial of pertuzumab for better treatment. On December 17, 2018, the NMPA approved the use of pertuzumab in combination with trastuzumab and chemotherapy for the adjuvant treatment of patients with HER2-positive early breast cancer at a high risk of recurrence.

In the pivotal clinical trial^[13]^ of pertuzumab for first-line treatment of metastatic breast cancer (clinicaltrial.gov registration number NCT00567190), 808 patients with HER2-positive (IHC3+ or FISH+) were randomized to the pertuzumab combination treatment group or the placebo combination treatment group at a 1:1 ratio based on whether they had previously received (neoadjuvant) adjuvant therapy (yes, no) and their region (Asia, Europe, North America or South America) as stratification factors. In the study, trastuzumab was administered at an initial loading dose of 8 mg/kg, followed by a maintenance dose of 6 mg/kg every 3 weeks (Q3W); pertuzumab was administered at an initial loading dose of 840 mg, followed by a maintenance dose of 420 mg, Q3W; docetaxel was administered at an initial dose of 75 mg/m^2^, which could be increased to 100 mg/m^2^ later, Q3W, for at least 6 consecutive cycles if tolerated well by the participants.

The trial data showed that in the pertuzumab combination treatment group and the placebo combination treatment group, the median progression-free survival (PFS) was 18.5 vs 12.4 months (HR = 0.62 [95% CI: 0.51,0.75]), prolonged by 6.1 months; the OS was 56.5 vs 40.8 months (HR = 0.68 [95% CI: 0.56,0.84]), significantly prolonged by 15.7 months.

##### Ado-trastuzumab Emtansine (T-DM1)

T-DM1 is a targeted biologic combination of trastuzumab and a small molecule anti-cancer agent developed by Genentech. On February 22, 2013, Kadcyla^®^ (ado-trastuzumab emtansine, or T-DM1) was approved by the FDA for the treatment of HER2-positive advanced metastatic breast cancer that had not been responsive to trastuzumab and first-line taxane chemotherapy. T-DM1 connects trastuzumab to a drug called DM1 that interferes with the growth of cancer cells. T-DM1 delivers the drug to the cancer site to shrink the tumor, slowing down disease progression and prolonging survival. Currently, T-DM1 has not been approved for marketing in China. However, the CSCO Guidelines for the Diagnosis and Treatment of Breast Cancer (2018.V1) encourages previously treated patients with HER2-positive advanced breast cancer to participate in the clinical trials of this class of drugs.

In a randomized, open-label, multicenter clinical trial (EMILIA) (clinicaltrial.gov registration number NCT00829166)^[14]^ to evaluate the efficacy and safety of T-DM1 in patients with metastatic breast cancer, 991 patients with HER2-positive (IHC3+ or FISH+) unresectable locally advanced or metastatic breast cancer participated in the trial, and the enrolled participants had previously received treatment with taxane and trastuzumab. The participants were randomized to the T-DM1 treatment group or the lapatinib plus capecitabine treatment group at a ratio of 1:1 based on the number of prior chemotherapy lines (0-1, >1), whether there was visceral metastasis (yes, no) and region (USA, Western Europe, others) as stratification factors. In the trial, T-DM1 was administered at 3.6 mg/kg, Q3W, by IV infusion; lapatinib was administered orally at 1,250 mg, QD; and capecitabine was administered orally at 1,000 mg/m^2^ twice daily on Days 1-14 of the 21-day cycle, with all participants receiving long-term treatment with the investigational product.

Patients in the Kadcyla treatment group had an IRC-assessed mPFS of 9.6 months, compared with 6.4 months in the lapatinib plus capecitabine treatment group. Patients in the Kadcyla treatment group had an IRC-assessed mOS of 30.9 months, compared with 25.1 months in the lapatinib plus capecitabine treatment group.

##### Lapatinib

Lapatinib is a new oral breast cancer targeted therapy drug developed by GlaxoSmithKline, UK. It is a tyrosine kinase inhibitor that can effectively inhibit the activity of HER1 and HER2 tyrosine kinases. It is unique in that it can work in multiple ways to inhibit or kill tumor cells by preventing breast cancer cells from receiving the signals they need to grow. In 2007, TYKERB^®^ (lapatinib) was approved by the FDA in combination with capecitabine for the treatment of HER2 overexpressing, advanced or metastatic breast cancer previously treated with anthracycline, paclitaxel or trastuzumab (combination therapy can be used only after disease progression with trastuzumab); and in combination with letrozole in the treatment of patients with hormone receptor-positive and HER2 overexpressing metastatic breast cancer who have received hormone therapy after menopause. The CSCO Guidelines for the Diagnosis and Treatment of Breast Cancer (2018.V1) recommends that treatment regimens for patients to consider after progression with trastuzumab include lapatinib in combination with capecitabine, and that patients who cannot tolerate chemotherapy may select lapatinib monotherapy in combination with trastuzumab.

In a key clinical trial^[15]^ to evaluate the safety and efficacy of lapatinib combined with capecitabine in patients with HER2 overexpressing (IHC3+ or IHC2+ and FISH+) advanced breast cancer, a total of 399 patients with locally advanced or metastatic breast cancer who progressed after receiving treatment with anthracyclines, taxanes and trastuzumab were enrolled. The enrolled participants were randomized to the lapatinib plus capecitabine treatment group or the capecitabine treatment group at a ratio of 1:1 to receive lapatinib at 1,250 mg, QD and capecitabine at 2,000 mg/m^2^/day, administered on Days 1-14 per cycle (21 days), or capecitabine alone at 2,500 mg/m^2^/day, administered on Days 1-14 per cycle (21 days).

The trial results showed that the median time to progression (TTP) of the lapatinib plus capecitabine treatment group and the capecitabine group was 27.1 vs 18.6 weeks (HR = 0.57 [95% CI: 0.43-0.77], P = 0.00013).

##### Pyrotinib Maleate

Pyrotinib maleate (Airuini^®^) is an innovative drug independently developed by Jiangsu Hengrui Pharmaceuticals Co., Ltd. It is another oral targeted therapy drug for breast cancer after lapatinib. It is a tyrosine kinase inhibitor (TKI) of pan-ErbB receptor and a dual inhibitor of epidermal growth factor receptor (EGFR) and HER2 tyrosine kinase. It can achieve anti-cancer effect by blocking the signal transduction pathways of the epidermal growth factor receptor (HER) family (EGFR, HER2, HER3 and HER4). On August 13, 2018, pyrotinib was officially approved by the NMPA for production and treatment of patients with HER2-positive recurrent or metastatic breast cancer in combination with capecitabine who were previously untreated or previously treated with trastuzumab and were required to have previously received anthracycline or taxane chemotherapy.

This indication was conditionally approved based on the results of a phase II clinical trial^[16–17]^ that included 128 patients with recurrent or metastatic breast cancer who had previously received or had not received trastuzumab treatment. The full approval of this indication will depend on ongoing confirmatory trials to confirm the clinical benefits of pyrotinib maleate in this population. The trial enrolled 128 patients with HER2-positive breast cancer (IHC3+ or IHC2+ and FISH+); patient who had previously failed treatment with anthracyclines and taxanes (including adjuvant therapy and treatment for recurrent metastasis); and patients with recurrent or metastatic breast cancer who had received chemotherapy of no higher than the second line after recurrence/metastasis, and were randomized into two groups: one group used pyrotinib at 400 mg QD, combined with capecitabine at 1,000 mg/m^2^, BID (1 week off after 2 weeks of treatment); the other group used lapatinib at 1,250 mg QD, combined with capecitabine at 1,000 mg/m^2^, BID (1 week off after 2 weeks of treatment).

The IRC- and Investigator-assessed objective response rates of the pyrotinib + capecitabine treatment group and the lapatinib + capecitabine treatment group were (71.4% vs 49.2% [P = 0.0117]) and (78.5% vs 57.1% [P = 0.01]), respectively, and the IRC- and Investigator-assessed median progression-free survival (mPFS) were (12.6 vs 5.6 months [HR = 0.371]) and (18.7 vs 7.0 months [HR = 0.363]), respectively, with statistically significant differences in efficacy between the two treatment groups.

To confirm the efficacy and safety of pyrotinib combined with capecitabine in the treatment of HER2-positive recurrent or metastatic breast cancer, Jiangsu Hengrui Pharmaceuticals Co., Ltd. is conducting two randomized, controlled, phase III clinical trials of pyrotinib combined with capecitabine in the treatment of HER2-positive recurrent or metastatic breast cancer. One is a randomized, open-label, multicenter phase III clinical trial of pyrotinib in combination with capecitabine versus lapatinib in combination with capecitabine in the treatment of patients with HER2-positive recurrent or metastatic breast cancer who have previously used trastuzumab, and it plans to enroll 240 patients. The other is a randomized, double-blind, multicenter phase III clinical trial of pyrotinib in combination with capecitabine versus placebo in combination with capecitabine in the treatment of patients with HER2-positive recurrent or metastatic breast cancer who have previously used trastuzumab, and it plans to enroll 350 patients.

#### Information of Investigational Product

##### General Information of Investigational Product

ARX788 is an antibody-drug conjugate (ADC), consisting of two parts, an anti-HER2 monoclonal antibody and a toxin small molecule AS269. The anti-HER2 monoclonal antibody can specifically bind to human HER2, and AS269 is a highly effective microtubule inhibitor that inhibits cell growth. Using a proprietary technique of unnatural amino acids, AS269 is fixed-point coupled to the antibody as ARX788 by a modified amino acid (p-acetylphenylalanine, pAF) inserted at a specific position in the heavy chain of the antibody. This technique results in a highly consistent ARX788 with a drug to antibody ratio (DAR) of 1.8 to 2.0. The connection between the ARX788 antibody part and the small molecule toxin AS269 is very stable in plasma, and no free AS269 is expected to be released in the blood after administration. The biological activity of ARX788 begins when it binds to HER2 on the cell surface. ARX788 enters the cell via endocytosis and is hydrolyzed in lysosomes, releasing pAF-AS269 (modified amino acid-linked toxic molecule) which binds to microtubules to induce cell cycle arrest and death.

The excipients of ARX788 used in the clinical trial include histidine, histidine hydrochloride, trehalose and polysorbate 80.

##### Pharmacodynamic Studies

###### In Vitro Activity Study

The pharmacological activity of ARX788 has been demonstrated in many preclinical studies, including in vitro inhibition assays in a variety of human tumor cell lines (including breast, ovarian and gastric cancers) and in vivo assays in xenograft tumors. The in vitro half-maximal inhibitory concentrations (IC50) of ARX788 on various HER2-high expressing breast cancer cell lines were 17 pM (SKBR3), 125 pM (HCC1954) and 50 pM (BT-474); the IC50 on the HER2-high expressing gastric cancer cells was 17 pM (NCI-N87); the IC50 on the HER2-high expressing ovarian cancer cells was 46 pM (SKOV-3). However, AS269 and pAF-AS269 do not pass through the cell membrane easily, and they do not have in vitro toxicity even at 200-fold higher molar concentrations than ARX788.

ARX788 has sub-nM potency and efficacy in cells that express high level of HER2 receptors. ARX788 is not active in cell lines that express low levels of receptors. These studies have shown that the in vitro activity of ARX788 depends on certain HER2 receptor threshold values. When the activity of ARX788 and T-DM1 was evaluated in the same tumor cell lines, ARX788 had similar or better activity compared to T-DM1 and also showed better inhibition in trastuzumab-resistant cell lines. These in vitro activity comparison results show that ARX788 is expected to be used in some breast cancer patients who cannot be treated with trastuzumab and T-DM1.

###### In Vivo Activity Study

The study investigated the in vivo anti-cancer effect of ARX788 in mice xenograft tumor models using human tumor cell lines with breast cancer, ovarian cancer and gastric cancer, and evaluated it in comparison with T-DM1 in selected cell lines. The results showed that ARX788 exhibited strong anti-tumor activity in all high HER2 expression models, with tumor regression or complete regression after a single dose administration of 0.55-5 mg/kg; ARX788 showed significantly improved anti-cancer activity compared to other HER2-targeted ADC drugs.

ARX788 had no effect on the MDA-M-468 HER2-negative breast cancer cell transplantation tumor model. This is consistent with the in vitro experiment proving that ARX788 is not active on HER2-negative cells. The anti-tumor activity showed a dose-dependent pattern in all tumor models. ARX788 showed better or the same anti-tumor effect compared with T-DM1.

The HER2-targeting specificity and potent anti-cancer activity of ARX788 have served as effective support for exploring its anti-cancer activity in clinical trials.

##### Non-Clinical Pharmacokinetic Studies

###### Animal Pharmacokinetics

Pharmacokinetic (PK) studies of ARX788 were conducted in rats and cynomolgus monkeys, assaying ARX788, total antibody and metabolite pAF-AS269, or as a stand-alone treatment, with PK assessment after administration of pAF-AS269 in rats.

The PK characteristics of both ARX788 and the total antibody exhibited low serum clearance and long serum half-life (T_1/2_), which were similar to other monoclonal antibody drugs. Exposure after administration of ARX788 was near dose proportional in rats and showed near or greater than dose proportional in cynomolgus monkeys. Neither ARX788 nor total antibody showed sex differences in systemic drug exposure in rats and cynomolgus monkeys. The T_1/2_ of ARX788 ranged from 118 to 196 h at a dose of 20 mg/kg in the rat model and 99 to 170 h at a dose of 5 mg/kg in the cynomolgus monkey model. Systemic drug exposure to ARX788 and total antibody increased with dose and at a greater rate in rats than in cynomolgus monkeys. No significant accumulation of ARX788 drug was observed in cynomolgus monkeys after 4 doses (3 weeks apart).

The binding of the antibody part and the toxin small molecule part in ARX788 was stable, and the levels of ARX788 and total antibody in the blood circulation were similar during the toxicological studies in rats and cynomolgus monkeys. This indicates that after ARX788 is injected into the blood circulation, the toxin molecule remains bound to the antibody (no release is found at the detection level), and that the stability of this ADC payload limits the systemic exposure of small molecule toxin.

ARX788 binds to HER2 in the target cell and is metabolized after endocytosis into the cell, producing the sole metabolite pAF-AS269. pAF-AS269 is usually detectable in animals 3 days after ARX788 administration. After administration of ARX788 to rats and cynomolgus monkeys, pAF-AS269 slowly appeared in the blood circulation at low concentrations near the lowest limit of quantification (LLOQ = 0.050 ng/mL). Quantifiable pAF-AS269 was observed in the blood system only at doses of ≥ 10.0 mg/kg in rats and ≥ 5.0 mg/kg in cynomolgus monkeys.

In the rat model, pAF-AS269 was detected in individual rats up to 0.0800 ng/mL at 120 or 168 h after administration of ARX788 at a dose of 10 mg/kg. In the 20 mg/kg dose group, pAF-AS269 was detected in all rats (with varying numbers of detections per rat) up to 0.275 ng/mL 24 to 240 h after administration of ARX788. In the study of pAF-AS269 alone in rats, pAF-AS269 had low systemic drug exposure and was rapidly cleared, with T_1/2_ of 0.606-0.839 h.

In the cynomolgus monkey model, pAF-AS269 was not detected 72-168 h after single-dose administration of ARX788. At the highest non-severely toxic dose (HNSTD), the C_max_ and AUC_(0-480_ _h)_ of pAF-AS269 were 0.849 ng/mL and 283 h*ng/mL in male cynomolgus monkeys (20 mg/kg ARX788) and 0.593 ng/mL and 217 h*ng/mL in female cynomolgus monkeys (20 mg/kg ARX788). In the multi-dose toxicological studies of cynomolgus monkeys (every 3 weeks, 4 times in total), pAF-AS269 was equal to or slightly higher than LLOQ (0.200 ng/mL) in the system at the HNSTD dose (10 mg/kg). On Day 64, after the last dose, C_max_ and AUC_(all)_ of pAF-AS269 in females and males were 0.440 ng/mL, 53.6 h*ng/mL and 0.485 ng/mL, 62.8 h*ng/mL, respectively. In both single-dose and multi-dose monkey toxicity studies, low levels of pAF-AS269 were detected in the blood circulation, with exposures less than 0.1% of ARX788 (molar concentration) at all dose levels.

###### Pharmacokinetics in Xenograft Tumor Models

The PK characteristics of ARX788 and its metabolite pAF-AS269 were studied in HER2-positive HCC1954 human breast and NCI-N87 human gastric transplantation tumor models. Nine female BALB/c nude mice were administered a single dose of 1.67 mg/kg (HCC1954 model) and 5 mg/kg (NCI-N87 model) of ARX788 intravenously, and the AUC_(0-last)_ of ARX788 was: 1,980,051 h*ng/mL for HCC1954 and 6,078,384 h*ng/mL for NCI-N87. The concentrations of pAF-AS269 detected at all time points were below the lower limit of quantification (LLOQ).

ARX788 had significantly lower total clearance and a smaller steady-state apparent volume of distribution (Vd_ss_) with mean serum T1/2 of 152 h and 160 h. The AUC was dose proportional in both trials.

##### Toxicologic Study

###### Single-Dose Toxicologic Study

**Single-Dose Toxicologic Study in Rats**

A single intravenous dose of 0.3, 0.9 or 1.35 mg/kg of pAF-AS269 was administered to rats over approximately 20 minutes and was well tolerated with no test-substance-related changes (clinical signs, weight changes, food intake, serum blood biochemistry, coagulation, blood routine or urinalysis); and no pAF-AS269-related terminal weight, organ weights, microscopic examination or histopathological changes. The maximum tolerance of pAF-AS269 was > 1.35 mg/kg in both male and female rats under the present test conditions.

No unplanned deaths were observed in rats given ARX788 at doses of 20, 60, or 90 mg/kg in a single intravenous drip over approximately 20 to 23 minutes. The clinical symptoms related to the investigational product were seen in one female animal in the 90 mg/kg group, which specifically manifested as: body temperature decrease and hair contamination; non-harmful effects related to the investigational product at doses ≥ 60 mg/kg were a decrease in mean weight accompanied by a decrease in food intake, and harmful effects were changes in serum biochemical parameters that were related to pathological changes in the liver, kidneys and immune organs (including spleen, thymus and bone marrow). Hematologic changes at doses ≥ 60 mg/kg and urinary changes at doses ≥ 20 mg/kg were related to pathologic changes in the kidneys, liver, spleen, and thymus. The kidneys, lungs, epididymis and testes had pathological changes related to the investigational product at a dose of 20 mg/kg. Under the present test conditions, 90 mg/kg was considered the maximum tolerated dose (MTD) of ARX788, at which the C_max_ and AUC_(0-336_ _h)_ of ARX788 were 2,010,000 ng/mL and 101,000,000 h*ng/mL in male animals, and 1,720,000 ng/mL and 109,000,000 h*ng/mL in female animals.

**Single-dose Toxicologic Study in Cynomolgus Monkeys**

The potential toxicity of ARX788 was evaluated by autopsy of cynomolgus monkeys (2/sex/group) administered at a single dose of 10, 20 or 30 mg/kg via intravenous injection (injection time of about 20 minutes) on Day 22.

One female monkey in the 30 mg/kg group was euthanized on Day 15 after near death. This female monkey had significant weight loss, increased white blood cells and reticulocytes, decreased platelets (with associated increased mean platelet volume) and decreased circulating red blood cell volume (increased width of red blood cell distribution). The cause of animal death was confirmed by microscopic observation of the liver, kidneys, thymus, spleen, duodenum and maxillary glands.

In all dose groups, manifestations related to ARX788 were observed in animals that survived until the final autopsy, including: severe weight loss, clinicopathologic changes, and microscopic findings in the liver (hepatocyte hypertrophy, necrosis, and increased mitosis), kidneys (glomerular lesions), immune organs (including lymphatic depletion of spleen, thymus and sternal bone marrow, increased mitosis, increased macrophages and/or necrosis of splenic red marrow monocytes; marrow hypercellular in sternal bone marrow and/or degenerative/necrotic bone marrow of megakaryocytes), duodenum (shortened villi, increased mitosis in the crypt, and degeneration/necrosis in the duodenal glands), and lungs (multifocal crimson changes in the right and caudal lobes, mild diffuse alveolar septal thickening).

The clinicopathologic changes were generally consistent with the microscopic findings, including increased ALT, AST, bilirubin total, alkaline phosphatase, GGT, cholesterol, triglyceride, globulin, white blood cells, reticular cells, neutrophils, monocytes, activated partial thromboplastin time and fibrinogen, as well as decreased platelets, albumin and A/G ratios. In general, the severity and frequency of clinicopathological changes increased with dose.

The HNSTD in male monkeys was confirmed at 30 mg/kg, at which the C_max_ and AUC_(0-480_ _h)_ of ARX788 in male monkeys were 722,000 ng/mL and 108,000,000 h*ng/mL, and the C_max_ and AUC_(0-480_ _h)_ of pAF-AS269 were 0.849 ng/mL and 283 h*ng/mL. The HNSTD in female monkeys was confirmed at 20 mg/kg, at which the C_max_ and AUC_(0-480_ _h)_ of ARX788 in female monkeys were 574,000 ng/mL and 57,800,000 h*ng/mL, and the C_max_ and AUC_(0-480_ _h)_ of pAF-AS269 were 0.593 ng/mL and 217 h*ng/mL.

###### Multi-Dose Toxicologic Study

Multiple-dose toxicologic studies of ARX788 in cynomolgus monkeys were conducted at 3.3, 10 or 15 mg/kg every 3 weeks with 4 repeated administrations. After the second administration, three (out of seven) cynomolgus monkeys in the 15 mg/kg dose group died related to the investigational product. The 15 mg/kg dose group discontinued administration after the second administration. As observed in single-dose administration, the changes related to the investigational product included weight loss, serum chemistry and hematological changes.

All monkeys in the 3.3 and 10 mg/kg groups were not found to have any lesions or deaths related to the investigational product during the four doses and recovery. No changes in cardiovascular, ocular, respiratory or neurological functions related to the investigational product were found in all dose groups. Changes in blood and coagulation parameters were dose-dependent. Increased white blood cells, neutrophils, basophils, monocyte count, red blood cell distribution width (RDW) and absolute reticulocyte count, as well as decreased hemoglobin were observed in the 3.3, 10 and/or 15 mg/kg dose groups. Except for the increase in RDW, all the above changes recovered after the end of the recovery period.

In the 3.3, 10 and/or 15 mg/kg dose groups, there were transient, mild to severe changes, including increased AST, GGT, cholesterol total and triglycerides, as well as decreased protein total, A/G ratio and phosphorus. These changes are consistent with the microscopic findings in the kidneys and liver. No changes were detected at the end of the recovery period, except in the 10 and/or 15 mg/kg dose groups, where there were increased triglycerides, decreased protein total, and decreased A/G ratio. These changes indicate the presence of toxic side effects at doses of 10 and/or 15 mg/kg.

Histopathological results related to the investigational product in the 3.3 mg/kg dose group showed that there were no toxic side effects at the 3.3 mg/kg dose. The no observed adverse effect level (NOAEL) was determined to be 3.3 mg/kg for male and female monkeys (C_max_ and AUC_(0_-_480_ _h_) of ARX788: 81,400 ng/mL and 1,460,000 h*ng/mL for male monkeys and 83,200 ng/mL and 1,420,000 h*ng/mL for female monkeys).

Test-related changes were found in the kidneys and rectum of animals given 4 doses of 10 mg/kg ARX788. These changes were considered adverse because of their potential harmful effects on the organ function and/or overall health of the animals. The renal findings included weight gain, and an increase in glomerular mesangial matrix, tubular dilatation, and albumin casts. The weight gain of the kidney was reversible in males but not in females; the microscopic changes persisted during the recovery period and progressed to glomerulosclerosis and interstitial fibrosis. Changes in the male rectum included small ulcers and submucosal neutrophilic inflammation, which were reversible. All other observations were not considered adverse because of the minimal or mild impact on the overall health of the animals or the lack of probability.

Very mild alveolar septal thickening was found in animals in the 10 or 15 mg/kg dose group and was associated with an increase in lung weight. One male animal in the 10 mg/kg dose group developed fibrosis after alveolar septal thickening. This change was also observed in three animals experiencing early death in the 15 mg/kg dose group, which were characterized by: increased eosinophils and increased monocytes in the alveolar septum; animals in the ≥ 3.3 mg/kg dose group experienced minimal to mild increases in alveolar macrophages, which were considered non-negative because the severity was very low.

Adverse findings in the 15 mg/kg dose group included early death and changes in the kidneys and gastrointestinal tract. Increased glomerular mesangial matrix, tubular dilatation, vacuolar formation and mixed cell infiltration were found in the kidneys of animals that died in the early stage. Findings in the gastrointestinal tract included mixed cellular infiltration in the mucosa, submucosa, and/or stomach serosa, duodenum, and/or colon; submucosal edema of the stomach and duodenum; hemorrhage in the mucosa, submucosa, and/or serosa of the stomach, duodenum, colon, and/or rectum; dilated crypt/gland of the duodenum; and neutrophilic inflammation of the mucosa/submucosa of the colon. In surviving animals, during the next 8-week recovery period, the renal weight increased, with a corresponding increase in glomerular mesangial matrix; the additional microscopic changes included tubular dilation, albumin casts, renal interstitial fibrosis, rectal mucosal ulceration with neutrophilic inflammation and submucosal edema. In the next 14-week recovery period, microscopic changes in the kidneys (increased glomerular mesangial matrix, tubular dilatation, albumin casts, and interstitial fibrosis) still existed. All other microscopic changes have recovered.

A dose of ≥ 10 mg/kg was considered to be adverse because it caused many serious reactions such as weight loss, decreased activity, arched back posture, inappetence and changes in serum chemical parameters, as well as histopathological findings in the kidneys and gastrointestinal tract.

Weight loss in males continued through the recovery stage at all dose levels, but no significant weight change was observed in females at any dose level by the end of the recovery stage. In the 10 mg/kg dose group, pathological findings in the kidneys and rectum related to the investigational product were considered adverse because they had potential harmful effects on the function of the related organs and/or the overall health of the animals. All other findings were not considered adverse because of their small or low severity and/or lack of probability of effect on the overall health of the animals.

The changes in the blood recovered at the end of the recovery period, except for the increase in RDW and fibrinogen. These changes, although considered to be related to the investigational product, were not considered to be adverse due to the short duration of the changes, small effects and/or reversibility. For the 10 and/or 15 mg/kg dose groups, changes in the AST, GGT, cholesterol total, triglycerides, protein total, A/G, urea and creatinine were consistent with the microscopic observations in the kidneys and liver. These clinical chemistry changes recovered after the end of the recovery period, except for the increased cholesterol total and triglycerides and the decreased A/G.

Most pathological and clinical observations were reversible for ARX788 at a dose of 10 mg/kg, and all animals tolerated 4 doses during administration and recovery. The HNSTD was determined to be 10mg/kg for males and females (C_max_ and AUC_(0-480 h)_ of ARX788: 223,000 ng/mL and 4,100,000 h*ng/mL for males and 244,000 ng/mL and 4,310,000 h*ng/mL for females).

###### Genetic Toxicology Studies

The potential mutagenicity of pAF-AS269 was determined by measuring its ability to induce reverse mutagenesis at some loci in Salmonella typhimurium (TA98, TA100, TA1535 and TA1537) and the tryptophan locus in Escherichia coli WP2 uvrA in the presence or absence of an exogenous metabolic activator (polychlorinated biphenyls 1254-induced rat liver S9). pAF-AS269 was available in concentrations of 100, 250, 500, 1,000, 2,500 and 5,000 μg per plate. The bacterial reverse mutation test for pAF-AS269 was negative for all conditions tested.

The potential ability of pAF-AS269 to cause chromosome damage was determined by the chromosome mutation test. The test was performed using human lymphocytes and pAF-AS269 was used at concentrations of 10, 25, 50, 100 and 250 μg/mL in the presence or absence of polychlorinated biphenyls-1254-induced activation of rat liver S9 system. Under the conditions of the current study, pAF-AS269 was negative in the induction of chromosomal mutation in human lymphocytes.

In the rat bone marrow micronucleus test, the lysis-inducing activity of pAF-AS269, and/or its ability to disrupt mitosis were evaluated by measuring the frequency of micronucleus formation by polychromatic erythrocytes (PCEs) in the bone marrow of adult SD rats after single intravenous infusions of 4.5, 13.5 and 40 mg/kg pAF-AS269. A significant increase in micronucleus formation was observed in the 40 mg/kg dose group compared to the parallel blank control group at both 24 h and 48 h sampling points (P < 0.05, analysis of variance (ANOVA)). In addition, positive control samples significantly increased the induction of micronucleus formation compared to the parallel blank control group (P < 0.05, ANOVA). A statistical analysis of the 24-hour sampling points revealed a dose-related response for pAF-AS269 (P < 0.01 0.05, Cochran-Armitage). Based on the above data, pAF-AS269 was evaluated as positive for the rat bone marrow micronucleus test under the current study conditions.

##### Previous Clinical Trials

###### First-in-human Trial

This trial was a multicenter, open-label, dose-escalation phase Ia clinical trial of intravenous infusion of ARX788 alone for treatment of HER2-positive advanced cancer (NCT02512237). In this trial, HER2-positive was defined as fluorescence in situ gene hybridization (FISH) + immunohistochemistry (IHC) of 3+. The current pharmacokinetic, efficacy and safety data were mainly from 9 participants in 5 dose groups, which were: 0.33 mg/kg (1 participant), 0.66 mg/kg (1 participant), 1.3 mg/kg (3 participants), 2.20 mg/kg (3 participants) and 2.9 mg/kg (1 participant).

**Safety results:**

Nine participants were treated with the investigational product at doses of 0.33 to 2.9 mg/kg and completed safety observations; DLT developed after the first dose of 2.9 mg/kg and was Grade 3 mucosal inflammation. Non-infectious pneumonia was determined to be a significantly delayed pulmonary toxicity, which occurred after 4 to 5 cycles of administration in the ≥ 1.3 mg/kg dose groups: one participant in the 1.3 mg/kg group developed Grade 2 non-infectious pneumonia after 4 cycles of treatment, and 2 participants in the 2.2 mg/kg group developed Grade 2 and Grade 5 non-infectious pneumonia after 4 to 5 cycles of treatment. Other investigational product-related AEs included Grade 1 ocular toxicity (hazy vision, dry eye, itchy eye syndrome) and Grade 1-2 alopecia. No AEs related to liver, kidney, heart, thrombocytopenia and neutropenia were found.

In the trial, non-infectious pneumonia was determined to be a serious investigational product-related AE at doses of ≥ 1.3 mg/kg: No non-infectious pneumonia was observed in the 0.33 mg/kg group within 13 treatment cycles and in the 0.66 mg/kg group within 7 treatment cycles; 1 Grade 2 non-infectious pneumonia was observed in the 1.3 mg/kg group after 4 cycles of treatment, and improved after discontinuation and steroid therapy; 2 participants in the 2.2 mg/kg group experienced Grade 2 and Grade 5 non-infectious pneumonia after 4 cycles of treatment, the participant with Grade 2 event recovered with no sequelae while the participant with Grade 5 event died after discontinuation and steroid therapy. PK data showed a significant accumulation of ARX788 between Cycle 1 and Cycle 3 in the participant with Grade 5 non-infectious pneumonia, with an increase in AUC_(0-inf)_ from 8.66 x 10^6^ h*ng/mL in Cycle 1 to 17.6 x 10^6^ h*ng/mL in Cycle 3, the highest exposure among the 9 participants.

These data suggest that non-infectious pneumonia associated with ARX788 may be reversible and that the risk and severity of its occurrence may be dose and exposure related.

**Pharmacokinetic results:**

Preliminary PK results of ARX788, metabolite pAF-AS269, and total antibody were obtained for ARX788 at doses ranging from 0.33 to 2.9 mg/kg, based on a dosing regimen of intravenous infusion every 3 weeks (Q3W) (90 minutes; the duration of infusion may be reduced to 60 minutes after the first cycle if well tolerated and there is no infusion reaction, at the discretion of the Investigator). The PK parameters of ARX788 are shown in Table 1 below.

Table 1 Summary of pharmacokinetic parameters in the first-in-human trial of ARX788

| **Cycle** |  | **Dose**  **mg/kg** | **Number of participants**  **n** |  | **Statistics** | **AUC_(0-inf)_**  **h*ng/mL** | **AUC_(0-t)_**  **h*ng/mL** | **CL or CL_ss_**  **L/h/kg** | **C_max_**  **ng/mL** | **t_1/2_**  **h** | **t_max_**  **h** | **V_z_ or V_ss_**  **L/kg** |
| --- | --- | --- | --- | --- | --- | --- | --- | --- | --- | --- | --- | --- |
| C1 |  | 0.33 | 1 |  | Observed value | 658000 | 636000 | 0.000502 | 8440 | 122 | 2.00 | 0.0881 |
|  |  | 0.66 | 1 |  | Observed value | 2510000 | 2340000 | 0.000262 | 22700 | 131 | 2.00 | 0.0498 |
|  |  |  |  |  | Mean | 4860000 | 4230000 | 0.000359 | 38200 | 116 | 3.00 | 0.0469 |
|  |  | 1.30 | 3 |  | Minimum | 2370000 | 2180000 | 0.000156 | 35600 | 55.9 | 1.50 | 0.0291 |
|  |  |  |  |  | Maximum | 8530000 | 6830000 | 0.000561 | 40900 | 212 | 5.50 | 0.0639 |
|  |  |  |  |  | Mean | 7780000 | 6690000 | 0.000285 | 47200 | 176 | 2.00 | 0.0719 |
|  |  | 2.20 | 3 |  | Minimum | 6990000 | 6180000 | 0.000254 | 43100 | 158 | 2.00 | 0.0687 |
|  |  |  |  |  | Maximum | 8660000 | 7130000 | 0.000315 | 50600 | 205 | 2.00 | 0.0750 |
| C3 |  | 2.90 | 1 |  | Observed value | 13800000 | 9040000 | 0.000210 | 61400 | 225 | 2.00 | 0.0682 |
|  |  | 0.33 | 1 |  | Observed value | 732000 | 724000 | 0.000456 | 8780 | 68.4 | 2.00 | 0.0422 |
|  |  | 0.66 | 1 |  | Observed value | 2460000 | 2370000 | 0.000278 | 20700 | 98.8 | 2.00 | 0.0388 |
|  |  |  |  |  | Mean | 7830000 | 6610000 | 0.000282 | 47300 | 136 | 1.50 | 0.0421 |
|  |  | 1.30 | 2 |  | Minimum | 3080000 | 3060000 | 0.000131 | 41600 | 60.2 | 1.50 | 0.0383 |
|  |  |  |  |  | Maximum | 12600000 | 10200000 | 0.000434 | 53000 | 212 | 1.50 | 0.0459 |
|  |  |  |  |  | Mean | 13900000 | 10300000 | 0.000206 | 63700 | 217 | 1.83 | 0.0636 |
|  |  | 2.20 | 3 |  | Minimum | 12000000 | 7890000 | 0.000174 | 56300 | 177 | 1.50 | 0.0533 |
|  |  |  |  |  | Maximum | 17600000 | 12600000 | 0.000234 | 69300 | 260 | 2.00 | 0.0722 |

The exposure and C_max_ of ARX788 increased with dose. In Cycle 3, the highest observed AUC_(0-t)_ and C_max_ were 12,600,000 h*ng/mL and 69,300 ng/mL, respectively. The concentration range of ARX788 before administration in Cycle 3 was 124 ng/mL to 10,500 ng/mL. The system clearance ranged from 0.000131 to 0.000561 L/h/kg, and T_1/2_ was 55.9 to 260 hours. The half-life increased with dose. Both CL and T_1/2_ were within the predictive range derived from the data of rats and monkeys. For most participants in the dose groups of ≥ 1.3 mg/kg, accumulation of ARX788 was observed, with a maximum 1.8-fold increase in AUC_(0_-_t)_ and a maximum 1.6-fold increase in Cmax from Cycle 1 to Cycle 3.

Similar to ARX788, total antibody exposure increased with dose. In Cycle 3, the highest observed AUC_(0-t)_ and C_max_ were 7,020,000 h*ng/mL and 49,100 ng/mL, respectively. The T_1/2_ of total antibody elimination ranged from 17.1 to 166 hours. It is expected that the concentration of total antibody will be equal to or higher than ARX788. However, the observed result was contrary (ARX788 to total antibody C_max_ ratio was between 1.1 and 1.5 in Cycle 3). Additional tests are being performed to determine the effect of other interference factors on total antibody and ARX788 determination.

The metabolite of ARX788, pAF-AS269, was detected in three participants at 1.3, 2.2 and 2.9 mg/kg at levels close to the limit of quantification (0.2 to 0.5 ng/mL). Although data were limited, overall pAF-AS269 exposure may be correlated with dose and ARX788 exposure, as the participant with the highest ARX788 AUC_(0-t)_ had the highest pAF-AS269 AUC_(0-t)_.

**Efficacy results:**

All 7 evaluable participants in this trial had varying degrees of tumor reduction. The best tumor response observed was partial response (PR) in 1 participant each in the 0.66, 1.3, and 2.2 mg/kg groups (33.3% of the total sample size). Four participants (44.4% of the total sample size) in the 0.33, 1.3 and 2.2 mg/kg groups had stable disease over 3 to 13 courses of continuous treatment.

###### First-in-human Trial in China

This trial was a single-center, open-label, dose-escalation phase Ia clinical trial, with the primary objective to evaluate the safety, tolerability and pharmacokinetic profile of ARX788 monotherapy in HER2-positive advanced breast cancer, where HER2-positive was defined as IHC 3+ and/or FISH+. Dose escalation was performed in a “3+3” design, and the available data were obtained from 51 participants in 9 different dose groups that met the efficacy and safety assessment, namely, 0.33 mg/kg Q3W (3 participants); 0.66 mg/kg Q3W (3 participants); 0.88 mg/kg Q3W (3 participants); 1.1 mg/kg Q3W (8 participants); 1.3 mg/kg Q3W (12 participants); 1.5 mg/kg Q3W (11 participants); 0.88 mg/kg Q4W (4 participants); 1.1 mg/kg Q4W (3 participants); and 1.3 mg/kg Q4W (4 participants).

**Safety results:**

As of January 20, 2020, a total of 51 participants entered the safety analysis set. A total of 51 (100%) participants reported treatment-emergent adverse events (TEAEs), and 49 (96.1%) participants experienced TEAEs related to the investigational product, of which most of the participants (45 participants, 91.8%) had the highest severity of Grade 2, and 11 participants (22.4%) had the highest severity of Grade 1; a small number of participants (4 participants, 8.2%) had severity of Grade 3-4, of which 3 participants (6.1%) with Grade 3 and 1 participant (2.0%) with Grade 4.

The most common system organ classes of the TEAEs related to the study included: investigations (47 participants, 92.2%), eye disorders (26 participants, 51%), general disorders and administration site conditions (22 participants, 43.1%), metabolism and nutrition disorders (22 participants, 43.1%), gastrointestinal disorders (21 participants, 41.2%), respiratory, thoracic and mediastinal disorders (19 participants, 39.2%), and skin and subcutaneous tissue disorders (18 participants, 35.3%).

The most common preferred terms of the TEAEs related to the study included: aspartate aminotransferase increased (35 participants, 68.2%), Alanine aminotransferase increased (26 participants, 51.0%), hypokalemia (16 participants, 31.4%), asthenia (16 participants, 31.4%), alopecia (16 participants, 31.4%), dry eye (15 participants, 29.4%), adrenocorticotropic hormone abnormal (15 participants, 29.4%), neutrophils decreased (12 participants, 23.5%), plasma aldosterone increased (12 participants, 23.5%), and dry mouth (12 participants, 23.5%).

Six (12.5%) participants experienced treatment-emergent SAEs, which were shock, osteomyelitis and epistaxis, respectively in 1 participant, and 3 participants developed infectious pneumonia, among which only 1 participant (1.5 mg/kg Q3W group) experienced the treatment-emergent SAE related to the investigational product.

As of January 20, 2020, a total of 11 (21.6%) participants had ≥ Grade 3 TEAEs, including: infectious pneumonia that occurred in 3 participants, and osteomyelitis, weight increased, neutrophil count decreased, hyponatremia, back pain, epistaxis, shock and electrocardiogram QTc interval prolongation that occurred in one participant each. Grade 3 or higher events that the Investigator determined to be related to the investigational product included: Grade 3 hyponatremia in 1 participant, Grade 4 neutrophil count decreased in 1 participant, Grade 3 electrocardiogram QTc interval prolonged in 1 participant, and Grade 3 pneumonia in 1 participant.

Thirty-two (62.7%) participants experienced treatment-related adverse events of special interest (AESIs), including ocular toxicity in 27 participants (52.9%), hepatotoxicity in 8 participants (17.6%), pulmonary toxicity in 9 participants (17.6%), and hematological toxicity in 1 participant (2.0%).

No participants in this phase I trial experienced DLT.

**Pharmacokinetic results:**

Blood samples were collected after the first and third doses and before dosing in each treatment cycle for the PK test of ARX788 in participants with advanced breast cancer. Currently, the PK results were obtained for the 0.33 mg/kg Q3W, 0.66 mg/kg Q3W, 0.88 mg/kg Q3W, 1.10 mg/kg Q3W, 1.30 mg/kg Q3W, 0.88 mg/kg Q4W, 1.10 mg/kg Q4W, and 1.30 mg/kg Q4W dose groups. The PK parameters of the intact drug ADC in the participants treated at the Q3W dosing interval showed:

- - - 0.33 mg/kg Q3W (1 participant): T_1/2_ of the third dosing cycle was 356 h;
    - 0.66 mg/kg Q3W (2 participants): T_1/2_ of the third dosing cycle was 100 h;
    - 0.88 mg/kg Q3W (3 participants): T_1/2_ of the third dosing cycle was 76.0 h;
    - 1.1 mg/kg Q3W (8 participants): T_1/2_ of the third dosing cycle was 86.4 h;
    - 1.3 mg/kg Q3W (4 participants): T_1/2_ of the third dosing cycle was 130 h;
    - 1.5 mg/kg Q3W (3 participants): T_1/2_ of the third dosing cycle was 154 h;

Exposure to intact ADC, total antibody and metabolite pAF-AS269 in serum of participants administered Q3W increased with the dose of ADC (ARX788) in both Cycle 1 and Cycle 3. The analysis results of the accumulation factors of the main parameters are shown in Table 2 below.

Table 2 Analysis of accumulation factors of intact ADC pharmacokinetic parameters in human body of Chinese participants with advanced breast cancer treated with ARX788

| **DOSE** | **PT** | **C_max__C1**  **(ng/mL)** | **C_max__C3**  **(ng/mL)** | **AUC_(0-tau)__C1**  **(h*ng/mL)** | **AUC_(0-tau)__C3**  **(h*ng/mL)** |
| --- | --- | --- | --- | --- | --- |
| 0.33 mg/kg Q3W | N | 1 | 1 | 1 | 1 |
|  | Mean | 6840 | 6530 | 602134 | 457854 |
|  | SD | - | - | - | - |
| 0.66 mg/kg Q3W | N | 2 | 2 | 2 | 2 |
|  | Mean | 15750 | 14600 | 1626901 | 1371258 |
|  | SD | 354 | 2121 | 274533 | 141139 |
| 0.88 mg/kg Q3W | N | 3 | 3 | 3 | 3 |
|  | Mean | 19200 | 19867 | 2158157 | 2354409 |
|  | SD | 1572 | 833 | 412534 | 425836 |
| 1.1 mg/kg Q3W | N | 8 | 8 | 8 | 8 |
|  | Mean | 24113 | 24763 | 2462840 | 2905757 |
|  | SD | 2387 | 3174 | 210270 | 381439 |
| 1.3 mg/kg Q3W | N | 4 | 4 | 4 | 4 |
|  | Mean | 26275 | 29700 | 3529158 | 4901466 |
|  | SD | 2562 | 5883 | 544670 | 1168781 |
| 1.5 mg/kg Q3W | N | 3 | 3 | 3 | 3 |
|  | Mean | 29500 | 33967 | 4397795 | 5991162 |
|  | SD | 2307 | 5244 | 943322 | 2167373 |
| 0.88 mg/kg Q4W | N | 3 | 3 | 3 | 3 |
|  | Mean | 19867 | 18333 | 1904392 | 1808031 |
|  | SD | 4508 | 3840 | 845918 | 501404 |
| 1.1 mg/kg Q4W | N | 3 | 3 | 3 | 3 |
|  | Mean | 23167 | 22667 | 3077252 | 3127534 |
|  | SD | 2155 | 3213 | 577523 | 868258 |
| 1.3 mg/kg Q4W | N | 1 | 1 | 1 | 1 |
|  | Mean | 33600 | 30600 | 3139663 | 4217298 |
|  | SD | - | - | - | - |

The results showed that the exposure to intact ADC in serum increased with the ARX788 dose, and the exposure to total antibody (TA) in serum also increased largely with the ARX788 dose.

The drug metabolism parameters of intact ADC and total antibody in serum were basically consistent; no significant accumulation trend of intact ADC was observed at doses of 1.1 mg/kg Q3W and below.

**Efficacy results**

As of January 20, 2020, 51 participants had completed more than one tumor assessment and entered the efficacy analysis set. The best objective response was PR. The PR rates in each group were:

- 33.3% (1/3 participants) in the 0.88 mg/kg Q3W group;
- 37.5% (3/8 participants) in the 1.1 mg/kg Q3W group;
- 50% (6/12 participants) in the 1.3 mg/kg Q3W group;
- 63.6% (7/11 participants) in the 1.5 mg/kg Q3W group;
- 33.3% (1/3 participants) in the 1.1 mg/kg Q4W group;
- 75% (3/4 participants) in the 1.3 mg/kg Q4W group.

#### Risk Benefits Analysis

Breast cancer is one of the most predominant malignant tumors in China, and is the most prevalent tumor in women. Epidemiological statistics^[1]^ showed that in 2011, the number of female breast cancer cases in China was about 249,000, with an incidence rate of 37.86/100,000, and the incidence had been on the rise in the last decade. The number of annual deaths was about 60,000, with a mortality rate of 9.21/100,000, and breast cancer mortality had been on the rise in the last decade. The 1-year, 3-year and 5-year observed survival rates in breast cancer patients were 90.5%, 80.0% and 72.7%, respectively, and the 5-year relative survival rate was 73.0% (95% CI was 0.712-0.749). The 2015 Chinese epidemiological prediction data for cancer^[2]^ suggested that breast cancer would remain the most prevalent tumor in women. In 2015, there were about 272,000 new cases of breast cancer and about 70,000 deaths in China. About 3% to 10% of new breast cancer cases each year have distant metastases at the time of diagnosis. Among early-stage patients, 30%-40% may develop into advanced breast cancer, with a 5-year survival rate of only 20% and an overall median survival time of 2-3 years^[3]^.

China has a large number of patients with metastatic breast cancer. The results of a retrospective analysis trial conducted on 422 patients with metastatic breast cancer^[18]^ showed that the most common distant metastatic sites in patients with metastatic breast cancer were bone, lung and liver. Molecular and gene expression analysis showed that breast cancer is a heterogeneous disease including several subtypes, including ductal (luminal A and luminal B), HER2 over-expressing and basal, and that patients with HER2 overexpressing and basal breast cancer have a poorer outcome and are more likely to develop brain metastases than those with ductal breast cancer. HER2 overexpression in 15-20% of breast cancer patients is an important adverse prognostic factor for this disease^[19]^. Based on the above information, it can be seen that patients with HER2-positive metastatic breast cancer generally have a poor prognosis and have a greater need for clinical treatment.

Currently, the main HER2-targeted drug recommended in the Guidelines for the Diagnosis and Treatment of Breast Cancer (2018.V1) is trastuzumab, which is widely used in patients with HER2-positive breast cancer at different stages of progression. The study data^[12]^ on the use of trastuzumab in the first-line treatment of metastatic breast cancer have shown that the time to progression (TTP) of the trastuzumab combined with chemotherapy group was significantly better than that of the chemotherapy group (7.2 vs 4.5 months). However, the study^[20]^ has also suggested that most patients with metastatic breast cancer developed drug resistance and disease progression after receiving trastuzumab treatment, presenting higher requirements for targeted therapy in patients with HER2-positive metastatic breast cancer.

The antibody-coupled drug T-DM1 (Kadcyla^®^) was approved by the FDA on February 22, 2013 for the treatment of HER2-positive advanced metastatic breast cancer that has failed trastuzumab and first-line taxane chemotherapy. Data from its pivotal phase III clinical trial^[21^] showed that T-DM1 significantly improved patient survival compared to lapatinib in combination with capecitabine, with a median PFS of 9.6 vs. 6.4 months and median OS of 30.9 vs. 25.1 months in both treatment groups.

Analysis of safety data^[22]^ showed that among the 884 participants treated with T-DM1, the most frequently reported adverse events were fatigue (46.4%), nausea (43.0%), platelets decreased (32.2%), headache (29.4%), and constipation (26.5%). The most common Grade 3-4 adverse events were platelets decreased (11.9%) and increased AST concentration (4.3%), which were manageable and usually unrelated to clinical symptoms. Overall information shows that T-DM1 is well tolerated in the treatment of breast cancer patients.

Similar to the mechanism of action of T-DM1, the investigational product ARX788 (or known as WBP265 ADC) is an antibody-drug conjugate composed of anti-HER2 monoclonal antibody and toxin small molecule AS269. The anti-HER2 monoclonal antibody can specifically bind to human HER2 and AS269 is a highly effective microtubule inhibitor that inhibits cell growth. The results from the phase I trial on the safety, tolerability and pharmacokinetics of ARX788 monotherapy in Chinese participants with HER2 advanced breast cancer showed a favorable safety profile of the investigational product and a tumor treatment response in some dose groups following efficacy assessment, with the best efficacy outcome observed to date being partial response (PR).

Currently, ARX788 has not been approved by the Chinese drug regulatory authority for marketing, so participation in this clinical trial may cause some risks to the participants. During the clinical trial process, measures such as selecting sites with emergency rescue-related procedures and equipment, arranging experienced clinicians, and designing reasonable safety examinations will be taken. It is required to pay close attention to the safety of participants, and provide prompt and reasonable intervention when adverse events occur.

In summary, this trial provides an opportunity for patients with HER2-positive metastatic breast cancer to achieve remission and prolong their lives.

### Study Objectives

#### Primary Objective

To evaluate the efficacy of ARX788 versus lapatinib in combination with capecitabine in the treatment of HER2-positive locally advanced or metastatic breast cancer based on progression-free survival (PFS) evaluated by the Independent Endpoint Review Committee (IRC).

#### Secondary Objectives

1. To further evaluate the efficacy of ARX788 versus the control drug in the treatment of HER2-positive locally advanced or metastatic breast cancer based on the participant’s overall survival (OS), investigator-assessed PFS, objective response rate (ORR, including partial response [PR] and complete response [CR]), disease control rate (DCR), and duration of response (DOR)
2. To further observe the safety of ARX788 in participants with HER2-positive locally advanced or metastatic breast cancer
3. To evaluate the immunogenicity of ARX788 in participants with HER2-positive locally advanced or metastatic breast cancer
4. To evaluate the population pharmacokinetic (PK) profile of ARX788 in participants with HER2-positive locally advanced or metastatic breast cancer

### Trial Design

#### Overall Design

This trial is a randomized, open-label, positive control, phase III clinical trial of ARX788 in participants with HER2-positive locally advanced or metastatic breast cancer.

This trial plans to include 440 participants with HER2-positive locally advanced or metastatic breast cancer who have previously received treatment with taxane and trastuzumab, and the participants will be randomized to the investigational product group or the control drug group in a 1:1 ratio to receive the investigational product alone or the lapatinib in combination with capecitabine.

ARX788 will be administered at a dose of 1.5 mg/kg once every 3 weeks (Q3W) in the investigational product group, and standard doses of lapatinib and capecitabine will be administered in the control drug group. All participants will receive long-term administration until intolerable toxicity or disease progression or death or voluntary withdrawal or the end of this trial (defined as trial completion or early trial discontinuation). If, after obtaining updated information on additional clinical trials of ARX788, the Investigator and Sponsor agree that other doses may have better efficacy and a manageable safety profile, some or all of the participants in the investigational product group may be administered with other dosing regimens.

During the treatment period, all participants will be assessed for anti-tumor efficacy every six weeks based on their disease status as determined by the IRC and the Investigator respectively as per RECIST1.1 criteria until disease progression or death or refusal to come the hospital for follow-up or the end of the trial (including the completion and early discontinuation of the trial), whichever occurs first. After a participant experiences disease progression or refuses to come to the hospital for follow-up, the Investigator will conduct a telephone visit every 3 months (30 days per month) to obtain the participant’s survival information and whether he/she is receiving other anti-tumor treatments. PK samples will be collected and tested in participants in the investigational product group to assess the population PK profile of ARX788 in patients with HER2-positive advanced breast cancer. If conditions permit, population PK samples should be collected and tested in all participants in the investigational product group. All participants in the investigational product group will receive immunogenicity testing to assess the potential effect of the immunizing antigen production on the efficacy and safety of the investigational product.

In this trial, the primary analysis of PFS will be conducted when 335 PFS events assessed by the Independent Review Committee (IRC) occur. Two interim analyses will be set up during the trial. The first interim analysis will be performed when 160 participants complete the Cycle 4 visit, and futility will be determined based on the ORR difference to decide whether to continue the enrollment; the second interim analysis will be performed when 224 (2/3) IRC-assessed PFS events occur, and superiority test and sample size re-estimation will be performed.

#### Selection of Trial Population and Justification

This trial plans to enroll HER2-positive locally advanced or metastatic breast cancer participants, i.e., participants who have received ≤ two lines of chemotherapy (excluding hormone therapy) for recurrent or metastatic diseases before enrollment. HER2-positive is defined as FISH+ or IHC 3+.

Breast cancer is the most common malignant tumor in women worldwide. In 2015, there were about 272,000 new cases of breast cancer and about 70,000 deaths in China. About 3% to 10% of new breast cancer cases each year have distant metastases at the time of diagnosis. Among early-stage patients, 30%-40% may develop into advanced breast cancer, with a 5-year survival rate of only 20% and an overall median survival time of 2-3 years^[3]^. HER2 overexpression in 15-20% of breast cancer patients is an important adverse prognostic factor for this disease^[18]^. The treatment of such patients has improved significantly since the approval of trastuzumab for the treatment of HER2-positive breast cancer.

Trastuzumab binds to the extracellular ligand-binding domain IV of HER2 protein to exert its anti-tumor effect by blocking HER2-mediated mitogenic signaling. The study data on the use of trastuzumab in the first-line treatment of metastatic breast cancer have shown that the time to progression (TTP) of the trastuzumab combined with chemotherapy group was significantly better than that of the chemotherapy group (7.2 vs 4.5 months). However, the study^[20]^ data have also suggested that most patients with metastatic breast cancer developed drug resistance and disease progression after receiving trastuzumab treatment, presenting higher requirements for targeted therapy in patients with HER2-positive metastatic breast cancer.

T-DM1 (Kadcyla^®^) is an antibody-drug conjugate (ADC) composed of trastuzumab combined with small molecule microtubule inhibitor DM1. T-DM1 produces the same physiological activity as trastuzumab^[23^], and blocks HER2-mediated mitogenic signaling by binding to extracellular ligands of HER2 protein. T-DM1 also enters the cells via endocytosis by target binding to HER2 and releases the active cytotoxic payload DM1 within HER2-expressing cells after lysosomal degradation, thus greatly reducing the toxicity produced by whole-body exposure of DM1^[24^]. T-DM1 was approved by the FDA on February 22, 2013 for the treatment of HER2-positive metastatic breast cancer after trastuzumab and taxane chemotherapy. In 2018, the CSCO Guidelines for the Diagnosis and Treatment of Breast Cancer (2018.V1) pointed out that T-DM1 is the international standard second-line anti-HER2 treatment regimen. T-DM1 has not yet been marketed in China, but patients should be encouraged to enter clinical trials to achieve the best survival benefit.

Similar to the mechanism of action of T-DM1, the investigational product ARX788 (or known as WBP265 ADC) is an antibody-drug conjugate composed of anti-HER2 monoclonal antibody and toxin small molecule AS269. The anti-HER2 monoclonal antibody can specifically bind to human HER2 and AS269 is a highly effective microtubule inhibitor that inhibits cell growth. The results from the phase I trial on the safety, tolerability and pharmacokinetics of ARX788 monotherapy in Chinese participants with HER2 advanced breast cancer showed a favorable safety profile of the investigational product and a tumor treatment response in the target dose group following efficacy assessment.

Taken together, ARX788 is expected to have a good therapeutic effect in patients with HER2-positive locally advanced or metastatic breast cancer, and can provide a new option for the treatment of such patients.

#### Selection of Administration Dose and Justification

The phase I clinical trials have been conducted in Australian and Chinese participants with HER2-positive advanced breast cancer, respectively, to fully explore the tolerability, safety, efficacy and pharmacokinetic profile of ARX788. The preliminary results from the two phase I trials are summarized as follows:

- Phase I trial in Australia: In the dose-escalation, phase I clinical trial of ARX788 monotherapy in the treatment of HER2-positive (in situ hybridization [FISH]+ or IHC 3+) advanced breast cancer (clinicaltrial.gov registration number NCT02512237) in Australia, the dose groups explored and the corresponding number of participants were 0.33 mg/kg (1 participant), 0.66 mg/kg (1 participant), 1.3 mg/kg (3 participants), 2.20 mg/kg (3 participants), and 2.9 mg/kg (1 participant), respectively. The efficacy results showed that 1 participant (100%) in the 0.66 mg/kg group, 1 participant (33%) in the 1.3 mg/kg group, and 1 participant (33%) in the 2.2 mg/kg group were determined to have partial response (PR) after efficacy assessment. The dose-limiting toxicity (DLT) first appeared in the 2.9 mg/kg dose group, which was grade 3 mucosal inflammation. Delayed toxicity occurred after 4 to 5 cycles of drug administration in the ≥1.3 mg/kg dose groups (the delayed toxicity was subsequently determined as delayed DLT):
  - One participant in the 1.3 mg/kg group experienced Grade 2 non-infectious pneumonia at Cycle 4 treatment that improved after drug discontinuation and steroid therapy;
  - Two participants in the 2.2 mg/kg group developed Grade 2 and Grade 5 non-infectious pneumonia after 4 to 5 cycles of treatment, respectively.
- Phase I trial in China: The phase I clinical trial of ARX788 monotherapy in the treatment of participants with HER2-positive advanced breast cancer in China (drug clinical trial registration number CTR20171162) is ongoing, with 51 (100%) of the 51 participants treated with at least one investigational product meeting efficacy and safety assessments as of January 20, 2020. The dosing regimens completed/being explored in the study and the corresponding sample sizes are as follows:
- 0.33 mg/kg Q3W (3 participants);
- 0.66 mg/kg Q3W (3 participants);
- 0.88 mg/kg Q3W (3 participants);
- 1.10 mg/kg Q3W (8 participants);
- 1.30 mg/kg Q3W (12 participants);
- 1.50 mg/kg Q3W (11 participants);
- 0.88 mg/kg Q4W (4 participants);
- 1.10 mg/kg Q4W (3 participants);
- 1.30 mg/kg Q4W (4 participants).

There were no DLTs among the participants in the groups that had completed dose-limiting toxicity (DLT) assessment. No adverse events leading to the death of participants were reported, and 4 participants experienced Grade 3 or above treatment emergent adverse events (TEAEs) related to the investigational product: 1 in the 0.88 mg/kg Q3 group, 2 in the 1.1 mg/kg Q3W group, and 1 in the 1.5 mg/kg Q3W group. Of the 49 participants who experienced TEAEs related to the investigational product, 91.8% had a maximum severity of Grade 2, and there was no apparent increasing trend in the incidence of AEs corresponding with the dose escalation.

The best response in participants who have been assessed was PR, and PR did not occur in participants in the 0.66 mg/kg Q3W and below dose groups. The PR rate was 33.3% (1/3 participants) in the 0.88 mg/kg Q3W group, 37.5% (3/8 participants) in the 1.1 mg/kg Q3W group, 50% (6/12 participants) in the 1.3 mg/kg Q3W group, and 63.6% (7/11 participants) in the 1.5 mg/kg Q3W group. The PR rate showed a tendency to increase with dose.

Based on the above safety and efficacy data, the dosing regimen of ARX788 in this phase III clinical trial is preliminarily set as 1.5 mg/kg Q3W. Alternative dosing regimens may be used for some or all participants in this trial if, after obtaining updated information from other clinical trials of the investigational product, the Investigator and the Sponsor mutually agree that alternative dosing and/or dosing regimens may have better efficacy and a manageable safety profile.

#### Toxicity-Based Dose Modification

Participants who experience clinically significant adverse events (AEs), or intolerable toxicity, may receive necessary interventions and supportive treatment. The Investigator will determine whether a participant in the investigational product group requires dose modification of ARX788, delayed dosing, or withdrawal from the study based on the grade of adverse events occurring after treatment, recovery, and correlation with ARX788 as per the relevant criteria specified in the Protocol. Toxicity-based dose modifications for participants in the ARX788 group can be found in the following table:

Table 3 Toxicity-based dose modification in participants in the ARX788 group

| **AE** | **Grade** | | **Dose Modification Principle** |
| --- | --- | --- | --- |
| Increased transaminase  (AST/ALT) | Grade 2 (> 3, ≤ 5 times the upper limit of normal [ULN]) | | No dose modification |
|  | Grade 3 (>5, ≤20 times ULN) | | Withhold ARX788 until AST/ALT level recovers to ≤ Grade 2, and continued at a reduced dose level |
|  | Grade 4 (> 20 times ULN) | | Permanently discontinue treatment with ARX788 |
| Elevated bilirubin | | Grade 2 (> 1.5, ≤ 3 times ULN) | Withhold ARX788 and resume at the original dose when the total bilirubin level recovers to ≤ Grade 1 |
|  |  | Grade 3 (>3, ≤10 times ULN) | Withhold ARX788 use and continue at a reduced dose when the total bilirubin level recovers to ≤ Grade 1 |
|  |  | Grade 4 (>10 times ULN) | Permanently discontinue treatment with ARX788 |
| Platelets decrease | | Grade 2 (50,000-75,000/mm^3^) | Withhold ARX788 use if the condition does not recover to ≤ grade 1 or worsens to grade 3 within 2 weeks. And it continues at the original dose if the platelet level recovers to ≤ grade 1 (≥ 75,000/mm^3^). |
|  |  | Grade 3 (25,000-50,000/mm^3^) | Withhold ARX788 use and continue at the original dose when the platelet level recovers to ≤ Grade 1 (≥ 75,000/mm^3^). |
|  |  | Grade 4 (< 25,000/mm^3^) | Withhold ARX788 use and continue at a reduced dose when the platelet level recovers to ≤ Grade 1 (≥ 75,000/mm^3^). |
| Left ventricular dysfunction | | Symptomatic congestive cardiac failure | Permanently discontinue ARX788 use |
|  |  | LVEF < 40% | Withhold ARX788 use, and permanently discontinue ARX788 use if LVEF < 40% after re-assessment of LVEF within 3 weeks. |
|  |  | LVEF of 40% to ≤45%, ≥10% reduction from baseline | Withhold ARX788 use, and permanently discontinue ARX788 use if LVEF does not recover to within 10% from baseline after re-assessment of LVEF within 3 weeks. |
|  |  | LVEF of 40% to ≤45%, <10% reduction from baseline | Continue with the original dose of ARX788 and intensify LVEF monitoring. |
|  |  | LVEF > 45% | Continue with the original dose of ARX788 |
| Pulmonary toxicity (interstitial lung disease or pneumonitis, etc.) | | Grade 1~2 | ARX788 should be on hold, as determined by the investigator until the event recovers to ≤ Grade 1 within 12 weeks. Otherwise, permanently discontinue ARX788 treatment. |
|  |  | Grade 3 or 4 | Permanently discontinue ARX788 treatment. |
| Ocular toxicity (keratitis or dry eye, etc.) | | Grade 2 | If a Grade 2 ocular adverse event persists for more than 2 weeks, continue ARX788 administration at a reduced dose subsequently at the Investigator’s discretion. If the Grade 2 ocular adverse event does not resolve after the dose reduction, withhold the administration of ARX788, and if the adverse event level recovers to ≤ Grade 1 within 12 weeks, continue administration at a reduced dose at the Investigator’s discretion. |
|  |  | Grade 3 | Withhold ARX788 use and continue administration at a lower dose if the adverse event levels recover to ≤ Grade 1 within 12 weeks, as determined by the Investigator. If a grade ≥2 ocular adverse event occurs after dose reduction, permanently discontinue ARX788 treatment and the participant withdraws from the study. |
|  |  | Grade 4 | Permanently discontinue ARX788 treatment and the participant withdraws from the study. |
| Other adverse events | |  | The Investigator determines ARX788 dose modification, delayed administration or withdrawal from the study according to the participant’s clinical symptoms. |

For the participants in the control drug group, refer to the package inserts of lapatinib and capecitabine as well as clinical routine procedures for the management of TEAEs, dose modification or delayed administration.

If the participant experiences a dose reduction due to toxicity during the treatment period, then the reduced dose can be used for subsequent continuous treatment. Dose recovery is not allowed after the recovery of toxicity in the investigational product group participants and the control drug group participants. Participants are allowed a maximum of 3 dose level adjustments in the investigational product group, with the following dose level adjustments referenced:

Table 4 ARX788 dose modification levels

| **Dose level** | **Dose** |
| --- | --- |
| 0 | 1.5 mg/kg Q3W |
| -1 | 1.3 mg/kg Q3W |
| -2 | 1.1 mg/kg Q3W |
| -3 | 0.88 mg/kg Q3W |
| Dose modification at lower level | Permanent discontinuation |

#### Population Pharmacokinetic Study

PK data from the phase I study of ARX788 in the treatment of advanced breast cancer showed that exposure to intact ADC, total antibody and metabolite pAF-AS269 in the serum of participants administered at 1.5 mg/kg Q3W increased with doses of ADC (ARX788) in both Cycle 1 and Cycle 3, with intact ADC at 154 h T_1/2_ in Cycle 3. PK samples will be collected and tested in participants in the investigational product group to assess the population PK profile of ARX788 in patients with HER2-positive advanced breast cancer. If conditions permit, population PK samples should be collected and tested in all participants in the investigational product group.

#### Immunogenicity

The ARX788 contains an anti-HER2 monoclonal antibody, and participants with locally advanced or metastatic breast cancer enrolled in this trial have previously received trastuzumab targeted therapy. Therefore, some participants may have corresponding antibodies in the body before ARX788 treatment, and antibodies may also be produced after ARX788 treatment. Antibodies before and after treatment may affect the efficacy and safety of treatment. Therefore, blood samples will be collected during the screening period and at different times after drug administration for immunogenicity study in participants in the investigational product group, and whether there is a potential link between the antibody production and the safety and efficacy will be analyzed.

In the trial, blood samples will be collected from all participants in the investigational product group for immunogenicity study before the first dose, every 2 treatment cycles after the first dose, and at the last visit.

#### Blinding and Unblinding

This trial is an open-label study and does not involve the blinding and unblinding processes.

#### Randomization

Eligible participants will be randomized to the investigational product group or the control drug group by means of stratified block randomization at a ratio of 1:1, using the number of prior chemotherapy lines (0-1, >1) received for recurrent or metastatic lesions and whether they have concomitant visceral metastases (yes, no) as stratification factors.

Each participant in the trial will enter either the investigational product group or the control drug group as determined by a randomization table. The statistician will use the PLAN process in SAS 9.4 and above to generate a randomization table and assign the participants to either the investigational product group or the control drug group in a 1:1 ratio. The random data are reproducible and the set initial seed for random numbers need to be stored.

After confirming that a participant meets the randomization criteria, the site will conduct randomization of the participant and assign the participant a randomization number. The Investigator will administer the drug to the participant based on the participant’s randomization number.

#### End of Trial

The end of the trial includes both the completion of the trial and the early discontinuation of the trial. In either case the trial will be stopped and all participants will be discharged from the group and will no longer receive the investigational product treatment.

The completion of the trial is defined as the time when the last participant completes the last visit. The Sponsor has the right to terminate the trial early for reasonable reasons at any time point throughout the trial.

### Study Population

#### Inclusion Criteria

Any participant who meets all of the following criteria can be enrolled:

1. The participant or his/her legal representative agree to voluntarily signs the informed consent form in writing;
2. 18 to 75 years old (including upper and lower limits), male or female;
3. Has a cytological or histological diagnosis of breast cancer (BC), with unresectable locally advanced, recurrent or metastatic BC;
4. Has previously received ≤ two lines chemotherapy (excluding hormone therapy) for recurrent or metastatic BC;
5. Must meet at least one of the following conditions:
   1. (Neoadjuvant) adjuvant therapy, receiving treatment containing trastuzumab (or marketed trastuzumab biosimilars) ± pertuzumab for ≥ 9 weeks, with disease recurrence or progression during treatment or within 12 months after the end of treatment;
   2. Recurrent or metastatic disease treated with at least one treatment regimen containing trastuzumab (or marketed trastuzumab biosimilars) ± pertuzumab, with disease progression during or after treatment.
6. Has previously received treatment with taxane;
7. Has at least one measurable target lesion as per RECIST1.1 criteria;
8. Can provide tissue samples for HER2 testing that are determined to be qualified by the central laboratory during the screening period;
9. Tissue samples determined to be HER2 positive (defined as IHC3+ or FISH+) by central laboratory testing;
10. ECOG Performance Status Score of 0-1;
11. LVEF ≥ 50% as shown by echocardiography during the screening period;
12. Has recovered from any AE (≤ Grade 1) related to prior surgery and prior cancer treatment, except for the following:
    1. alopecia; b. pigmentation; c. long-term toxicity caused by radiotherapy, which cannot be recovered based on the assessment of the Investigator; d. Grade 2 or lower neurotoxicity caused by platinum; e. hemoglobin 90-100 g/L (including boundary values).
13. Adequate bone marrow, liver, kidney and coagulation function (please refer to the upper limit of normal of each site):

- Bone marrow (no blood transfusion or use of adjuvant drugs to increase white blood cells and platelets within 2 weeks prior to screening):
  - Absolute neutrophil count (ANC) ≥ 1.5 × 10^9^/L;
  - Platelet ≥ 100 × 10^9^/L;
  - Hemoglobin ≥ 90 g/L;
- Liver function:
- Bilirubin total ≤ 1.5 times the upper limit of normal (ULN);
- Alanine aminotransferase (ALT) and aspartate aminotransferase (AST) ≤ 3 times the ULN, alkaline phosphatase (ALP) ≤ 2.5 times the ULN; ALT and AST ≤ 5 times the ULN in the presence of liver metastasis, and ALP ≤ 5 times the ULN in the presence of bone metastasis;
- Renal function: Creatinine ≤ 1.5 times the ULN;
- Coagulation: International normalized ratio (INR) ≤ 1.5 times the ULN, and activated partial thromboplastin time (APTT) ≤ 1.5 times the ULN;

1. Expected survival ≥ 3 months.

#### Exclusion Criteria

Any participant who meets any of the following criteria can not be enrolled:

1. Has known history to be allergic to any active ingredient or excipient of ARX788; a clear history of allergy to protein drugs; a history of specific allergies (asthma, rheumatism, eczematous dermatitis), or has experienced other serious allergic reactions and is not suitable for treatment with ARX788 as determined by the Investigator;
2. Has known hypersensitivity or delayed allergic reactions to certain components of capecitabine and lapatinib or similar drugs, or known contraindications to capecitabine, mainly including previous severe and unexpected reactions to fluoropyrimidine or known allergy to fluorouracil and known complete deficiency of dihydropyrimidine dehydrogenase (DPD) activity;
3. Has previously used any treatment regimen containing capecitabine and/or anti-HER2 TKI, except for any of the following:
   1. (Neoadjuvant) adjuvant therapy containing capecitabine and/or anti-HER2 TKI, with disease recurrence more than 12 months after completion of full treatment with capecitabine and/or anti-HER2 TKI. The neoadjuvant treatment regimen must be effective, which refers to clinical CR or PR or pathological CR. In addition, patients have not been exposed to capecitabine or anti-HER2 TKI during the recurrence or metastasis setting;
   2. Recurrent or metastatic disease, treated effectively with a regimen containing capecitabine and/or anti-HER2 TKI, but discontinued not for disease progression or drug-related adverse reaction and at a period of more than 6 months with no evidence of disease progression. Moreover, patients have not been exposed to capecitabine or anti-HER2 TKI during the (neoadjuvant) adjuvant treatment phase.
4. Has previously received T-DM1 or other HER2-ADC drugs;
5. Suffered from another malignant tumor in the past 5 years, except for cervical cancer in situ or non-melanoma skin cancer that has received curative treatment;
6. Has primary central nervous system (CNS) malignancy or CNS metastasis that has failed local treatment, except that patients who have asymptomatic brain metastasis, or have stable clinical symptoms that do not require steroids and other treatment for brain metastases for ≥ 28 days prior to the first administration of the investigational product are allowed to be enrolled;
7. Has interstitial lung disease requiring steroid therapy, a history of drug-induced interstitial lung disease, a history of radiation pneumonitis, or any evidence indicating clinically active interstitial lung disease;
8. Has any eye disease that require medical intervention such as keratitis, corneal disorder, retinal disorder or active eye infection;
9. Is unwilling or unable to stop wearing corneal contact lens during the study;
10. Has cardiac insufficiency, including but not limited to congestive cardiac failure, transmural myocardial infarction, angina pectoris requiring drug therapy, clinically significant heart valve disorders, and high-risk arrhythmia, or clinically significant QTc abnormalities in the ECG examination during the screening period (QTc > 450 msec [male] or QTc > 470 msec [female] corrected on ECG at rest);
11. Uncontrolled hypertension (at rest: systolic blood pressure > 160 mmHg or diastolic blood pressure > 100 mmHg);
12. Has evidence of severe or uncontrollable systemic diseases (e.g., unstable or uncompensated respiratory, cardiac, hepatic, or renal disease) as determined by the Investigator;
13. Has used chemotherapy, radiotherapy or immunotherapy within 4 weeks prior to the first administration of the investigational product (the use of physiological replacement dose of corticosteroid [prednisone or equivalent < 15 mg/day] is permitted);
14. Has received breast cancer endocrine therapy within 2 weeks before the first administration of the investigational product;
15. Has received palliative radiotherapy for bone metastases within 2 weeks prior to the first administration of the investigational product;
16. Prior exposure to anthracyclines accumulated to doses of:

- Doxorubicin or liposomal doxorubicin > 500 mg/m^2^;
- Epirubicin > 900 mg/m^2^;
- Mitoxantrone > 120 mg/m^2^;
- Others (i.e., liposomal doxorubicin or other anthracyclines > the equivalent of 500 mg/m^2^ of doxorubicin);
- If more than one anthracycline is used, then the cumulative dose must not exceed the equivalent of 500 mg/m^2^ of doxorubicin.

1. Has any uncontrolled infection, or other situations that may limit study compliance or interfere with the evaluation;
2. Is positive for hepatitis B surface antigen and HBV DNA ≥ ULN, or positive for any one of hepatitis C virus antibody, syphilis spirochete antibody, or human immunodeficiency virus antibody test result;
3. Plans to receive major surgical treatment or experiences severe traumatic injury within 2 weeks before the first use of the investigational product or during the trial;
4. A pregnant or breastfeeding female participant;
5. A woman of childbearing potential (WOCBP) (WOCBPs include: any woman participant who has had her menarche, and has not received successful artificial sterilization surgery [hysterectomy, bilateral tubal ligation, or bilateral oophorectomy] or has not menopausal) or a male participant who is unwilling or unable to use an acceptable method of contraception throughout the entire treatment period of this trial and within 8 months after the last dose of the investigational product;
6. Participated in any other clinical trial and used any other investigational product within 4 weeks prior to the first administration of the investigational product;
7. Has any mental or cognitive disorder that may restrict his/her understanding and execution of the informed consent form;
8. Other conditions that the Investigator considers inappropriate for participation in this trial, such as poor compliance.

#### Criteria for Treatment Discontinuation/Withdrawal

During the trial, a participant may voluntarily withdraw from the study due to reasons such as loss to follow-up or withdrawal of consent and request for withdrawal. The Investigator may allow the participant to discontinue the trial treatment/withdraw from the trial if any of the following occurs:

- - 1. The Investigator considers that the participant should discontinue the investigational product treatment for safety considerations (e.g., the participant experiences an intolerable adverse events);
    2. The participant is pregnant;
    3. The participant experiences disease progression;
    4. The participant experiences a significant protocol violation that, in the opinion of the Investigator, significantly affects the evaluation of the primary endpoints of this trial, and the Investigator believes that the participant should discontinue treatment;
    5. Other conditions that the Investigator determines make the participant unfit to continue in the trial.

#### Criteria for Early Termination of Trial

Early discontinuation of the trial means that the clinical trial has not yet been completed for all participants according to the Protocol and the entire trial or a part of the trial is discontinued midway. The purpose of trial discontinuation is mainly to protect the rights and interests of the participants, ensure the quality of the trial, and avoid unnecessary economic losses.

Under normal circumstances, the trial will not be prematurely terminated at will. However, the entire trial or a part of the trial (such as the trial in a site) may be prematurely terminated if any of the following occurs:

- - 1. The total sample size for competitive enrollment has met the requirements of the trial, but the site has not completed the planned enrollment as contracted;
    2. The investigator at the site cannot follow the Protocol, GCP, etc.;
    3. Access to new information leads to an unfavorable risk-benefit evaluation of ARX788, including sufficient evidence to suggest a lack of efficacy or an unacceptable safety profile;
    4. The collaborating institution does not consider it appropriate to continue this trial due to medical, ethical or commercial reasons, etc.;
    5. The enrollment of participants is very poor and it is impossible to complete the trial in an acceptable time frame;
    6. The National Medical Products Administration or the Ethics Committee orders the discontinuation of the trial for some reason.

Any early discontinuation of the clinical trial shall be promptly notified in writing to all parties (Sponsor, Research Institution, Ethics Committee, clinical trial institution and administrative authority).

#### Participant Assignment and Number

After the participant signs the informed consent form and before the relevant trial examinations, the screening number is obtained in order. The screening number is made up of the uppercase letter “S” + 5 digits: The first 2 digits are the “site number”, and the last 3 digits are the “screening serial number”. For example, the screening number of the second screened participant at Site 01 is “S01002”.

Any participant who passes the screening will be given a unique randomization number, which may not be reused once assigned.

If the participant withdraws from this trial, his/her randomization number cannot be reused.

### Investigational Product Information

#### Basic Information of Investigational Product

ARX788 is an antibody-drug conjugate formed by covalent binding of a humanized anti-HER2 monoclonal antibody and AS269 (small molecule cytotoxin drug), and the conjugation ratio is fixed at 1:2. The investigational product has two dosage forms, namely, injection solution and sterile powder for injection. In this trial, only the sterile powder for injection is used. The main drug information is as follows:

- **Name:**  Recombinant humanized anti-HER2 monoclonal antibody-AS269 conjugate for injection
- **Code**: ARX788
- **Strength**: 50 mg/vial
- **Dosage and administration**: Intravenous infusion, 1.5 mg/kg, Q3W
- **Storage conditions**: 2°C-8°C, protected from light
- **Transportation and storage conditions**: Cold chain transportation at 2°C-8°C
- **Shelf-life**: Tentatively 18 months
- **Excipients**: Histidine, histidine hydrochloride, trehalose and polysorbate 80
- **Provider**: NovoCodex Biopharmaceuticals Co., Ltd.

#### Information on Other Investigational Products

##### Control Drug - Lapatinib

- - - - **Name:**  Lapatinib mesylate tablet
      - **Strength**: 250 mg
      - **Dosage form:** Tablet
      - **Dosage and administration**: The recommended dose is 1,250 mg, taken orally once a day, every 21 days as a cycle. It is recommended to take the daily dose in one lump, not in separate doses. It should be taken at least 1 hour before meal or at least 1 hour after meal. In combination with lapatinib, the dose of capecitabine is 2,000 mg/m^2^/d, taken orally in 2 doses, with an interval of about 12 hours, administered for 14 consecutive days and rest for 7 days, with 21 days as a cycle. It should be taken with food or within 30 minutes after a meal.
      - **Method of storage:** See the package insert for details
      - **Provider**: NovoCodex Biopharmaceuticals Co., Ltd.

##### Control Drug - Capecitabine

- - - - **Name:**  Capecitabine tablet
      - **Strength**: 0.5 g
      - **Dosage form:** Tablet
      - **Dosage and administration**: See the dosage and administration of lapatinib
      - **Method of storage:** See the package insert for details
      - **Provider**: NovoCodex Biopharmaceuticals Co., Ltd.

#### Investigational Product Administration

**Investigational product group**

- ARX788: The dose is 1.5 mg/kg, and the first infusion duration is 90 ± 10 min. If it is well tolerated without infusion reaction, the subsequent infusion may be shortened to 60 ± 10 min in the Investigator’s judgment. The drug is administered on Day 1 of each cycle for long-term treatment according to Q3W. Weight will be re-measured in each cycle. If the weight change from the last measurement or baseline is >± 10% (whichever is greater), the specific dose should be recalculated and the weight at the new dose should be used as the baseline for subsequent calculations.

**Control drug group**

- Lapatinib: The recommended dose is 1,250 mg, taken orally once a day, every 21 days as a cycle. It is recommended to take the daily dose in one lump, not in separate doses. It should be taken at least 1 hour before meal or at least 1 hour after meal.
- Capecitabine: In combination with lapatinib, the dose of capecitabine is 2,000 mg/m^2^/d, taken orally in 2 doses, with an interval of about 12 hours, administered for 14 consecutive days and rest for 7 days, with 21 days as a cycle. It should be taken with food or within 30 minutes after a meal. Weight will be re-measured in each cycle. If the body surface area change from the last measurement or baseline is >± 10% (whichever is greater), the specific dose should be recalculated and the body surface area at the new dose should be used as the baseline for subsequent calculations.

If a patient misses a dose on a particular day, the dose does not need to be doubled on the second day; the dose can be continued at the next dose time as scheduled. Treatment should continue until disease progression or intolerable toxicity.

#### Packaging and Labeling of Investigational Product

In accordance with the requirements of GCP and national laws and regulations, NovoCodex Biopharmaceuticals Co., Ltd. will be responsible for affixing labels on the packaging boxes of the investigational products, and the investigational products of all participants will be uniformly packaged.

The contents of the trial label may include: Protocol number, Sponsor name, packaging configuration, drug storage conditions for batch number, shelf life, etc. The specific packaging shall be based on the actual product.

#### Management of Investigational Products

The Sponsor will provide the pharmacy manual to clarify the use, storage and related records of the investigational products. It should formulate the supply and management procedures for the investigational products, including the receipt, storage, distribution, use and recovery of the investigational products. Investigational drugs that are recovered from participants and unused by the study staff should be returned to the Sponsor, or destroyed by the clinical trial institution after being authorized by the Sponsor.

The Sponsor should ensure the timely delivery of investigational products to the Investigator and the clinical trial institution to ensure timely use by participants; maintain records of transport, receipt, distribution, recovery and destruction of investigational products; establish a management system for the recovery of investigational products to ensure the recall of defective products, recovery after the end of the trial, and recovery after expiration; and establish a destruction system for unused investigational products. There should be written records of the management process of all investigational products which should be counted accurately throughout the process.

#### Concomitant Medications

The Investigator may use supportive care or adjunctive therapy during the trial depending on the participant’s adverse event status and clinical needs. Supportive care may include but is not limited to antiemetics, opioid or non-opioid analgesics, granulocyte colony-stimulating factors and erythrocyte growth factors.

To fully expose the safety profile of the investigational product, prophylactic medications for possible adverse events (e.g., rash, alopecia, ocular toxicity, etc.) of the investigational product is not permitted throughout the course of the trial. Prophylactic medications are allowed for infusion reactions that may be caused by the investigational product. If in order to prevent participants from experiencing influenza-like infusion reactions, routine prophylactic infusion medications (such as phenergan and/or indomethacin) can be administered 30 minutes before the start of the infusion of the investigational product upon the investigator’s judgment.

Participants cannot receive any concurrent anti-cancer drug treatment (except for drugs such as bisphosphonates used for bone metastasis treatment), including radiotherapy, chemotherapy, biologic therapy, hormone therapy or any other investigational drugs during the entire treatment period from their enrollment into the screening of this trial, and herbal medicines approved for anti-tumor use cannot be used during the course of this trial.

In the study, all AEs, concomitant medications and reasons for treatment, dose/procedures, etc. of the participants must be fully recorded in eCRFs.

### Study Procedures

This trial is divided into the screening period, treatment period, discontinuation follow-up period and long-term follow-up period. All participants should complete the relevant visits and clinical examinations or observations within the specified time in the Protocol.

#### Screening Period (D-28 - D-1)

Participants or their legally acceptable representatives must sign an informed consent form prior to all screening procedures on a voluntary basis. Participants who meet all inclusion criteria and do not meet the exclusion criteria can participate in the trial.

The main procedures or examinations that participants will undergo during the screening period include:

- Signing of informed consent form;
- Judgment of inclusion and exclusion criteria;
- Collection of demographic information;
- Medical history collection, including but not limited to:
  - Historical data on diagnosis of breast cancer by histopathology or cytology;
  - Molecular typing diagnostic data (may include hormone receptor status, etc.);
  - History of malignancy within 5 years before signing the informed consent form;
  - Presence of previous or current interstitial lung disease requiring hormone therapy, a history of drug-induced interstitial lung disease, a history of radiation pneumonitis, or any evidence indicating clinically active interstitial lung disease;
  - Presence of previous or current cardiac insufficiency, including but not limited to congestive cardiac failure, transmural myocardial infarction, angina requiring drug therapy, clinically significant heart valve disorders, and high-risk arrhythmias;
  - Presence of previous or current serious or clinically significant systemic disease, such as cardiac, pulmonary, metabolic, or hepatic or renal disease.
- Collection of treatment history, including at least:
  - Prior anti-tumor treatment;
  - Medication within 4 weeks prior to randomization, including at least the use of corticosteroid.
- Tissue sample acquisition for HER2 expression testing and central laboratory testing;
- Ophthalmological examination, general examination of the eye, including examination of the ocular appendages and the anterior segment of the eye;
- Vital signs;
- Physical examination;
- ECOG score, see Appendix 2 for details;
- Pregnancy test (for women of childbearing potential only);
- Blood routine;
- Blood biochemistry;
- Coagulation test;
- Urinalysis;
- 12-lead ECG;
- Echocardiography;
- Serum virology, which may include HBsAg, HBsAb, HBeAg, HBcAb, HBeAb, HBV DNA quantification (only performed in participants with positive hepatitis B surface antigen), HCV antibody test, HIV antibody test, and treponema pallidum test;
- Tumor imaging evaluations, all imaging should be completed within 28 days prior to the first dose and as close to the start of dosing as possible, primarily including:
  - Contrast-enhanced CT or MRI of chest, abdomen and pelvic cavity: Target lesions and non-target lesions should be selected according to the initial scan, and all subsequent scans should use the same method; for patients who are allergic to contrast agents, CT or MRI plain scan can be performed if determined by the Investigator;
  - Head MRI: The head MRI is a mandatory examination during the screening period, and may be performed in the investigator’s judgment during the treatment period;
  - Bone emission computed tomography (ECT): ECT is mandatory during the screening period and is performed during the treatment period at the discretion of the Investigator based on the participant’s specific condition (e.g., presence of bone pain or elevated alkaline phosphatase);
  - The Investigator may decide whether to perform other imaging examinations, such as breast MRI, breast X-ray, breast ultrasound or other imaging examinations of suspected metastatic lesions, based on the signs and status of individual participants.
- Tumor baseline status recorded as per the Response Evaluation Criteria in Solid Tumors (RECIST) Version 1.1 (Appendix 3);
- Records of concomitant medications;
- Records of adverse events.

The blood routine, blood biochemistry, urinalysis and 12-lead ECG must be completed within one week before starting dosing; otherwise, they must be retested within one week before dosing.

The inclusion/exclusion criteria must be reviewed on D-1. Participants who meet the inclusion requirements will be given a randomization number. The time of randomization should be as close as possible to the time of the first administration of the investigational product.

#### Treatment Period - Day 1 (C1D1)

Starting from C1D1, participants will receive long-term continuous administration according to the dose and regimen specified in the Protocol. During the treatment period, it is necessary to dispense/recover the drugs (only applicable to participants in the control drug group) and the patient diary cards to/from the participants every time they come to the hospital. The tests and procedures required for the participants on C1D1 are as follows:

- - - Weight measurement before administration;
    - Immunogenicity blood sample collection (prior to investigational product administration, only applicable to participants in the investigational product group);
    - First dose of the investigational product, of which the infusion time of ARX788 is 90 ± 10 minutes;
    - Infusion reaction observation (only applicable to participants in the investigational product group): The occurrence of adverse events in participants should be closely observed during and after ARX788 infusion, and they should be treated in a timely manner. If infusion-related symptoms (such as fever or chills) occur, the infusion rate can be reduced or the infusion can be discontinued. For specific treatment measures, refer to Appendix 4 Management of Infusion-Related Reactions;
    - Vital signs, to be monitored within 30 minutes before the first dose of the investigational product and 2 h ± 30 min after the completion of administration;
    - Physical examination (before the first dose of the investigational product);
    - 12-lead ECG, to be performed before the first dose (test results within 48 hours before the start of administration are acceptable) of the investigational product and within 2 h ± 30 min after the completion of administration;
    - Records of concomitant medications;
    - Records of adverse events.

#### Treatment Period - Day 7 (C1D7)

During the treatment period, participants will undergo safety and efficacy tests at the specified frequency. The examinations or procedures that should be received on C1D7 are as follows:

- - - Vital signs;
    - Blood routine;
    - Blood biochemistry;
    - 12-lead ECG;
    - Records of concomitant medications;
    - Records of adverse events.

The time window for C1D7 visit is ±1 day, and any situation beyond the time specified in the Protocol should be recorded.

#### Treatment Period - Day 14 (C1D14)

During the treatment period, participants will undergo safety and efficacy tests at the specified frequency. The examinations or procedures that should be received on C1D14 are as follows:

- - - Vital signs;
    - Blood routine;
    - Blood biochemistry;
    - 12-lead ECG;
    - Records of concomitant medications;
    - Records of adverse events.

The time window for C1D14 visit is ±3 days, and any situation beyond the time specified in the Protocol should be recorded.

#### Treatment Period - (C2D1 - discontinuation)

During the treatment period, the following dosing procedures or related examinations will be performed every cycle after the first dose of the investigational product. The time window for a treatment visit in each cycle is ±3 days. The safety tests required to be performed in each cycle should be completed and the investigational product treatment should be continued after the Investigator determines that the administration conditions are met. Any situation beyond the time specified in the Protocol should be recorded.

- - - Weight measurement. If the weight/body surface area change from the last measurement or baseline is >± 10% (whichever is greater), the specific dose of ARX788 (as per the weight) or capecitabine (as per the body surface area) should be recalculated, and the weight or body surface area at the new dose should be used as the baseline for subsequent calculations.
    - Vital signs;
    - Physical examination;
    - Blood routine;
    - Blood biochemistry;
    - Urinalysis;
    - 12-lead ECG;
    - Records of concomitant medications;
    - Records of adverse events.

On Day 1 of each cycle, participants should receive the investigational product, and participants in the investigational product group should also be monitored for infusion-related reactions, and for vital signs and 12-lead ECG after infusion (2 h ± 30 min after completion of dosing). If the first infusion is well tolerated without infusion reaction, the subsequent infusion time of ARX788 may be shortened to 60 ± 10 min in the Investigator’s judgment. If infusion-related symptoms (such as fever or chills) occur, the infusion rate can be reduced or the infusion can be discontinued. For specific treatment measures, refer to Appendix 4 Management of Infusion-Related Reactions.

The following examinations or procedures will be performed every two cycles after the first dose of the investigational product. The time window for the visit is ±3 days, and any situation beyond the time specified in the Protocol should be recorded.

- - - Vital signs;
    - Physical examination;
    - 12-lead ECG;
    - Blood routine;
    - Blood biochemistry;
    - Urinalysis;
    - Coagulation test;
    - Pregnancy test (for women of childbearing potential only);
    - Echocardiography;
    - ECOG score;
    - Serum virology (Hepatitis B five-item test ± HBV DNA); hepatitis B five-item test will only be performed in participants with positive hepatitis B surface antigen during the screening period; the Investigator will determine whether HBV DNA quantitative test is required;
    - Immunogenicity blood sample collection (only applicable to participants in the investigational product group);
    - Records of concomitant medications;
    - Records of adverse events.

During the treatment period, participants will undergo a tumor efficacy assessment every six weeks after randomization, with a time window of ±3 days. Any situation beyond the time specified in the Protocol should be recorded. The main procedures include the following:

- - - Tumor imaging mainly includes the following examinations:
      - Contrast-enhanced CT or MRI of chest, abdomen and pelvic cavity: Target lesions and non-target lesions should be selected according to the initial scan, and all subsequent scans should use the same method; for patients who are allergic to contrast agents, CT or MRI plain scan can be performed if determined by the Investigator;
      - Head MRI: The head MRI is a mandatory examination during the screening period, and may be performed in the investigator’s judgment during the treatment period;
      - Bone emission computed tomography (ECT): ECT is mandatory during the screening period and is performed during the treatment period at the discretion of the Investigator based on the participant’s specific condition (e.g., presence of bone pain or elevated alkaline phosphatase);
      - The Investigator may decide whether to perform other imaging examinations, such as breast MRI, breast X-ray, breast ultrasound or other imaging examinations of suspected metastatic lesions, based on the signs and status of individual participants.
    - Anti-tumor efficacy assessments will be performed according to the RECIST 1.1;

The Investigator may add examinations based on the participant’s signs or disease conditions, including imaging examinations other than those specified in the Protocol (every 6 weeks), and all examinations should be recorded.

#### Follow-up Period After Treatment Discontinued (within 28+7 days after discontinuation)

Participants will continue to use the drug until intolerable toxicity or disease progression or death or voluntary withdrawal or the end of this trial. The Investigator may decide whether the participant will continue to receive the trial treatment based on the Investigator-assessed disease progression, and does not need to make treatment judgments after the IRC assessment results are available. Participants will enter the discontinuation follow-up period after permanent discontinuation.

The participant should enter the discontinuation follow-up period after discontinuation. The study doctor should conduct a telephone visit once a week to ask whether the participant has experienced adverse events or used other drugs or treatment methods. The time window for telephone visit is ±3 days. Participants should come to the hospital to receive the last treatment visit 28 days after the last treatment. If the participant experiences any adverse event or use any concomitant medication during this period, he/she must report it to the Investigator in a timely manner, and go to the hospital to receive necessary intervention if necessary. If the drug is permanently discontinued due to the occurrence of adverse events related to the investigational product, follow-up must be carried out until the outcome is reached.

Participants should come to the hospital for the last treatment visit within 28 + 7 days after the last treatment and complete the following examinations:

- - - Physical examination;
    - Vital signs;
    - Blood routine;
    - Blood biochemistry;
    - Urinalysis;
    - 12-lead ECG;
    - Echocardiography;
    - ECOG score;
    - Pregnancy test (for women of childbearing potential only);
    - Coagulation test;
    - Serum virology (Hepatitis B five-item test ± HBV DNA); hepatitis B five-item test will only be performed during the screening period; the Investigator will determine whether HBV DNA quantitative test is required;
    - Immunogenicity blood sample collection (only applicable to participants in the investigational product group);
    - Recovery of drugs (only applicable to participants in the control drug group) and patient diary cards.

Any information such as any adverse events and concomitant medications obtained during the discontinuation follow-up period must be recorded in the electronic case report form (eCRF).

#### Long-term follow-up period

After the discontinuation follow-up period, the participants will enter the long-term follow-up period to provide sufficient data to support the analysis and assessment of long-term benefit endpoints. During the long-term follow-up period, unless the participant discontinues the drug due to IRC-assessed disease progression or death, the participant must come to the hospital for an imaging efficacy assessment at the original scheduled time (every 6 weeks ± 3 days after randomization) until IRC-assessed disease progression or death or refusal to come to the hospital for follow-up or the end of the trial (including completion of the trial and early discontinuation of the trial), which mainly includes:

- - - Tumor imaging assessment:
      - Contrast-enhanced CT or MRI of chest, abdomen and pelvic cavity: Target lesions and non-target lesions should be selected according to the initial scan, and all subsequent scans should use the same method; for patients who are allergic to contrast agents, CT or MRI plain scan can be performed if determined by the Investigator;
      - Head MRI: To be performed at the discretion of the Investigator;
      - Bone emission computed tomography (ECT): To be performed after judged by the Investigator based on the participant’s specific condition (such as bone pain or elevated alkaline phosphatase);
      - The Investigator may decide whether to perform other imaging examinations, such as breast X-ray, breast ultrasound or other imaging examinations of suspected metastatic lesions, based on the signs and status of individual participants.
    - Anti-tumor efficacy assessments will be performed according to the RECIST 1.1 (Appendix 3).

When it is determined that the participant has disease progression or the participant refuses to come to the hospital for the relevant examinations, the Investigator will conduct a telephone visit every 3 months (based on 30 days/month) to learn whether the participant receives other anti-tumor treatments and the participant’s survival information, until the participant dies, withdraws informed consent, is lost to follow-up, refuses telephone follow-up or the trial ends (including completion of the trial and early discontinuation of the trial). The time window for telephone visit is ±7 days. Only serious adverse events related to the investigational product will be collected during the long-term follow-up period.

#### Unscheduled Visit

For the safety of the participants, the Investigator may ask the participants to undergo additional visits or examinations. Unscheduled visits or test results must be recorded in the eCRFs.

#### Population Pharmacokinetic Study

PK samples will be collected and tested in participants in the investigational product group to assess the population PK profile of ARX788 in patients with HER2-positive advanced breast cancer. If conditions permit, population PK samples should be collected and tested in all participants in the investigational product group. The specific sampling time is as follows:

- - - Within 0.5 h before the second dose of the investigational product, 1.5 h (± 30 min) after the completion of the dosing (after the completion of the ARX788 intravenous infusion), and 144 h (± 24 h) after the completion of the dosing (after the completion of the ARX788 intravenous infusion);
    - Within 0.5 h before the fourth dose of the investigational product, 1.5 h (± 30 min) after the completion of the dosing (after the completion of the ARX788 intravenous infusion), and 144 h (± 24 h) after the completion of the dosing (after the completion of the ARX788 intravenous infusion).

About 5 mL of blood sample will be collected at each blood collection point. For the collection, processing, storage and transportation of samples, see the relevant laboratory standard operating procedures or relevant clinical operation manual for details. The concentration of ARX788, total antibody and the metabolite pAF-AS269 of ARX788 in the blood samples will be tested at each time point.

### Study Evaluation

#### Efficacy Evaluation

The clinical trial inspection items related to efficacy evaluation in this trial are arranged as follows:

##### Anti-tumor Efficacy Evaluation

Imaging will be completed to document baseline tumor status for all participants during the screening period (to be completed within 28 days before the first dose of the investigational product, and as close to the start time of drug administration as possible). During the treatment period, the tumor efficacy will be evaluated every 6 weeks after randomization until the participant experiences disease progression or refuses to come to the hospital for follow-up or dies or the trial ends (including completion of the trial and early discontinuation of the trial), whichever occurs first. During the long-term follow-up period, unless the participant discontinues the drug due to IRC-assessed disease progression or death, the participant must come to the hospital for a tumor imaging efficacy assessment at the original scheduled time (every 6 weeks ± 3 days after randomization) until the participants develops the IRC-assessed disease progression or refuses to come to the hospital for follow-up or dies or the trial ends (including completion of the trial and early discontinuation of the trial), whichever occurs first. Related imaging examinations mainly include:

- - - - Contrast-enhanced CT or MRI of chest, abdomen and pelvic cavity: Target lesions and non-target lesions should be selected according to the initial scan, and all subsequent scans should use the same method; for patients who are allergic to contrast agents, CT or MRI plain scan can be performed if determined by the Investigator;
      - Head MRI: The head MRI is a mandatory examination during the screening period, and may be performed in the investigator’s judgment during the treatment period and long-term follow-up visit;
      - Bone emission computed tomography (ECT): ECT is mandatory during the screening period and is performed during the treatment period and long-term follow-up visit at the discretion of the Investigator based on the participant’s specific condition (e.g., presence of bone pain or elevated alkaline phosphatase);
      - The Investigator may decide whether to perform other imaging examinations, such as breast MRI, breast X-ray, breast ultrasound or other imaging examinations of suspected metastatic lesions, based on the signs and status of individual participants.

The Investigator and the IRC will perform anti-tumor efficacy assessments as per RECIST1.1 (Appendix 3), with the following requirements:

- - - - At least 1 measurable lesion at baseline;
      - The same assessment methods and assessment techniques must be used for lesion documentation at baseline and during the follow-up period;
      - For patients with PR or CR for the first time, efficacy must be confirmed after at least 4 weeks. Confirmation of efficacy can be carried out at planned tumor efficacy assessment every 6 weeks, as described in Appendix 3;
      - If the participant has received an unscheduled assessment and has no disease progression, every effort should be made to perform subsequent assessments at the scheduled time;
      - If emergency medical intervention is required for a participant with rapid tumor progression or symptomatic progression (e.g., metastases to central nervous system, respiratory failure caused by tumor compression, spinal cord compression), then the participant will no longer receive subsequent treatment;
      - The Investigator may decide whether the participant will continue to receive the study treatment based on the Investigator-assessed disease progression, and does not need to make treatment judgments after the IRC assessment results are available.

##### Telephone Visit

The participant should enter the discontinuation follow-up period after discontinuation. The study doctor should conduct a telephone visit once a week (time window of ± 3 days) to ask whether the participant has experienced adverse events or used other drugs or treatment methods. If the participant experiences any adverse event or use any concomitant medication during this period, he/she must report it to the Investigator in a timely manner, and go to the hospital to receive necessary intervention if necessary. During the long-term follow-up period, when it is determined that the participant has disease progression or refuses to come to the hospital for the relevant tests, the Investigator will conduct a telephone visit every 3 months (based on 30 days/month) (time window of ±7 days) to learn whether the participant receives other anti-tumor treatments and the participant’s survival information.

##### Efficacy Evaluation Indicators

**Primary Efficacy Evaluation Indicators**

Progression-free survival (PFS) based on the Independent Review Committee (IRC) assessment, defined as the time from randomization to IRC-assessed disease progression (PD) or death due to any reason, whichever occurs first.

**Secondary Efficacy Evaluation Indicators**

Overall survival (OS), defined as the time from randomization to death due to any reason.

Progression-free survival (PFS) based on the Investigator assessment, defined as the time from randomization to Investigator-assessed disease progression (PD) or death due to any reason, whichever occurs first;

Objective response rate (ORR, including PR and CR), defined as the proportion of patients with complete response or partial response. Anti-tumor efficacy will be evaluated as per RECIST1.1. For patients with PR or CR for the first time, efficacy must be confirmed after at least 4 weeks. Confirmation of efficacy can be carried out at planned tumor efficacy assessment every 6 weeks, as described in Appendix 3;

Disease control rate (DCR), defined as the proportion of patients who achieved response (PR+CR) and stable disease (SD) after treatment. For patients evaluated with SD, the efficacy needs to be evaluated as SD at least once after at least 12 weeks after randomization, as described in Appendix 3;

Duration of response (DOR), defined as the time to disease progression or death after the first evaluation as CR or PR.

#### Safety Evaluation Indicators

During the trial, participants need to undergo safety tests at the specified time, including laboratory tests (blood routine, urinalysis, blood biochemistry, etc.), vital signs, 12-lead electrocardiogram (ECG), and physical examination. The clinical manifestation, severity, occurrence time, end time, duration, treatment measures and outcome of any adverse event should be recorded, and its correlation with the investigational product should be determined. AEs are graded as per the NCI CTCAE V5.0 criteria, except for cardiac failure (graded as per the NYHA criteria [Appendix 5]). The correlation between any adverse event and the investigational product will be assessed by the Investigator as per the attribution evaluation criteria specified in the Protocol.

Safety evaluation indicators include but are not limited to the following:

- - 1. Any spontaneously reported and all directly observed adverse events;
    2. Any abnormal changes in vital signs and physical examination;
    3. Abnormal clinically significant laboratory findings, abnormal physical examination results and hematological examination results after treatment.

The arrangements and specific indicators of the safety evaluation-related clinical examination items are as follows. Refer to Appendix 1 Clinical Trial Flow Chart for details.

##### Vital Signs

Vital signs mainly include blood pressure, pulse, respiratory rate and body temperature. All participants in this study will undergo vital signs examination during the screening period (D-28 - D-1), within 30 min before the first dose of the investigational product and 2 h ± 30 min after the administration on C1D1, on C1D7 (± 1 day), on C1D14 (± 3 days), within 3 days before administration of the investigational product in C2 and each subsequent cycle, and at the last treatment visit. Participants in the investigational product group will also undergo a vital sign test 2 h ± 30 min after the administration of the investigational product in each cycle.

Systolic blood pressure and diastolic blood pressure are measured after the participant rests quietly for at least 5 minutes. Standard equipment will be used for all recordings, and either automated or manual measurements are acceptable, but the same method should be used for all assessments of individual participants during the trial.

##### Physical Examination

In this trial, physical examinations should include breast, apart from routine skin, lymph nodes, head and neck, chest, abdomen, spine and limbs items. Physical examinations will be performed during the screening period (D-28 - D-1), on C1D1 (before the first dose of the investigational product), and within 3 days before the administration of the investigational product in C2 and each subsequent cycle, and at the last treatment visit.

During the screening period, we will determine whether the participants meet the enrollment criteria in conjunction with the physical examination results and record their medical history after the Investigator’s determination; during the treatment and follow-up periods, we will determine whether the participants have adverse events in conjunction with the physical examination results and record the adverse events after the Investigator’s determination.

##### Weight Measurement

The participants will be measured for weight during the screening period (D-28 - D-1), on C1D1 (before the first dose of the investigational product), and within 3 days before the administration of the investigational product in C2 and each subsequent cycle. These data will be recorded in the original records (weight measurement data during the screening period will be included in the demographic data). If the weight or body surface area change from the last measurement or baseline is >± 10% (whichever is greater), the specific dose of ARX788 (as per the weight) or capecitabine (as per the body surface area) should be recalculated, and the weight or body surface area at the new dose should be used as the baseline for subsequent calculations.

##### 12-Lead ECG

All participants in this study will undergo the 12-lead ECG examination on D-28 to D-1 (the examination will be performed within 1 week before administration during the screening period, otherwise it will need to be performed again within 1 week before administration), before the first dose of the investigational product (test results within 48 hours before the start of administration are acceptable) and 2 h ± 30 min after the administration on C1D1, on C1D7 (± 1 day), on C1D14 (± 3 days), within 3 days before administration of the investigational product in C2 and each subsequent cycle, and at the last treatment visit. Participants in the investigational product group will also undergo the 12-lead ECG examination 2 h ± 30 min after the administration of the investigational product in each cycle.

ECG indicators mainly include heart rate, PR interval, QRS duration, QT interval and QTc interval.

During the screening period, we will determine whether the participants meet the enrollment criteria in conjunction with the 12-lead ECG results and record their medical history after the Investigator’s determination; during the treatment and follow-up periods, we will determine whether the participants have adverse events in conjunction with the 12-lead ECG results and record the adverse events after the Investigator’s determination.

##### Echocardiography

Echocardiography will be performed during the screening period (D-28 - D-1), within 3 days before administration of the investigational product in C3 and every 2 cycles thereafter, and at the last treatment visit.

##### ECOG Score

ECOG scoring will be performed during the screening period (D-28 - D-1), within 3 days before administration of the investigational product in C3 and every 2 cycles thereafter, and at the last treatment visit.

##### Routine Laboratory Tests

Routine laboratory tests include blood routine, blood biochemistry, urinalysis and coagulation.

Blood biochemistry and blood routine will be performed on D-28 to D-1 (the tests will be performed within 1 week before administration during the screening period, otherwise they will need to be performed again within 1 week before administration), on C1D7 (± 1 day), on C1D14 (± 3 days), within 3 days before administration of the investigational product in C2 and each subsequent cycle, and at the last treatment visit.

Urinalysis will be performed on D-28 to D-1 (the test will be performed within 1 week before administration during the screening period, otherwise it will need to be performed again within 1 week before administration), within 3 days before administration of the investigational product in C2 and each subsequent cycle, and at the last treatment visit.

The coagulation test will be performed on D-28 to D-1, within 3 days before administration of the investigational product in C3 and every 2 cycles thereafter, and at the last treatment visit.

The indicators of routine laboratory tests are described below.

- - - - Blood routine includes: White blood cells, absolute lymphocyte count, absolute neutrophil count, absolute monocyte count, absolute eosinophil count, absolute basophil count, neutrophil percentage, lymphocyte percentage, red blood cells, hemoglobin, hematocrit, mean corpuscular volume, mean corpuscular hemoglobin, and platelets;
      - Urinalysis includes: Specific gravity, PH, bilirubin, urine protein, glucose, ketone body, urobilinogen, nitrite, urine occult blood (occult blood), red blood cells and white blood cells;
      - Blood biochemistry includes: Lactate dehydrogenase (LDH), glutamic-oxaloacetic transferase, alanine aminotransferase, r-glutamyl transpeptidase, creatine kinase, alkaline phosphatase, total bilirubin, direct bilirubin, indirect bilirubin, total protein, albumin, globulin, urea (urea nitrogen), creatinine, uric acid, sodium, potassium, chloride, calcium, magnesium and glucose;
      - Coagulation includes: Prothrombin time, activated partial thromboplastin time, thrombin time and international normalized ratio (INR).

##### Serum Virology Testing

Serum virology will be performed from D-28 to D-1 with indicators including HBsAg, HBsAb, HBeAg, HBcAb, HBeAb, HBV DNA quantification (only performed in participants with positive hepatitis B surface antigen), HCV antibody test, HIV antibody test, and treponema pallidum test. During the treatment period, the Hepatitis B five-item test will be performed only in participants who are positive for hepatitis B surface antigen during the screening period within 3 days before administration of the investigational product in C3 and every 2 cycles thereafter, and at the last treatment visit, and the Investigator will determine whether the HBV DNA quantitative test is required.

##### Pregnancy Test

The pregnancy test will be performed during the screening period (D-28 - D-1), within 3 days before administration of the investigational product in C3 and every 2 cycles thereafter, and at the last treatment visit.

#### Population Pharmacokinetic Evaluation

PK samples will be collected from participants in the investigational product group for testing before and after the second and fourth dosing of the investigational product to evaluate the population PK characteristics of ARX788 in patients with HER2-positive advanced breast cancer. If conditions permit, population PK samples should be collected and tested in all participants in the investigational product group.

#### Immunogenicity Evaluation

Anti-drug antibodies in the participants before and after treatment may affect the efficacy and safety of ARX788. Therefore, blood samples will be collected from participants in the investigational product group for immunogenicity testing before the first dose of the investigational product, every 2 cycles after the first dose of the investigational product (visit time window of ±3 days), and at the last visit, and the effect of the production of the antibodies on safety and efficacy will be analyzed.

All samples will be analyzed for anti-drug antibodies (ADAs) during the screening test. Study samples with results below the screening threshold will be reported as ADA negative. If a positive result in the screening test is present, the sample will be further analyzed by a confirmatory test. All samples determined positive will be reported as ADA positive and will be analyzed for the presence of neutralizing antibodies (NAbs). The incidence of ADAs will be summarized based on the immunogenicity analysis set. If applicable, the effect of ADAs on the efficacy and safety of ARX788 will be evaluated.

#### Other Assessment

##### Demographic Information

Demographic data will be collected from D-28 to D-1, including name, age, date of birth, gender, height, weight, ID card number, ethnicity and address. These data will be recorded in the original records, and data that do not involve the personal information or privacy of participants will be recorded in the CRFs.

##### Medical History

Medical history refers to past or concomitant diseases (or signs and symptoms, if the diagnosis cannot be determined) that occurred before signing the informed consent form, which may include: past medical history (tumor, cardiovascular disorder, infectious disease, severe drug allergy, etc.), history of alcoholism, history of drug abuse, and present medical history. The medical history should be included in the original records and CRFs during the screening period. Participants with specific previous or current diseases are not allowed to participate in this clinical trial (see Inclusion and Exclusion Criteria in Sections 4.1 and 4.2).

##### Previous Medications and Concomitant Medications and Treatments

Previous medications and treatments include but are not limited to the following:

- - - 1. Cumulative amount of previous anthracycline use;
      2. Use of capecitabine within 6 months before the first dose of the investigational product;
      3. Radiation, surgery or steroid therapy within 4 weeks prior to the first dose of the investigational product to control symptoms of brain metastasis;
      4. Use of chemotherapy, radiotherapy and immunotherapy within 4 weeks prior to the first dose of the investigational product;
      5. Use of breast cancer endocrine therapy and palliative radiotherapy for bone metastases within 2 weeks before the first dose of the investigational product.

Concomitant medications and treatments refer to two or more drugs/treatments administered simultaneously, defined in the trial as any drug/treatment given simultaneously/concurrently with the investigational products (all trial drugs in the treatment cycle) during the treatment period to treat any event until the completion of the examinations associated with the final treatment visit.

At each visit during the trial, from the initial screening to the end-of-trial examination, the Investigator will need to ask the participants for information about any medications/treatments used since the last visit. Any previous medications and concomitant medications/treatments will be recorded in the original records and CRFs, and all records include the following information: name of drug/treatment, route of administration/treatment, start time, stop time, reason for use, etc. If the reason for use of concomitant treatment meets the definition of AE, the relevant information should also be recorded in the participant’s original record and CRF.

### Adverse Events

#### Definition of Adverse Events

Adverse events is any untoward medical occurrence in a clinical trial participant following the use of investigational product, which can be manifested as symptoms/signs, diseases or laboratory test abnormalities, but a clear causal relationship with the investigational product cannot necessarily be inferred. Symptoms/signs of the participants that already existed during the screening period, if not worsening after entering the trial, will not be reported as adverse events. However, once they worsen during the trial, they should be recorded as adverse events. Expected disease progression and signs, symptoms, laboratory findings related to disease progression are not considered as AEs, unless disease progression is more frequent or more severe than expected (as determined by the Investigator and/or pharmacovigilance staff) or disease progression leads to death. Adverse events include but are not limited to the following:

- - - Adverse events that are routinely observed and adverse events that are foreseeable by the pharmacological effects of the investigational product.
    - Laboratory abnormalities (including blood biochemistry, blood routine, and urinalysis results), vital signs, or physical examination findings that the Investigator considers to be clinically significant.
    - Accidental injury, reason for adjusting medication (drug and/or dose), any reason for medical treatment, care or pharmacological consultation, and reason for hospitalization or surgery.
    - Overdose and medication errors, regardless of clinical effects.

**Pre-dose adverse events:**

A pre-dose adverse event refers to an adverse event that occurs after the participant signs the informed consent form and before the start of use of the investigational product, or that is a pre-existing medical condition that worsens after the signing of the informed consent form and before the start of use of the investigational product. (Note: Pre-existing abnormalities identified by assessments and examinations performed during screening are not adverse events but are recorded as medical history).

**Post-dose adverse events:**

An adverse event that occurs after drug administration refers to an adverse event that occurs during the period from the start of use of the investigational product to the last visit, or an adverse event that occurs before drug administration or an aggravation of pre-existing medical condition.

#### Monitoring of Adverse Events

Participants should be closely monitored for adverse events during the trial. The duration of monitoring for adverse events is defined as the period from the participant’s screening (the date of first signing of the informed consent form) until the end of the last treatment (28+7 days after the last treatment) visit. The serious adverse events related to the investigational product during the long-term follow-up period will be monitored.

Sources of adverse events include, but are not limited to:

- - - The participant’s answers to questions about his/her health status (non-leading questions at each visit such as “How have you been feeling since your last visit?”);
    - Symptoms spontaneously reported by the participant;
    - Changes or abnormal findings or tests that are assessed by the Investigator as clinically significant;
    - Other information related to the participant’s health that is made known to the Investigator (e.g., hospitalization).

#### Recording of Adverse Events

All adverse events will be coded and described using the Medical Dictionary for Regulatory Activities (MedDRA, V22.0 or above). During the trial, the adverse event record form should be truthfully completed to record the name, date of occurrence, severity, causal relationship with the investigational product, medical treatment, measures taken on the investigational product, duration, whether it is a serious adverse event (SAE), and outcome status.

If possible, adverse events should be recorded as per the exact diagnosis. If an AE cannot be recorded as an exact diagnosis, separate signs and symptoms should be recorded. Each diagnosis/symptom should be recorded separately.

If the same adverse event occurs more than once in the participant and the participant has recovered between the occurrences, the adverse event should be recorded separately according to the number of occurrences. If the severity of the adverse event changes during its occurrence, it should be re-recorded as a separate AE.

Attention: The measures taken are not adverse events; the reasons for the measures are adverse events. Hospitalization is not an adverse event; the cause of the hospitalization is an adverse event. Death is not an adverse event; the cause of death is an adverse event (except for unexplained sudden death, which should be recorded as an adverse event).

The start date of an adverse event is the date the first sign or symptom is first observed. If the adverse event is an abnormal laboratory test or test result with clinical significance, the start time is the sampling date.

#### Abnormal Laboratory Findings

If an abnormal laboratory finding meets any of the following criteria, it should be reported as an adverse event:

1. The test result is related to concomitant symptoms;
2. The test result requires other diagnostic tests or treatment measures/surgical intervention;
3. The test result results in a change in the participant’s investigational product dose or discontinuation of the test, the need for concomitant medication, or other treatment;
4. The Investigator or Sponsor believes that the test result should be reported as an adverse event.

A test that is solely for the purpose of reviewing an abnormality and does not meet any of the above conditions does not constitute an adverse event. Any abnormal test result determined to be error is not required to be reported as an adverse event.

#### Severity of Adverse Events

The investigators will categorize the severity of each adverse event by describing the clinical symptoms, referring to the five-grade criteria developed as per NCI CTCAE V5.0, as follows:

- Grade 1: Mild, asymptomatic or mild symptoms; clinical or diagnostic findings only; intervention not indicated.
- Grade 2: Moderate; minimal, local or noninvasive intervention indicated; limiting age-appropriate instrumental activities of daily living (ADL)^*^;
- Grade 3: Severe or medically significant but not immediately life-threatening; hospitalization or prolongation of hospitalization indicated; disabling; limiting self care ADL^**^;
- Grade 4: Life-threatening consequences; urgent intervention indicated;
- Grade 5: Death related to AE.

*Instrumental ADL refer to preparing meals, shopping for groceries or clothes, using the telephone, managing money, etc.

**Self care ADL refer to bathing, dressing and undressing, feeding self, using the toilet, taking medications, and not bedridden.

#### Adverse Event Causality Assessment

The correlation between all AEs that occur during the clinical trial and the investigational product must be determined. The following factors need to be comprehensively considered in the analysis of the correlation between an AE and the investigational product:

1. Whether there is a reasonable time sequence between the AE and the investigational product;
2. Whether the AE disappears or alleviates after the investigational product is discontinued or reduced in dosage;
3. Whether the AE recurs or worsens after the investigational product is re-administered;
4. Whether the clinical symptoms of the AE are consistent with the known pharmacological and toxicological knowledge of the investigational product;
5. Whether the AE can be explained by the participant’s clinical status, psychological factors, concomitant medications or treatments, and other environmental factors.

The Investigator should assess the possible correlation between adverse events and the investigational product and concomitant drugs with reference to the following five-grade classification criteria:

1. Certainly related: The occurrence time of the AE is consistent with the chronological order of administration, and the AE is consistent with the type of known AEs of the investigational product, improves after discontinuation, and reappears after repeated administration;
2. Probably related: The occurrence time of the AE is consistent with the chronological order of treatment, the AE is consistent with the known principle of the treatment, and cannot be explained by the participant’s clinical status, psychological factors, concomitant medication or treatment, other environmental factors, etc., and the AE improves after discontinuation;
3. Possibly related: The occurrence time of the AE is consistent with the chronological order of administration, the AE is consistent with the known type of AEs of the investigational product, and the participant’s clinical status, psychological factors, concomitant medication or treatment, and other environmental factors may also cause the AE;
4. Unlikely related: The occurrence time of the AE is consistent with the chronological order of administration, the AE is less consistent with the known type of AEs of the investigational product, and the participant’s clinical status, psychological factors, concomitant medication or treatment, and other environmental factors may also cause the AE;
5. Not related: The occurrence time of the AE is not consistent with the chronological order of administration, the AE is not consistent with the known type of AEs of the investigational product, and the participant’s clinical status, psychological factors, concomitant medication or treatment, and other environmental factors may also cause the AE; the AE resolves after improvement in disease status or discontinuation of other treatment modalities, and the AE occurs with repeated use of other treatments.

#### Suspected Unexpected Serious Adverse Reactions

The nature and severity of the clinical manifestations exceed the suspected and unexpected serious adverse reactions outlined in the Investigator’s Brochure of the investigational product, the package insert of the marketed drugs, or the summary of product characteristics, or other available data.

#### Definition of Serious Adverse Events

A serious adverse event (SAE) refers to an adverse medical event that results in death, is life-threatening, causes permanent or severe disability or loss of function, requires hospitalization or prolongation of hospitalization, or leads to congenital anomaly or birth defect after the participant receives the investigational product:

1. Resulting in death;
2. Life-threatening;
3. Resulting in significant or permanent disability or loss of function;
4. Leading to hospitalization or prolongation of hospitalization;
5. Congenital anomaly or birth defects;

The following conditions are not considered hospitalizations or do not need to be reported as SAEs: emergency room visit; hospital stay for observation within 24 hours; hospitalization for routine examination (hospital stay less than 24 hours); hospitalization for social reasons (e.g., hospitalization due to unattended care); hospitalization due to surgery on the date agreed before the trial; if the participant has already suffered from a disease and the disease has not worsened during the trial, the planned hospitalization and/or surgical treatment before the trial is performed is not an adverse event.

1. Other important medical events that may lead to the above conditions if not treated;
2. New lesions in malignant tumors or disease progression and their corresponding signs and symptoms should not be reported as SAEs, unless disease progression is more frequent or more severe than expected (as determined by the Investigator and/or pharmacovigilance staff) or disease progression results in death.

#### Safety Reporting

The Investigator shall immediately report all serious adverse events to the Sponsor in writing, except for serious adverse events that do not require immediate reporting as specified in this Protocol or other documents (e.g., Investigator’s Brochure) (e.g., 8.8 Definition of Serious Adverse Events), and shall provide a timely, detailed, written follow-up report thereafter. The Investigator is also required to make the necessary written reports in accordance with the notification requirements and timelines for serious adverse events by the local regulatory authority and its Ethics Committee.

The Investigator must fill out the serious adverse event report form, describe in detail the time of occurrence, seriousness criteria, duration, relationship with the investigational product, measures taken, outcome, etc. of the serious adverse event, and sign the report. If there is follow-up information, it should be reported in a timely manner. The initial report should include, to the extent possible, the following: source of report, basic information of participant, name of investigational product, name of serious adverse event, duration, seriousness criteria, correlation with investigational product, treatment and outcome of the event. The serious adverse event report and follow-up report should indicate the participant’s identification code in the clinical trial, rather than the participant’s real name, ID card number, address and other identity information. Adverse events and laboratory abnormalities that are important to safety evaluation as specified in the Protocol should be reported to the Sponsor in accordance with the requirements and time limit specified in the Protocol. For reports involving death events, the Investigator should provide the Sponsor and the Ethics Committee with other required information, such as autopsy reports and final medical reports.

After receiving the relevant safety information of the clinical trial provided by the Sponsor, the Investigator should sign and read it in a timely manner, consider whether the treatment of the participant is adjusted accordingly, communicate with the participant as soon as possible when necessary, and report to the Ethics Committee suspected unexpected serious adverse reactions provided by the Sponsor.

The SAE reporting contact is shown in Table 4.

Table 5 Sponsor and mailing address for SAE reporting

| **Reported to** | **Contact Person** | **Contact Information** | **Mailing Address** |
| --- | --- | --- | --- |
| NovoCodex Biopharmaceuticals Co., Ltd. | XIONG Gaozhun | 13735219471  [xionggaozhun@zmc-china.com](mailto:xionggaozhun@zmc-china.com) | 5/F, North, Building 2, No. 398 Mahuan Road, Binhai New City, Shaoxing, Zhejiang Province |

In addition to the Sponsor, the Investigator should also report the death SAE to the Ethics Committee. During the same time period as the initial report, the Investigator/reporter must respond to all requests for follow-up information, such as: additional information, event results, final evaluation, special records, and, if necessary, answers to any questions raised by the Sponsor/designated party with regard to the AE.

Note: The safety reporting requirements shall conform to the Good Clinical Practice, effective July 1, 2020, under the No. 57 Announcement of 2020 issued by the National Medical Products Administration/National Health Commission. In addition, if information of the Sponsor’s contact person to whom SAEs are reported changes, the Sponsor should inform the Investigator in a timely manner and does not need to revise the Protocol.

#### Adverse Events of Special Interest

Adverse events of special interest include the following hepatotoxicity, hematological toxicity, pulmonary toxicity, ocular toxicity and infusion-related reactions associated with ARX788, including:

- Hepatotoxicity: Grade 3 and above elevated transaminase (AST/ALT) and Grade 2 and above elevated bilirubin;
- Hematologic toxicity: Grade 2 or higher thrombocytopenia;
- Pulmonary toxicity: Including but not limited to non-infectious pneumonia or interstitial pneumonia;
- Ocular toxicity: Including but not limited to dry eye or blurred vision, etc.;
- Infusion-related reactions: Including but not limited to fever, chills, stiffness, sweating or headache.

#### Pregnancy Reporting

To ensure the safety of participants, the Investigator or other site staff must report to the Sponsor each pregnancy event and pregnancy outcome that occurs in a participant (or participant’s partner) treated with the investigational product within 24 hours of learning of the pregnancy event using the appropriate pregnancy reporting form. An abnormal pregnancy outcome should be considered a serious adverse event and reported using the SAE reporting form and as per the SAE reporting process within 24 hours of discovery and knowledge of the event.

Pregnancy itself is not considered an adverse event unless it is suspected that the investigational product under trial may affect the effectiveness of the contraceptive pills. Congenital anomalies/birth defects and spontaneous abortions should be reported and treated as serious adverse events. Elective abortions without complications are not treated as adverse events. All pregnancy outcomes (spontaneous abortion, elective abortion, ectopic pregnancy, normal birth or congenital anomaly) should be followed up and recorded, even if the participant has withdrawn from the trial.

#### Follow-up of Adverse Events

Before the end of the last treatment visit, the Investigator must collect the information of each adverse event that occurred in the participant and follow up until the outcome.

After the participant’s last treatment visit, the Investigator must follow up on serious adverse events that occur during the trial or adverse events that are possibly, probably, and certainly related to the investigational product until they recover or the participant’s condition is stable or lost to follow-up. All relevant follow-up information must be reported to NovoCodex Biopharmaceuticals Co., Ltd.

### Risk Management Plan

The safety of ARX788 has been preliminarily explored in two human trials, and to a certain extent, the possible risks during treatment have been exposed.

**First-in-human Trial**

The first-in-human trial was an intravenous infusion of ARX788 monotherapy for HER2-positive advanced breast cancer in dose escalation in participants with advanced breast cancer who tested FISH+ or IHC 3+ for HER2. The dose groups that have conducted the trial include 0.33 mg/kg (1 participant), 0.66 mg/kg (1 participant), 1.3 mg/kg (3 participants), 2.20 mg/kg (3 participants) and 2.9 mg/kg (1 participant). The treatment-emergent adverse events (TEAEs) were summarized as follows.

A total of 139 TEAEs occurred in 9 participants, most of which (91 TEAEs) were mild. The most common TEAEs reported as per the preferred terms were nausea (6 participants [66.7%]), upper respiratory tract infection (4 participants [44.4%]), vomiting (4 participants [44.4%]), dry eye (4 participants [44.4%]), fatigue (4 participants [44.4%]) and alopecia (4 participants [44.4%]). Stomatitis occurred in one participant (11.1%) and was identified as DLT; disseminated intravascular coagulation occurred in one participant (11.1%) and was identified as an adverse event of special interest (AESI).

A total of 47 TEAEs were considered treatment related. The most common treatment-related AEs (TRAEs) reported as per the preferred terms were nausea (4 participants [44.4%]), alopecia (4 participants [44.4%]), hazy vision (3 participants [33.3%]), pneumonitis (3 participants [33.3%]) and fatigue (3 participants [33.3%]). The most common TRAEs reported as per the system organ class (SOC) were gastrointestinal disorders (6 participants [66.7%]), eye disorders (4 participants [44.4%]), respiratory, thoracic and mediastinal disorders (4 participants [44.4%]), skin and subcutaneous tissue disorders (4 participants [44.4%]), general diseases and administration site reactions (3 participants [33.3%]), vascular disorders (3 participants [33.3%]), and metabolism and nutrition disorders (2 participants [22.2%]). Among them, the TRAEs that occurred in at least 2 participants are described by the SOC and preferred terms as follows:

Table 6 Treatment-related AEs in ≥ 2 participants by system organ class and preferred terms

| **SOC/PT** | **Statistics** | **0.33**  **mg/kg**  **(N=1)** | **0.66**  **mg/kg**  **(N=1)** | **1.3 mg/kg**  **(N=3)** | **2.2 mg/kg**  **(N=3)** | **2.9 mg/kg**  **(N=1)** | **Total**  **(N=9)** |
| --- | --- | --- | --- | --- | --- | --- | --- |
| Any drug-related AE | n (%)  [nAE] | 1 (100.0%) [3] | 1 (100.0%) [3] | 3 (100.0%) [10] | 3 (100.0%) [21] | 1 (100.0%) [10] | 9 (100.0%) [47] |
| Gastrointestinal disorders | n (%)  [nAE] | 1 (100.0%) [1] | 1 (100.0%) [3] | 1 (33.3%) [1] | 2 (66.7%) [2] | 1 (100.0%) [6] | 6 (66.7%) [13] |
| Eye disorders | n (%)  [nAE] | 0 | 0 | 0 | 3 (100.0%) [5] | 1 (100.0%) [3] | 4 (44.4%) [8] |
| Vision dim | n (%)  [nAE] | 0 | 0 | 0 | 2 (66.7%) [2] | 1 (100.0%) [1] | 3 (33.3%) [3] |
| Respiratory, thoracic and mediastinal disorders | n (%)  [nAE] | 1 (100.0%) [1] | 0 | 1 (33.3%) [1] | 2 (66.7%) [8] | 0 | 4 (44.4%) [10] |
| Pneumonitis | n (%)  [nAE] | 0 | 0 | 1 (33.3%) [1] | 2 (66.7%) [6] | 0 | 3 (33.3%) [7] |
| Skin and subcutaneous tissue disorders | n (%)  [nAE] | 0 | 0 | 2 (66.7%) [4] | 2 (66.7%) [3] | 0 | 4 (44.4%) [7] |
| Alopecia | n (%)  [nAE] | 0 | 0 | 2 (66.7%) [4] | 2 (66.7%) [3] | 0 | 4 (44.4%) [7] |
| General and administration site discomfort | n (%)  [nAE] | 0 | 0 | 1 (33.3%) [2] | 2 (66.7%) [2] | 0 | 3 (33.3%) [4] |
| Fatigue | n (%)  [nAE] | 0 | 0 | 1 (33.3%) [2] | 2 (66.7%) [2] | 0 | 3 (33.3%) [3] |

**First-in-human Trial in China**

Another trial of ARX788 was conducted to evaluate the safety, tolerability and pharmacokinetic profile of ARX788 monotherapy for the treatment of HER2-positive advanced breast cancer in China, in which HER2 test results should be IHC 3+ and/or FISH+. As of January 20, 2020, a total of 51 participants entered the safety analysis set: 0.33 mg/kg Q3W (3 participants); 0.66 mg/kg Q3W (3 participants); 0.88 mg/kg Q3W (3 participants); 1.1 mg/kg Q3W (8 participants); 1.3 mg/kg Q3W (12 participants); 0.88 mg/kg Q4W (4 participants); 1.1 mg/kg Q4W (3 participants); 1.3 mg/kg Q4W (4 participants); and 1.5 mg/kg Q3W (11 participants).

A total of 51 (100%) participants reported treatment-emergent adverse events (TEAEs), and 49 (96.1%) participants experienced TEAEs related to the investigational product, of which most of the participants (45 participants, 91.8%) had the highest severity of Grade 2, and 11 participants (22.4%) had the highest severity of Grade 1; a small number of participants (4 participants, 8.2%) had severity of Grade 3-4, of which 3 participants (6.1%) with Grade 3 and 1 participant (2.0%) with Grade 4.

The most common system organ classes of the TEAEs related to the study included: investigations (47 participants, 92.2%), eye disorders (26 participants, 51%), general disorders and administration site conditions (22 participants, 43.1%), metabolism and nutrition disorders (22 participants, 43.1%), gastrointestinal disorders (21 participants, 41.2%), respiratory, thoracic and mediastinal disorders (19 participants, 39.2%), and skin and subcutaneous tissue disorders (18 participants, 35.3%).

The most common preferred terms of the TEAEs related to the study included: aspartate aminotransferase increased (35 participants, 68.2%), Alanine aminotransferase increased (26 participants, 51.0%), hypokalemia (16 participants, 31.4%), asthenia (16 participants, 31.4%), alopecia (16 participants, 31.4%), dry eye (15 participants, 29.4%), adrenocorticotropic hormone abnormal (15 participants, 29.4%), neutrophils decreased (12 participants, 23.5%), plasma aldosterone increased (12 participants, 23.5%), and dry mouth (12 participants, 23.5%).

As of January 20, 2020, a total of 11 (21.6%) participants had ≥ Grade 3 TEAEs, including: infectious pneumonia that occurred in 3 participants, and osteomyelitis, weight increased, neutrophil count decreased, hyponatremia, back pain, epistaxis, shock and electrocardiogram QTc interval prolongation that occurred in one participant each. Grade 3 or higher events that the Investigator determined to be related to the investigational product included: Grade 3 hyponatremia in 1 participant, Grade 4 neutrophil count decreased in 1 participant, Grade 3 electrocardiogram QTc interval prolonged in 1 participant, and Grade 3 pneumonia in 1 participant.

No participants in this phase I trial experienced DLT.

Six (12.5%) participants experienced treatment-emergent SAEs, which were shock, osteomyelitis and epistaxis, respectively in 1 participant, and 3 participants developed infectious pneumonia, among which only 1 participant (1.5 mg/kg Q3W group) experienced the treatment-emergent SAE related to the investigational product.

The details of SAEs during the treatment period are shown in the table below.

Table 7 SAEs summarized by MedDRA system organ class and preferred terms

| **MedDRA system organ class/preferred terms n(%)E** | **0.33(Q3) N=3** | **0.66(Q3) N=3** | **0.88(Q3) N=3** | **0.88(Q4) N=4** | **1.1(Q3) N=8** | **1.1(Q4) N=3 n(%)** | **1.3(Q3) N=12 n(%)** | **1.3(Q4) N=4 n(%)E** | **1.5(Q3) N=11 n(%)E** | **Total N=51 n(%)E** |
| --- | --- | --- | --- | --- | --- | --- | --- | --- | --- | --- |
| **(SAE)** | 0 | 1(33.3)1 | 1(33.3)1 | 0 | 1(12.5)1 | 0 | 0 | 2(50.0)2 | 1(9.1)1 | 6(11.8 )6 |
| **Infections and infestations** | 0 | 0 | 1(33.3)1 | 0 | 0 | 0 | 0 | 2(50.0)2 | 1(9.1)1 | 4( 7.8 )4 |
| **Infectious pneumonia** | 0 | 0 | 0 | 0 | 0 | 0 | 0 | 2(50.0)2 | 1(9.1)1 | 3( 5.9 )3 |
| **Osteomyelitis** | 0 | 0 | 1(33.3)1 | 0 | 0 | 0 | 0 | 0 | 0 | 1( 2.0 )1 |
| **Respiratory, thoracic and mediastinal disorders** | 0 | 0 | 0 | 0 | 1(12.5)1 | 0 | 0 | 0 | 0 | 1( 2.0 )1 |
| **Epistaxis** | 0 | 0 | 0 | 0 | 1(12.5)1 | 0 | 0 | 0 | 0 | 1( 2.0 )1 |
| **Vascular and lymphatic disorders** | 0 | 1(33.3)1 | 0 | 0 | 0 | 0 | 0 | 0 | 0 | 1( 2.0 )1 |
| **Shock** | 0 | 1(33.3)1 | 0 | 0 | 0 | 0 | 0 | 0 | 0 | 1( 2.0 )1 |

As of January 20, 2020, 32 (62.7%) participants experienced treatment-related adverse events of special interest (AESIs), including ocular toxicity in 27 participants (52.9%), hepatotoxicity in 8 participants (17.6%), pulmonary toxicity in 9 participants (17.6%), and hematological toxicity in 1 participant (2.0%). The adverse events of special interest (AESIs) are summarized by category of special interest and preferred terms as follows:

Table 8 Adverse events of special interest (AESIs) in the first-in-human trial in China

| **Preferred term of AESI** | **0.33(Q3) N=3** | **0.66(Q3) N=3** | **0.88(Q3) N=3** | **0.88(Q4) N=4** | **1.1(Q3) N=8** | **1.1(Q4) N=3** | **1.3(Q3) N=12** | **1.3(Q4) N=4** | **1.5(Q3) N=11** | **Total N=51** |  |
| --- | --- | --- | --- | --- | --- | --- | --- | --- | --- | --- | --- |
| **AESI** | 0 | 1(33.3)1 | 1(33.3)1 | 2(50.0)5 | 5(62.5)11 | 2(66.7)3 | 9(75.0)24 | 3( 75.0)10 | 4(36.4.0)7 | 27(52.9)62 |  |
| **Ocular toxicity** | 0 | 0 | 1(33.3)1 | 1(25.0)1 | 5(62.5)8 | 0 | 8(66.70)19 | 3( 75.0)4 | 3(27.3)5 | 21(41.2)39 |  |
| **Dry eye** | 0 | 0 | 0 | 0 | 4(50.0)4 | 0 | 4(50.0)4 | 2(50.0)2 | 5(45.5)6 | 15(29.4)16 |  |
| **Blurred vision** | 0 | 0 | 0 | 0 | 1(12.5)1 | 0 | 4(33.3)4 | 0 | 4(36.4)4 | 9(19.6)9 | |
| **Diplopia** | 0 | 0 | 0 | 1(25.0)1 | 0 | 0 | 0 | 0 | 0 | 1( 2.0)1 | |
| **Corneal injury** | 0 | 0 | 0 | 0 | 0 | 0 | 3( 25.0)3 | 0 | 2(18.2)2 | 5( 9.8)5 | |
| **Keratitis** | 0 | 0 | 0 | 0 | 0 | 0 | 2( 3.9)2 | 0 | 0 | 2( 3.9)2 | |
| **Conjunctival hyperemia** | 0 | 0 | 0 | 0 | 1(12.5)1 | 0 | 0 | 0 | 0 | 1( 2.0)1 | |
| **Conjunctivitis** | 0 | 0 | 1(33.3)1 | 0 | 0 | 0 | 0 | 0 | 0 | 1( 2.0)1 | |
| **Increased eye discharge** | 0 | 0 | 0 | 0 | 0 | 0 | 1( 8.3)1 | 0 | 0 | 1( 2.0)1 | |
| **Ocular discomfort** | 0 | 0 | 0 | 0 | 1(12.5)1 | 0 |  | 0 | 0 | 1( 2.0)1 | |
| **Eye pain** | 0 | 0 | 0 | 0 | 1(12.5)1 | 0 | 1( 8.3)1 |  | 0 | 2( 2.0)2 | |
| **Dacryorrhea** |  |  |  |  |  |  |  | 1( 25.0)1 | 1( 9.1)1 | 2( 2.0)2 | |
| **Hepatotoxicity** | 0 | 1(33.3)1 | 0 | 2(50.0)3 | 1(12.5)1 | 1(33.3)2 | 1( 8.3)1 | 1( 25.0)1 | 1( 9.1)1 | 8(15.7)10 | |
| **AST increased** | 0 | 1(33.3)1 | 0 | 2(50.0)3 | 1(12.5)1 | 1(33.3)1 | 0 | 1( 25.0)1 | 1( 9.1)1 | 7(13.7)8 | |
| **ALT increased** | 0 | 0 | 0 | 0 | 0 | 1(33.3)1 | 0 | 0 | 0 | 1( 2.0)1 | |
| **Blood bilirubin increased** | 0 | 0 | 0 | 0 | 0 | 0 | 1( 8.3)1 | 0 | 0 | 1( 2.0)1 | |
| **Pulmonary toxicity** | 0 | 0 | 0 | 1(25.0)2 | 1(12.5)1 | 1(33.3)1 | 3( 25.0)3 | 1(25.0)3 | 2(18.1)2 | 9(17.6)12 | |
| **Pneumonitis** | 0 | 0 | 0 | 1(25.0)2 | 1(12.5)2 | 1(33.3)1 | 2( 25.0)2 | 1(25.0)2 | 2(18.2)2 | 10(19.6)12 | |
| **Interstitial lung disease** | 0 | 0 | 0 | 0 | 0 | 0 | 1( 8.3)1 | 1(25.0)1 | 0 | 2( 3.9)2 | |
| **Hematologic toxicity** | 0 | 0 | 0 | 0 | 0 | 0 | 0 | 1(25.0)1 | 0 | 1( 2.0)1 | |
| **Platelet count decreased** | 0 | 0 | 0 | 0 | 0 | 0 | 0 | 1(25.0)1 | 0 | 1( 2.0)1 | |

The investigational product ARX788 and the marketed drug T-DM1 are HER2 therapeutic drugs with the same mechanism, both of which are conjugated and synthesized with small molecule substances of anti-HER2 antibody-conjugated toxins. It is expected that the safety data results obtained from previous T-DM1 clinical trials have certain reference significance for the risk control of ARX788 clinical trials.

In the clinical trial of T-DM1 for second-line treatment of metastatic breast cancer, the most common (incidence > 25%) adverse reactions in 490 HER2-positive metastatic breast cancer participants receiving T-DM1 treatment were nausea, fatigue, musculoskeletal pain, hemorrhage, thrombocytopenia, transaminases increased, headache and constipation. The most common NCI-CTCAEV3.0 ≥ Grade 3 adverse reactions (frequency > 2%) were thrombocytopenia, transaminases increased, anemia, hypokalemia, peripheral neuropathy and fatigue. The specific details of adverse events that occurred in ≥ 10% of participants are as follows:

**Blood and lymphatic system**

- Thrombocytopenia: (31%, 15% with Grades 3-4)
- Anemia: (14%, 4.1% with Grades 3-4)

**Gastrointestinal disorders**

- Nausea: (40%, 0.8% with Grades 3-4)
- Constipation: (27%, 0.4% with Grades 3-4)
- Diarrhea: (24%, 1.6% with Grades 3-4)
- Vomiting: (19%, 0.8% with Grades 3-4)
- Abdominal pain: (19%, 0.8% with Grades 3-4)
- Dry mouth: (17%, 0% with Grades 3-4)
- Stomatitis: (14%, 0.2% with Grades 3-4)

**General diseases and infusion reactions**

- Fatigue: (36%, 2.5% with Grades 3-4)
- Fever: (19%, 0.2% with Grades 3-4)
- Weakness: (18%, 0.4% with Grades 3-4)

**Test items**

- Elevated transaminase: (29%, 8.0% with Grades 3-4)

**Metabolism and nutrition disorders**

- Hypokalemia: (10%, 2.7% with Grades 3-4)

**Musculoskeletal and connective tissue disorders**

- Musculoskeletal pain: (36%, 1.8% with Grades 3-4)
- Arthralgia: (19%, 0.6% with Grades 3-4)
- Myalgia: (14%, 0.6% with Grades 3-4)

**Nervous system disorders**

- Headache: (28%, 0.8% with Grades 3-4)
- Peripheral neuropathy: (21%, 2.2% with Grades 3-4)
- Vertigo: (10%, 0.4% with Grades 3-4)

**Mental disorders**

- Insomnia: (12%, 0.4% with Grades 3-4)

**Respiratory, thoracic and mediastinal disorders**

- Nasal bleeding: (23%, 0.2% with Grades 3-4)
- Cough: (18%, 0.2% with Grades 3-4)
- Dyspnea: (12%, 0.8% with Grades 3-4)

**Skin and subcutaneous tissue disorders**

- Rash: (12%, 0% with Grades 3-4)

**Vascular disorders**

- Hemorrhage: (32%, 1.8% with Grades 3-4)

In addition to the above adverse events, clinically relevant adverse events reported in < 10% of participants also included: dyspepsia (9%), urinary tract infection (9%), chills (8%), dysgeusia (8%), neutrophils reduced (7%), peripheral edema (7%), pruritus (6%), hypertension (5%), elevated alkaline phosphatase (4.7%), Hazy vision (4.5%), conjunctivitis (3.9%), dry eye (3.9%), lacrimation increased (3.3%), drug allergy (2.2%), left ventricular insufficiency (1.8%), infusion related reaction (1.4%), pneumonitis (1.2%), nodular hyperplasia (0.4%), and portal hypertension (0.4%).

Considering the above information together, participants participating in this trial may be at risk for one or more of the adverse events including, but not limited to, those described above. In this trial, if a participant experiences a drug-related adverse event, the administration should be withheld in a timely manner as required by the Protocol and intervention should be given in a timely manner in accordance with clinical routine treatment measures. If interstitial pneumonia and ocular toxicity related to the investigational product occur, they can be treated with reference to the relevant event handling procedures.

The trial site must be equipped with necessary medical rescue equipment, first-aid drugs and emergency measures. An emergency medical event response team should be established when necessary to handle medical emergencies and accidental injuries in accordance with the relevant standard operating procedures. The possible adverse events, especially unexpected adverse events, should be closely observed, analyzed and communicated in a timely manner, and the adverse event observation log should be completed. The site should establish a contact procedure with the hospital intensive care units for participant transfer and care, and establish communication and exchange between the Investigator and the laboratory and Sponsor to ensure that possible adverse events are communicated and handled in a timely manner.

The inclusion/exclusion criteria specify participants’ organ functions (including liver, kidney, hematopoiesis, cardiac ejection function, etc.), and exclude participants with cardiac disorder, eye disorder, pulmonary disorder and other severe and unmanageable systemic diseases to avoid disorder aggravation of such participants in the trial. During the treatment period, at least one safety test will be performed every cycle, mainly including: vital signs, physical examination, 12-lead ECG, blood routine and blood biochemistry (including liver function, kidney function, electrolytes, etc.). Electrocardiogram, echocardiogram, coagulation and ECOG tests will be performed at the end of the treatment period. Through the above examinations and the Investigator’s observation, adverse events can be detected as early as possible, and the necessary intervention treatment is given to fully guarantee the safety of the participants. During the follow-up period, if the participant experiences any adverse event or use any concomitant medication during this period, he/she must report it to the Investigator in a timely manner, and go to the hospital to receive necessary intervention if necessary. If the drug is discontinued due to the occurrence of adverse events related to the investigational product, follow-up is required until the outcome.

### Statistical Analysis

Statistical analysis will be performed using SAS 9.4 or later. Continuous variables will be summarized using descriptive statistics, including number of participants, mean, median, standard deviation, maximum and minimum values. Categorical variable will be described by the number and percentage of participants in each category. Unless otherwise specified, the hypothesis test will use a two-sided test at α = 0.05.

#### Statistical Hypotheses and Sample Size Calculation

This trial intends to enroll 440 participants with HER2-positive locally advanced or metastatic breast cancer, and assign the participants randomly to either the investigational product group or the control drug group in a 1:1 ratio, using the number of prior chemotherapy lines (0-1, >1) received for recurrent or metastatic lesions and whether they have concomitant visceral metastases (yes, no) as stratification factors.

In the key phase III clinical trial^[14]^ of T-DM1, the median PFS of patients receiving lapatinib combined with capecitabine in the control drug group was 6.4 months; in the key phase II clinical trial^[17]^ of pyrotinib maleate, the median PFS of patients receiving lapatinib combined with capecitabine in the control drug group was 5.6 months.

In combination with the above data, the efficacy of the study group will be compared with that of the control drug group using the IRC-assessed PFS as the primary endpoint in this trial. Assuming that the median PFS of the control drug group is 6.4 months, HR = 0.7, and the two-sided test level is 0.05, two interim analyses will be performed when 160 participants complete the Cycle 4 visit and the number of PFS events reaches 2/3 (see the Interim Analysis section for details) using the O’Brien Fleming Type I error spending function. Under these hypotheses, 335 IRC-assessed PFS events are required to gain a statistical power of 90%. Assuming 24 months of enrollment and an annual dropout rate of 5%, if we want to reach the target number of events 8 months after the last participant is enrolled, we approximately need a sample size of 220 participants/group, with a total of 440 participants in both groups.

#### Interim Analysis

In this trial, the primary analysis of PFS will be conducted when 335 PFS events assessed by the Independent Review Committee (IRC) occur. Two interim analyses will be planned during the trial. The interim analysis will be performed by the Independent Data Monitoring Committee (IDMC). Details will be specified in the IDMC Charter.

- - - The first interim analysis will be performed at the completion of the Cycle 4 visit in 160 participants (approximately 80 in each group), and the difference in point estimate of ORR between the two groups at that time will be calculated. If this difference is less than 2% during the interim analysis, a futility judgment should be considered to decide whether to continue the enrollment. Whether to continue the enrollment will be decided by the IDMC in combination with other safety and efficacy data at that time. Considering that in the key phase III clinical trial of T-DM1, the difference in the best ORR between the two groups was 12.7% (95% two-sided confidence interval (6.0%, 19.4%)), if the difference between the two groups in this trial is similar to that in the key phase III clinical trial of T-DM1, there is approximately a 10% probability that the two-group difference will be less than 2%. No test of superiority will be performed in this interim analysis, but for conservative reasons, Lan-DeMets α-spending function to approximate O'Brien-Fleming will be used. Conservatively, considering that the correlation coefficient between ORR and PFS will be 1 then (the actual correlation coefficient should be far less than 1), the efficacy boundaries will be estimated based on the actual number of PFS events. Under the same hypotheses as the calculation of sample size, the first interim analysis is expected at about 12 months. The number of events is approximately 74 (22%). In this case, the efficacy boundary will be two-sided α=0.0002 at the first interim analysis.
    - The second interim analysis will be performed when 224 (2/3) IRC-assessed PFS events occur, and the superiority test will be performed and the sample size will be re-estimated. Using Lan-DeMets α-spending function to approximate O'Brien-Fleming, the efficacy boundary will be two-sided α=0.0123 at the second interim analysis. The conditional power will be calculated based on the hazard ratio (HR) trend at that time. When the conditional power is not high enough (specific boundaries are specified in the IDMC Charter, roughly 60%-80%), the sample size will be re-estimated.
    - If the sample size is re-estimated at the second interim analysis, the hypothesis test statistics for the final analysis will be adjusted according to the method of Wassmer (2006)^[25]^ and Cui, Hung, Wang (1999)^[26]^. As per Lan-DeMets α-spending function to approximate O'Brien-Fleming, the efficacy boundary will be two-sided α=0.0462 during the final analysis. If the final number of events or the number of events in the first and second interim analyses is slightly different from the plan (possibly due to the fact that multiple events occur on the same day when the number of events is reached), the efficacy boundaries will be adjusted accordingly based on the actual number of events.

#### Statistical Analysis Population

##### Intent-to-Treat (ITT) Analysis Set

All randomized participants will be included in the ITT analysis set. The ITT analysis set will be used for the analysis of participant distribution, demographic and baseline characteristics, and will also be used as the primary analysis set for all efficacy endpoints.

##### Per-Protocol Set (PPS)

All participants who are randomized and treated with at least one dose of investigational product or control drug without major protocol violations constitute the per-protocol set for this trial. The PPS will be used for the supporting analysis of the primary efficacy endpoints.

##### Safety Analysis Set (SS)

All participants who are randomized and treated with at least one dose of investigational product or control drug and perform at least one post-treatment safety evaluation are included in the SS. The SS is the safety evaluation population of this trial.

##### Population PK Analysis Set

All participants who receive at least one dose of the investigational product treatment and have at least one post-dose PK data are included in the population PK analysis set.

##### Immunogenicity Analysis Set

All participants who receive at least one dose of the investigational product treatment and have at least one post-dose immunogenicity evaluation data are included in the immunogenicity analysis set.

#### Statistical Analysis Methods

##### Demographic Data and Other Baseline Characteristics

Demographic data and other baseline characteristics will be tabulated, summarized and descriptively analyzed using descriptive statistics.

##### Efficacy Analysis

The primary endpoint of this trial is the IRC-assessed PFS, and the primary analysis will be performed in the ITT population.

The primary hypothesis test will first be performed on the difference in the IRC-assessed PFS between the treatment groups. If the IRC-assessed PFS is statistically different between the treatment groups, a hypothesis test will be further performed on the OS of the treatment groups at a two-tailed level of 0.05.

All time-to-event endpoints (such as PFS, DOR, OS, etc.) will be summarized and estimated using the Kaplan-Meier method. Survival functions of the two treatment groups will be compared using the Log-rank test stratified by randomization stratification factors. A Cox proportional risk model will also be used to calculate the hazard ratio (HR) between the two treatment groups and their corresponding confidence intervals using the treatment group and randomization stratification factors (the number of prior chemotherapy lines (0-1, >1) received for recurrent or metastatic lesions and whether they have concomitant visceral metastases (yes, no)) as independent variables.

The ORR of the investigational product group and the control drug group and its 95% confidence interval (estimated by the Clopper-Pearson method) will be calculated, respectively. The difference in ORR between the treatment groups and its 95% confidence interval will be calculated. The stratified Cochran-Mantel-Haenszel (CMH) method adjusted by the randomization stratification factors will be used to calculate the P values of the treatment groups. DCR will be analyzed using a statistical method similar to that for ORR.

##### Safety Analysis

Adverse events will be coded using the Medical Dictionary for Regulatory Activities (MedDRA). They will be analyzed based on treatment emergent adverse events (TEAEs). TEAE is defined as an adverse event that occurs or worsens (of the adverse event that occurs before medication or the pre-existing medical conditions) during the period from the start of use of the investigational product to the last visit. TEAEs, TEAEs related to the investigational product, and serious adverse events (SAEs) in both groups will be summarized and analyzed by system organ class (SOC) and preferred term (PT), and the number of participants and incidence rates will be calculated.

Descriptive statistics will be used to summarize vital signs, physical examination, ECG and laboratory tests as well as their changes from baseline. Changes in laboratory tests relative to baseline will be described using a shift table.

##### Population Pharmacokinetic Analysis

PK data will be summarized using descriptive statistics (sample size, mean, geometric mean, standard deviation, minimum, median, maximum, coefficient of variation), if applicable. An appropriate model will be selected for PK analysis, if applicable, and the specific method is detailed in the independent statistical analysis plan.

##### Immunogenicity Analysis

The incidence of ADA will be summarized by visit. For ADA-positive participants, descriptive analysis will also be performed on the Nabs test results. If applicable, the effect of ADAs on the efficacy and safety of ARX788 will be evaluated.

### Data Processing

#### Raw Data and Original Documents

**Raw data**

Raw data are all information in the original records and certified copies of the original records generated through clinical findings, observations, or other activities in the trial that are necessary for the restoration and evaluation of the trial. The original data are included in the original records.

**Original records**

Original records are the earliest documents, data and records (e.g., hospital records, clinical and office charts, laboratory records, memoranda, participant diaries or evaluation forms, drug dispensing records, data recorded by automated instruments, certified copies or duplicates that have been verified as accurate copies, microfiche, photographic negatives, microfilm or magnetic media, X-rays, participant files, and records kept by pharmacy, laboratory and pharmaceutical technology departments participating in the clinical trial).

**Definition of Clinical Trial Specific Raw Data**

The Investigator must keep the participant records.

The participant’s medical history, physical examination results and other clinically relevant results, demographic data and AEs will be recorded in the specific original records and then transcribed into the case report form (CRF) of each participant. Laboratory results will be transcribed into the CRF by the Investigator. The results of the 12-lead ECG evaluation will be transcribed into specific parts of the CRF and marked as normal, abnormal without clinical significance or abnormal with clinical significance. If necessary, specific abnormalities will also be described in detail in the CRF.

**CRF**

This trial uses an electronic case report form, and the content is to be completed by the Investigator or his/her authorized personnel through the clinical electronic data capture and management system (EDC). Before the start of the trial, the CRF will be set up in the EDC system and an account will be assigned to the site’s Investigator and/or his/her authorized who is responsible for completing the CRF form.

- - 1. For all participants who have signed the informed consent form, any items on the case report form must be carefully and thoroughly documented in accordance with the instructions for completing the case report form;
    2. All data in the case report form must be checked against the participant’s original document data for accuracy;
    3. Data that are significantly high or outside the clinically acceptable range are participant to verification and, if needed, to the judgment of the Investigator;
    4. For additional details, please refer to the guidelines for completing the electronic case report form.

After the completed electronic case report form is reviewed by the clinical monitor, the data manager performs data verification and management. After the data collection and cleaning are completed, the Investigator will sign and confirm online.

The original documents of the participants are the original records of the participants kept at the site. The trial data must be entered into the CRF in a timely manner.

The completed CRF is the exclusive property of the Sponsor and cannot be provided to a third party in any form without the written permission of the Sponsor, except to authorized representative of the relevant regulatory authority or Ethics Committee (EC) (see Section 14).

#### Data Management

- - 1. This trial uses an electronic data management system. Creation of electronic case report form: The data manager will create the electronic case report form (eCRF) based on the Protocol.
    2. Permission assignment: The data manager will create accounts and grant different permissions based on the different identities of the entry clerks, investigators, clinical monitors, etc. Data entry clerks have data entry, modification and query feedback permissions, investigators have modification, browsing, query feedback and review permissions, monitors have browsing and query sending permissions, and data managers have browsing, query sending and data lock permissions.
    3. Data entry: The clinical Investigator or the data entry clerk (clinical coordinator) designated by the Investigator will promptly and accurately enter the data in the study medical records into the eCRF.
    4. Sending and resolving of queries: The monitor and data manager will send all queries through the eCRF, and the entry clerk or the Investigator will answer the queries and modify the wrong data. If necessary, the queries may be sent repeatedly, and all records will be kept in the eCRF.
    5. Modification and review of data: The data entry clerk or the Investigator may modify the data after verification, and the reason for modification needs to be filled in on the eCRF. The Investigator has review access to all final data.
    6. Data locking and export: After all data are reviewed to be correct, the data manager will lock the data. Any modification after data locking will require signatures of the Sponsor, Investigator, entry clerk, monitor and data manager before implementation. All data are finally exported by the data manager and submitted to the statistician for analysis.

#### Independent Data Monitoring Committee

The Independent Data Monitoring Committee (IDMC) (Data and Safety Monitoring Board) is an independent committee established by the Sponsor. This committee will regularly evaluate the efficacy and safety data of the clinical trial. The IDMC should have written operating procedures and keep all of its meeting minutes. The IDMC Charter will be developed separately.

#### Independent Endpoint Review Committee

The primary endpoint (PFS) of this trial will be evaluated based on the evaluation results of the Independent Review Committee (IRC). The IRC is an independent committee composed of experts who are responsible for evaluating the results of the adjudication of endpoint events using uniform criteria as defined by the Protocol. The IRC can generate more reliable data relative to the Investigator’s individual evaluation of the events in the multicenter trial. The purpose is to ensure proper assessment using uniform criteria and to avoid deviations arising from different standards of event assessment from site to site. The IRC members of this trial are composed of members who have certain experience in the participant area of this clinical trial. Any member must be independent of the Investigator and Sponsor and cannot directly participate in the implementation of the trial, and must have no conflict of interest that may affect their impartiality and independent decision-making. The IRC Charter will be separately formulated.

### Clinical Monitoring

To ensure the rights and interests of the participants in the trial, the data accuracy and completeness of the trial records and reports, and the trial complies with the approved Protocol and relevant laws and regulations, the Sponsor should appoint trained monitors with sufficient scientific and clinical knowledge required for clinical trial monitoring and acceptable qualifications to monitor the clinical trial. Duties of monitors:

1. The monitor should be familiar with the knowledge related to the investigational product, the content of the Protocol, the informed consent form and other written materials provided to the participants, as well as the basic clinical trial SOP and other relevant regulations.
2. The monitor should conscientiously perform the monitoring duties in the clinical trial in accordance with the requirements of the Sponsor, and ensure that each medical institution can implement the Protocol and record the clinical trial data correctly.
3. The monitor serves as the primary contact person between the Sponsor and the Investigator. Before the trial, the monitor should confirm that the Investigator has sufficient qualifications and resources to complete the trial, and that the medical institution has the appropriate conditions for completing the trial, including staffing and training, complete laboratory equipment, good operation and various trial-related conditions for tests.
4. The monitor should verify that the storage time and storage conditions of the investigational products throughout the trial are acceptable and sufficient; that the investigational products are only provided to eligible participants according to the dose stipulated in the Protocol; that the participants receive instructions on the correct use, handling, storage and return of the investigational products; that the receipt, use and return of the investigational products by each trial medical institution are properly controlled and recorded; and that the disposal of the unused investigational products by each trial medical institution is compliant with the relevant regulations and the requirements of the Sponsor.
5. The monitor should understand the implementation status of the Protocol by the Investigator during the clinical trial; confirm that all participants have signed the written informed consent form before the trial; ensure that the Investigator receives the latest version of the Investigator’s Brochure, all trial-related documents, and all necessary supplies for the trial, and implements them in accordance with regulatory requirements; and ensure that the Investigator and all staff participating in the trial fully understand the trial.
6. The monitor should verify that the Investigator and all staff participating in the trial perform the respective duties stipulated in the Protocol and written contract, and do not delegate these duties to unauthorized persons; understand the enrollment rate of participants and the progress of the trial, confirm the eligibility of enrolled participants, and report the enrollment rate and progress of the trial; confirm that all data records and reports are correct and complete, and that the trial records and documents are updated and kept in good condition in real time; and verify that all medical reports, records and documents provided by the Investigator are accurate, complete, timely, legible, dated and numbered (with trial number).
7. The monitor should verify the accuracy and completeness of the CRF entries and compare them with the original documents. The monitor should particularly verify that the data specified in the Protocol are accurately recorded on the CRF and consistent with the original documents; that the dose change, treatment change, adverse events, concomitant medications, intercurrent diseases, loss to follow-up, missed tests, etc. of each participant should be confirmed and recorded; that the follow-up, tests and examinations not performed by the Investigator should be clearly and truthfully documented, and whether corrections are made to errors and omissions; and that the withdrawal and loss to follow-up of enrolled participants are recorded and described in the CRF.
8. The monitor should inform the Investigator of any incorrect completion, omission or unclear writing of the CRF; and ensure that the corrections, additions or deletions made are performed by the Investigator or trial personnel authorized by the Investigator to modify the CRF, and are signed and dated by the person making the modification, with an explanation of the reason for modification. This authorization shall be recorded in writing.
9. The monitor should ensure that all adverse events are reported within the prescribed time limit in accordance with the basic specifications, the Protocol, and the requirements of the Ethics Committee, the Sponsor and the drug regulatory authority.
10. The monitor should determine whether the Investigator has kept the essential documents in accordance with the basic specifications.
11. The monitor who identifies deviations from the Protocols SOP, GCP, and relevant regulatory requirements should communicate with the Investigator in a timely manner and take appropriate measures to prevent recurrence of the deviations.
12. After monitoring at each visit, the monitor must make a written report and send it to the Sponsor. The report should specify the date and place of monitoring, the name of the monitor, the name of the Investigator and other staff contacted by the monitor, and the problems found during monitoring; the summary of the monitoring work, the clinical trial problems and facts, deviations from and defects in the Protocol, and the monitoring conclusion; the corrective measures taken or to be taken to address the problems found in monitoring, and the recommendations implemented to ensure compliance with the Protocol; and the sufficient details to check compliance with the monitoring plan.
13. The monitor should promptly provide the monitoring results to the Sponsor, including the Sponsor’s management, the lead of the clinical trial, and the project supervision and management personnel. The Sponsor shall review and follow up on the problems mentioned in the monitoring report, and prepare a document for retention.

### Quality Assurance and Control

1. Quality control measures in the laboratory: The laboratory should establish standard operating procedures and quality control procedures. Dedicated personnel must be responsible for special testing items. See the laboratory operation manual and quality control manual for details.
2. The investigators participating in the clinical trial must have professional expertise, qualifications and competencies for clinical trials, be vetted for qualifications, and be relatively fixed.
3. Before the start of the trial, the trial participants will receive training on the Protocol, current GCP and relevant standard operating procedures of this trial, so that the study staff have a full understanding of the specific contents of the Protocol and its indicators.
4. To ensure the quality of the clinical trial and ensure the rights and interests of the participants, the clinical site has set up a special project team, and the Sponsor has appointed one monitor to monitor the progress of the trial.

### Ethical Standards and Informed Consent

#### Review and Approval of Ethics Committee

The Sponsor shall obtain the approval of the Ethics Committee before conducting the clinical trial. The Sponsor should obtain the approval documents and other relevant materials reviewed by the Ethics Committee, including: name and address of the Ethics Committee; members of the Ethics Committee participating in the project review; statement that the review process meets the requirements of the basic specifications and relevant laws and regulations; the Ethics Committee’s approval of the implementation of the clinical trial and listing of the reviewed documents, such as the latest Protocol, the written informed consent form of the participant, the written documents provided to the participant, the participant enrollment procedures, the relevant documents for the payment of the participant, and other documents required for review by the Ethics Committee. If the review comment of the Ethics Committee is “approved after making necessary amendments”, to modify the Protocol, informed consent form, and documents provided to the participant and/or other relevant documents, the Sponsor should negotiate with the Investigator and the medical institution in which he/she works to modify the relevant documents and submit them to the Ethics Committee. If the review comment of the Ethics Committee is “not approved”, the Sponsor and the Investigator shall, after modifying the issues related to the clinical trial, submit them to the Ethics Committee for re-review.

#### Informed Consent of Participants

- - 1. During the participant informed consent process, the Investigator must comply with the regulatory requirements of the drug regulatory authority, as well as the ethical principles of this Protocol and the Declaration of Helsinki. The Investigator may start the clinical trial only after the informed consent form and other information provided to the participants have been approved by the Ethics Committee.
    2. During the course of the clinical trial, when the Investigator obtains new information that may affect the participant’s continued participation in the trial, the participant or his/her legally acceptable representative should be promptly informed in writing such as informed consent form. After all relevant new information that needs to be informed to the participant is approved by the Ethics Committee, the participant should sign the informed consent form again. Newly enrolled participants should sign an updated informed consent form and other written materials.
    3. Neither the Investigator nor any other study staff may use coercion, inducement, or other improper means to influence participants to participate in, or continue to participate in, the clinical trial.
    4. Any written or verbal information related to the trial must not contain any language that would cause the participant and his or her legally acceptable representative to waive his or her legal rights or that would release the Investigator and his or her medical institution, the Sponsor and its agent from liability.
    5. The Investigator or designated study staff should fully inform the participants of all matters related to the clinical trial, including written information and approval comments from the Ethics Committee. Participants who are incapable of expressing informed consent should have their legally acceptable representatives give informed consent on their behalf.
    6. Both oral and written information provided to the participant, such as informed consent form, should be in language and expressions that are easily understood by the participant or his or her legally acceptable representative.
    7. Before implementing the informed consent process, the Investigator or designated study staff should give the participant or his/her legally acceptable representative sufficient time and opportunity to understand the details of the trial, and answer all trial-related questions raised by the participant or his/her legally acceptable representative in detail.
    8. The participant or his/her legally acceptable representative, as well as the Investigator who executes the informed consent, should sign and date the informed consent form separately.
    9. During the informed consent process, if the participant or his/her legally acceptable representative lacks reading ability, an impartial witness is required to assist and witness the informed consent process. The Investigator should explain the contents of the informed consent form and other written materials to the participant or his/her legally acceptable representative and witness. If the participant or his/her legally acceptable representative verbally agrees to participate in the trial and signs the informed consent form, the witness must also sign and date the informed consent form to prove that the participant or his/her legally acceptable representative has obtained an accurate explanation from the Investigator about the informed consent form and other written materials, understood the relevant content, and agreed to participate in the clinical trial.
    10. The participant or his/her legally acceptable representative should receive a copy of the signed and dated informed consent form and other written materials provided to the participant. During the trial, the participant or his/her legally acceptable representative should receive a copy of the signed and dated updated informed consent form, as well as the revised text of other written materials.
    11. When a legally acceptable representative gives informed consent on behalf of a participant, the participant should be informed and helped to understand as much information about the clinical trial as possible, and the participant should sign and date the informed consent form in person whenever possible. When the participant participates in a non-therapeutic trial and there is no expected clinical benefit, the participant must sign and date the informed consent form.
    12. In urgent cases, when informed consent cannot be obtained from the participant prior to participation in the clinical trial, the consent of the participant’s legally acceptable representative must be obtained. If the participant is unable to give prior informed consent and if his or her legally acceptable representative is not present, the participant’s mode of enrollment should be clearly stated in the Protocol and/or other documents and approved in writing by the Ethics Committee; informed consent should be obtained as soon as possible from the participant or his or her legal representative to continue to participate in the clinical trial.

### Changes in Trial Procedures

#### Amendment to the Study Protocol

Any changes to this Protocol will be recorded in the Protocol amendment and agreed by the Investigator and Sponsor prior to implementation.

EC approval is required for any amendments that may affect the safety of participants or require changes to the trial scope/design, such as an increase in the dose or exposure time of the investigational product, an increase in the number of participants, or addition of new tests or procedures.

However, changes to the Protocol to eliminate direct harm to trial participants may be implemented before EC approval. Afterwards, it is necessary to immediately submit the Protocol amendment to the EC for approval in accordance with regulatory requirements, and indicate the reason for doing so.

#### Deviation from the Study Protocol

In the event of a deviation from the Protocol, the Investigator must notify the monitor and must review and discuss the impact of the deviation. Any deviation from the Protocol must be recorded in the protocol deviation log/report. The protocol deviation log/report will be kept by NovoCodex Biopharmaceuticals Co., Ltd. The protocol deviation log/report and supporting documentation must be retained in the Investigator’s file and project core file.

#### Early Discontinuation of the Trial

The Investigator and NovoCodex Biopharmaceuticals Co., Ltd. will reserve the right to discontinue the trial at any time. If it is necessary to terminate the trial, a consensus on the discontinuation steps will be reached after consultation between both parties. At the discontinuation of the trial, NovoCodex Biopharmaceuticals Co., Ltd. and the Investigator will ensure that due consideration will be given to protecting the best interests of the participants. The regulatory authority and the Ethics Committee will be notified.

In addition, NovoCodex Biopharmaceuticals Co., Ltd. will reserve the right to terminate the study site’s participation in the trial.

### Data and Information Storage

#### Confidentiality and Ownership of Trial Data

Any confidential information related to the investigational products or this trial, including any data and results derived from this trial, is the exclusive property of NovoCodex Biopharmaceuticals Co., Ltd. The Investigator and any other personnel participating in this trial should protect the confidentiality of the proprietary information belonging to NovoCodex Biopharmaceuticals Co., Ltd.

#### Data Publication and Public Disclosure

##### Publication Policy

At the end of the trial, the Investigator may work with NovoCodex Biopharmaceuticals Co., Ltd. to write one or more articles for co-publication, with the Investigator as first author and corresponding author.

The copyright is determined in accordance with the standards of the International Committee of Medical Journal Editors (ICMJE). The total number of authors is determined based on the guidelines of relevant journals or conferences. If there is any disagreement in the contents of the publication, the views of the Investigator and NovoCodex Biopharmaceuticals Co., Ltd. will be fairly and fully reflected in the publication.

Any external Contract Research Organization (CRO) or laboratory that participates in this trial does not have the publication right of this trial.

If the Investigator wishes to independently publish/introduce any results of the trial, the draft of the manuscript/publication must be submitted in writing to NovoCodex Biopharmaceuticals Co., Ltd. for the company’s comments prior to submission. This statement, apart from restricting the disclosure of the intellectual property rights of NovoCodex Biopharmaceuticals Co., Ltd., does not grant NovoCodex Biopharmaceuticals Co., Ltd. any rights to edit the published content. If NovoCodex Biopharmaceuticals Co., Ltd. considers the contents of a publication to be patentable, publication in a scientific publication is not permitted until the submitted patent application is published. In this case, the Investigator may decide to revise or delay the publication, so that NovoCodex Biopharmaceuticals Co., Ltd. has sufficient time to seek patent protection for the invention.

##### Public Disclosure Policy

ICMJE member magazines have adopted the trial registration policy as a condition for publication. This policy requires all clinical trials to be registered on a clinical trial registry website. Therefore, NovoCodex Biopharmaceuticals Co., Ltd. will be responsible for registering this trial on the appropriate public registry network (i.e., <http://www.chinadrugtrials.org.cn/> as designated by the NMPA).

#### Study Data Retention

To ensure the evaluation and supervision of the clinical trial by the National Health Commission and the Sponsor, the Investigator should agree to retain all study data, including the original records of participant hospitalization, informed consent forms, case report forms, and detailed records of drug dispensing. The clinical trial essential documents should be kept until 2 years after the investigational product is approved for marketing or 5 years after the discontinuation of the clinical trial. All data from this clinical trial belong to NovoCodex Biopharmaceuticals Co., Ltd. The Investigator shall not provide them to third parties in any form without the written consent of the Sponsor, except as required by the National Health Commission.

The clinical trial essential documents refer to separate, integrated, quality-controlled documents that evaluate the implementation of the clinical trial and the data generated. These documents are used to prove that the Investigator, Sponsor and monitor have complied with this Protocol and the laws and regulations related to drug clinical trials during the trial. The Sponsor, and the Investigator and the medical institution in which he/she works should confirm that both parties have the premises and conditions for retaining these clinical trial essential documents. The equipment for keeping documents should be able to prevent direct light, be waterproof, have an environment conducive to long-term document preservation, and regulations and standard operating procedures (SOPs) for document management should be established. The documents being retained need to be easy to identify, locate, retrieve and return to their original location.

If some documents generated during the clinical trial are not listed in the essential document management directory established at each stage of the clinical trial, the Sponsor, Investigator and study institution must also include them in their respective essential document archives. The Sponsor should ensure that the Investigator can retain the case report form (CRF) data submitted to the Sponsor. Copies used as source documents must meet the requirements for certified copies. The Investigator and the medical institution in which he/she works are able to manage all clinical trial essential documents and ensure the authenticity of raw data generated during the entire implementation of the clinical trial.

At the start of the clinical trial, the Investigator and the medical institution in which he/she works, and the Sponsor shall establish the dossier management of trial essential documents in their respective offices. At the end of the trial, the monitor must review and confirm the essential documents of the Investigator, study institution and Sponsor, and these documents must be properly stored in their respective clinical trial dossiers.

### Responsibilities of the Parties and Other Relevant Provisions

1. **Sponsor**

In accordance with GCP regulations, the Sponsor should conscientiously perform the following duties:

- - Responsible for initiating, applying for, organizing, monitoring and auditing a clinical trial.
  - Select the Institution and Investigator of the clinical trial, and recognize their qualifications and conditions to ensure the completion of the trial.
  - Provide the Investigator’s Brochure, which includes chemical, pharmaceutical, toxicological, pharmacological and clinical information and data of the investigational product.
  - Organize the clinical trial as per the Protocol only after obtaining the approval document from the Ethics Committee.
  - Work together with the Investigator to design the Protocol, and clarify the responsibilities and tasks in such areas as Protocol implementation, data management, statistical analysis, result reporting, and paper publication. Sign the Protocol and Contract agreed by both parties.
  - Provide the Investigator with the investigational product that is easily identifiable, correctly coded, and affixed with special labels, and ensure acceptable quality. The investigational products should be properly packaged and stored as per the Protocol. The Sponsor shall establish a management system and record system for the investigational products.
  - Appoint a qualified monitor acceptable to the Investigator.
  - Establish a quality control and quality assurance system for the clinical trial, and organize audits of the clinical trial to ensure quality.
  - Upon receipt of safety-related information from any source, the Sponsor should immediately analyze and evaluate it, including severity, correlation to the investigational product, and whether it is an anticipated event. The Sponsor should quickly report suspected unexpected serious adverse reactions to the Investigator, clinical trial institution and Ethics Committee; the Sponsor should also report these reactions to the drug regulatory authority and health authority.
  - Notify the Investigator, the Ethics Committee and the National Medical Products Administration and state the reason before suspending a clinical trial.
  - Provide insurance coverage for participants participating in the clinical trial and cover the cost of treatment and corresponding financial compensation for participants who suffer trial-related damage or death. The Sponsor should provide legal and financial guarantees to the Investigator, except in cases caused by medical malpractice.
  - When the Investigator fails to conduct the clinical trial as per the approved Protocol or relevant regulations, the Sponsor should point out the situation for correction. If the situation is serious or persists, the Sponsor should terminate the Investigator’s participation in the clinical trial and report the situation to the drug regulatory authority.

1. **Study Site and Investigator**

In accordance with GCP regulations, the Investigator should conscientiously perform the following duties:

- - Read and understand the content of the Protocol in detail and strictly implement the Protocol.
  - Understand and be familiar with the nature, effect, efficacy and safety of the investigational product (including relevant data from preclinical studies of the drug), and master all new information related to the drug discovered during the clinical trial.
  - The Investigator must conduct the clinical trial in a medical institution with good medical facilities, laboratory equipment and staffing. The institution should have all facilities to handle emergencies to ensure the safety of participants. Laboratory findings should be accurate and reliable.
  - Obtain the consent from the medical institution or the competent authority and ensure sufficient time to lead and complete the clinical trial within the time limit stipulated in the Protocol. The investigator must explain the materials, regulations and responsibilities related to the trial to all staff participating in the clinical trial, and ensure that a sufficient number of participants who meet the Protocol enter the clinical trial.
  - The Investigator should explain the details of the trial approved by the Ethics Committee to the participants and obtain informed consent forms.
  - Be responsible for making medical decisions related to the clinical trial, and ensure that the participants receive appropriate treatment when they experience adverse events during the trial.
  - Take necessary measures to protect the safety of the participants, and record them on file. If a serious adverse event occurs during the clinical trial, the Investigator should immediately take appropriate therapeutic measures for the participant and report it to the drug regulatory authority, the health administration, the Sponsor and the Ethics Committee, and sign and date the report.
  - Ensure that data are entered into medical records and case report forms in a truthful, accurate, complete, timely and legal manner.
  - Accept the monitoring and audit by the monitor or auditor dispatched by the Sponsor as well as the audit and inspection by the drug regulatory authority to ensure the quality of the clinical trial.
  - Confirm the expenses related to the clinical trial with the Sponsor and specify them in the contract. During the course of the clinical trial, the Investigator may not charge the participants for the costs required for the investigational product.
  - After the clinical trial is completed, the Investigator must write a summary report, sign and date it, and then send it to the Sponsor.

1. **Biological Sample Testing Unit**

According to the requirements of the clinical trial, the unit will earnestly conduct biological sample testing work of this trial. The bioanalysis laboratory will be responsible for the testing and storage of biological samples to ensure the reliability of the test data, and the data will be analyzed according to the test results.

1. **Biostatistical analysis (data management/statistical unit)**

In accordance with GCP regulations, the unit will earnestly perform responsibilities of data management and statisticians of this trial. After the end of the trial, the unit should be responsible for data management, making statistics on the data according to the formulated statistical plan, and issuing the statistical analysis report.

### Study Site and Trial Participants

#### Sponsor

Name of Sponsor: NovoCodex Biopharmaceuticals Co., Ltd.

Contact Person: LIANG Xuejun

Tel.: (0575) 85211809

Address: 2/F, Auxiliary Building, Environmental Protection Center, No. 58 Changhe Road, Binhai New City, Shaoxing

#### Leading Site

Name of Study Site: Fudan University Shanghai Cancer Center

Principal Investigator: HU Xichun

#### Data Management and Statistics Unit

Nanjing CR Medicon Pharmaceutical Technology Co., Ltd.

#### Contract Research Organization

Nanjing CR Medicon Pharmaceutical Technology Co., Ltd.

### References

1. Chen W, Zheng R. Incidence, mortality and survival analysis of breast cancer in China [J]. Chinese Journal of Clinical Oncology, 2015, 42(13): 668-674.
2. Chen W , Zheng R , Baade P D , et al. Cancer statistics in China, 2015[J]. CA: A Cancer Journal for Clinicians, 2016, 66(2):115-132.
3. Breast Cancer Professional Committee of China Anti-Cancer Association. Expert Consensus on the Clinical Diagnosis and Treatment of Advanced Breast Cancer in China (Version 2018) [J]. Chinese Journal of Oncology, 2018, 40(9): 703-713.
4. Breast Cancer Professional Committee of China Anti-Cancer Association. Guidelines for the Diagnosis and Treatment of Breast Cancer (2018.V1);
5. Moasser, M M . The oncogene HER2: its signaling and transforming functions and its role in human cancer pathogenesis[J]. Oncogene, 2007, 26(45):6469-6487.
6. King C , Kraus M , Aaronson S . Amplification of a novel v-erbB-related gene in a human mammary carcinoma[J]. Science, 1985, 229(4717):974-976.
7. Ross J S , Slodkowska E A , Symmans W F , et al. The HER-2 Receptor and Breast Cancer: Ten Years of Targeted Anti-HER-2 Therapy and Personalized Medicine[J]. The Oncologist, 2009, 14(4):320-368.
8. Compilation group of the Guidelines for the HER2 Detection of Breast Cancer. Guidelines for the HER2 Detection of Breast Cancer (Version 2014) [J]. Chinese Journal of Pathology, 2014, 43(4): 262-267.
9. Jiang Z, Shao Z, Xu B, et al. Expert Consensus on the Clinical Diagnosis and Treatment of Human Epidermal Growth Factor Receptor 2 Positive Breast Cancer 2016 [J]. National Medical Journal of China, 2016, 96(14): 1091-1096.
10. Compilation group of the Guidelines for the HER2 Detection of Breast Cancer (Version 2019). Guidelines for the HER2 Detection of Breast Cancer (Version 2019) [J]. Chinese Journal of Pathology, 2019, 48(3): 169-175.
11. Santa-Maria C A , Nye L , Mutonga M B , et al. Management of Metastatic HER2-Positive Breast Cancer: Where Are We and Where Do We Go From Here?[J]. Oncology, 2016, 30(2).
12. Label of HERCEPTIN^®^ (trastuzumab),2018, Genentech, Inc.
13. Revannasiddaiah S , Seam R , Gupta M . Pertuzumab plus trastuzumab in metastatic breast cancer.[J]. N Engl J Med, 2011, 366(2):109-119.
14. Label of KADCYLA^®^ (ado-trastuzumab emtansine),2019, Genentech, Inc.
15. Label of TYKERB^®^ (lapatinib),2018, Novartis, Inc.
16. Blair H A . Pyrotinib: First Global Approval[J]. Drugs, 2018.
17. Package insert of pyrotinib maleate (Airuini^®^) tablet.
18. Miao P, Tong Z, Hao C, et al. Prognostic analysis and metastatic behavior of breast cancer subtypes [J]. Chinese Journal of Clinical Oncology, 2011, 38(4): 228-231.
19. Krishnamurti U , Silverman J F . HER2 in breast cancer: A review and update[J]. Advances in anatomic pathology, 2014, 21(2):100-107.
20. Nahta R, Esteva F J. Trastuzumab: triumphs and tribulations.[J]. Oncogene, 2007, 26(25):3637-3643.
21. Verma S , Miles D , Gianni L , et al. Trastuzumab Emtansine for HER2-Positive Advanced Breast Cancer[J]. New England Journal of Medicine, 2013, 368(25):1783-91.
22. Dieras V , Harbeck N , Budd G T , et al. Trastuzumab Emtansine in Human Epidermal Growth Factor Receptor 2-Positive Metastatic Breast Cancer: An Integrated Safety Analysis[J]. Journal of Clinical Oncology, 2014, 32(25):2750-2757.
23. Junttila T T , Li G , Parsons K , et al. Trastuzumab-DM1 (T-DM1) retains all the mechanisms of action of trastuzumab and efficiently inhibits growth of lapatinib insensitive breast cancer[J]. Breast Cancer Research & Treatment, 2011, 128(2):347-356.
24. Lopus M , Oroudjev E , Wilson L , et al. Maytansine and Cellular Metabolites of Antibody- Maytansinoid Conjugates Strongly Suppress Microtubule Dynamics by Binding to Microtubules[J]. Molecular Cancer Therapeutics, 2010, 9(10):2689-2699.
25. Wassmer G . Planning and analyzing adaptive group sequential survival trials.[J]. Biometrical Journal, 2010, 48(4):714-729.
26. Cui L , Wang H S J . Modification of Sample Size in Group Sequential Clinical Trials[J]. Biometrics, 1999, 55(3):853-857.

Appendix 1 Clinical Trial Flow Chart

| **Study day/examination or procedure** | **Screening period** | **Treatment period** | | | | **Discontinuation follow-up period** | **Long-term follow-up period ^19^** |
| --- | --- | --- | --- | --- | --- | --- | --- |
|  |  | **Cycle 1 (C1)** | | | **Cycle 2 (C2) and thereafter ^18^** |  |  |
|  | **D-28~D-1** | **D1** | **D7 ± 1 day** | **D14 ± 3 days** | **±3 days** | **28+7 days after discontinuation** | **±7 days** |
| **Signing of informed consent form** | X |  |  |  |  |  |  |
| **Demographic information** | X |  |  |  |  |  |  |
| **Collection of medical history ^1^** | X |  |  |  |  |  |  |
| **Collection of treatment history ^2^** | X |  |  |  |  |  |  |
| **Judgment against inclusion/exclusion criteria** | X |  |  |  |  |  |  |
| **Participant randomization ^3^** | X | |  |  |  |  |  |
| **Tissue sample acquisition for HER2 expression detection ^4^** | X |  |  |  |  |  |  |
| **Ophthalmological examination ^5^** | X |  |  |  |  |  |  |
| **Vital Signs** | X | X**^6^** | X | X | X | X |  |
| **Physical Examination** | X | X |  |  | X | X |  |
| **Blood routine** | X**^7^** |  | X | X | X | X |  |
| **Blood biochemistry** | X**^7^** |  | X | X | X | X |  |
| **Urinalysis** | X**^7^** |  |  |  | X | X |  |
| **12-Lead ECG** | X^7^ | X^8^ | X | X | X | X |  |
| **ECOG Score** | X |  |  |  | X | X |  |
| **Pregnancy test (for women of childbearing potential only)** | X |  |  |  | X | X |  |
| **Coagulation test** | X |  |  |  | X | X |  |
| **Echocardiography** | X |  |  |  | X | X |  |
| **Serum virology ^9^** |  |  |  |  |  |  |  |
| **Hepatitis B five-item test ± HBV DNA** | X |  |  |  | X | X |  |
| **HCV antibody** | X |  |  |  |  |  |  |
| **HIV antibody** | X |  |  |  |  |  |  |
| **Treponema pallidum** | X |  |  |  |  |  |  |
| **Imaging ^10^** |  |  |  |  |  |  |  |
| **Contrast-enhanced CT or MRI of chest, abdomen and pelvic cavity** | X |  |  |  | X |  | X^19^ |
| **Head MRI** | X |  |  |  |  |  |  |
| **Bone emission computed tomography (ECT)** | X |  |  |  |  |  |  |
| **Judgment against RECIST 1.1 Criteria ^11^** | X |  |  |  | X |  | X^19^ |
| **Weight ^12^** |  | X |  |  | X |  |  |
| **Investigational Product Administration** |  | X | | | |  |  |
| **Infusion reaction monitoring ^13^** |  | X |  |  | X |  |  |
| **Immunogenicity sample collection ^14^** |  | X |  |  | X | X |  |
| **PK sample collection ^15^** |  | X | | | |  |  |
| **Telephone visit ^16^** |  |  |  |  |  | X | |
| **Drug dispensing/recovery and patient diary card** ^17^ |  | X | | | | |  |
| **Records of concomitant medications** |  | X | | | | |  |
| **Records of adverse events** | X | | | | | |  |

1. Medical history collection, including but not limited to:
   - Historical data on diagnosis of breast cancer by histopathology or cytology;
   - Molecular typing diagnostic data (may include hormone receptor status, etc.);
   - History of malignancy within 5 years before signing the informed consent form;
   - Presence of previous or current interstitial lung disease requiring hormone therapy, a history of drug-induced interstitial lung disease, a history of radiation pneumonitis, or any evidence indicating clinically active interstitial lung disease;
   - Presence of previous or current cardiac insufficiency, including but not limited to congestive cardiac failure, transmural myocardial infarction, angina requiring drug therapy, clinically significant heart valve disorders, and high-risk arrhythmias;
   - Presence of previous or current serious or clinically significant systemic disease, such as cardiac, pulmonary, metabolic, or hepatic or renal disease.
2. Collection of treatment history, including at least:
   - Prior anti-tumor treatment;
   - Medication within 4 weeks prior to randomization, including at least the use of corticosteroid.
3. The inclusion/exclusion criteria must be reviewed on D-1. Participants who meet the inclusion requirements will receive the participant randomization number, and the randomization time should be as close to the first administration time of the investigational product as possible;
4. The participants should provide tissue samples for central laboratory testing of HER2 expression during the screening period;
5. General examination of the eye, including examination of the ocular appendages and the anterior segment of the eye;
6. The vital signs examination on the day of the first dose of the investigational product will be performed within 30 min before and 2 h ± 30 min after administration, respectively;
7. The blood routine, blood biochemistry, urinalysis and 12-lead ECG during the screening period must be completed within one week before starting dosing; otherwise, they must be retested within one week before dosing;
8. The 12-lead ECG examination on the day of the first dose of the investigational product will be performed once before the administration (test results within 48 h before the start of the administration are acceptable) and 2 h ± 30 min after administration, respectively;
9. Serum virology, which may include HBsAg, HBsAb, HBeAg, HBcAb, HBeAb, HBV DNA quantification (only performed in participants with positive hepatitis B surface antigen), HCV antibody test, HIV antibody test, and treponema pallidum test; during the treatment period, the Hepatitis B five-item test will be performed only in participants who are positive for hepatitis B surface antigen during the screening period, and the Investigator will determine whether the HBV DNA quantitative test is required;
10. Tumor imaging evaluation mainly includes:
    - Contrast-enhanced CT or MRI of chest, abdomen and pelvic cavity: Target lesions and non-target lesions should be selected according to the initial scan, and all subsequent scans should use the same method; for patients who are allergic to contrast agents, CT or MRI plain scan can be performed if determined by the Investigator;
    - Head MRI: The head MRI is a mandatory examination during the screening period, and may be performed in the investigator’s judgment during the treatment period and long-term follow-up visit;
    - Bone emission computed tomography (ECT): ECT is mandatory during the screening period and is performed during the treatment period and long-term follow-up visit at the discretion of the Investigator based on the participant’s specific condition (e.g., presence of bone pain or elevated alkaline phosphatase);
    - The Investigator may decide whether to perform other imaging examinations, such as breast MRI, breast X-ray, breast ultrasound or other imaging examinations of suspected metastatic lesions, based on the signs and status of individual participants.
11. The anti-tumor response evaluation will be performed by the IRC and the Investigator as per RECIST1.1.
12. If the weight/body surface area change from the last measurement or baseline is >± 10% (whichever is greater), the specific dose of ARX788 (as per the weight) or capecitabine (as per the body surface area) should be recalculated, and the weight or body surface area at the new dose should be used as the baseline for subsequent calculations.
13. Before and after ARX788 infusion, close attention should be paid to the occurrence of adverse events in participants and they should be treated in a timely manner. If infusion-related symptoms (such as fever or chills, etc.) occur, the infusion rate can be reduced or the infusion can be discontinued;
14. Blood samples will be collected from all participants in the investigational product group for immunogenicity testing.
15. PK samples will be collected and tested in participants in the investigational product group to assess the population PK profile of ARX788 in patients with HER2-positive advanced breast cancer. If conditions permit, population PK samples should be collected and tested in all participants in the investigational product group. The specific sampling time is as follows:

- Within 0.5 h before the second dose of the investigational product, 1.5 h (± 30 min) after the completion of the dosing (after the completion of the ARX788 intravenous infusion), and 144 h (± 24 h) after the completion of the dosing (after the completion of the ARX788 intravenous infusion);
- Within 0.5 h before the fourth dose of the investigational product, 1.5 h (± 30 min) after the completion of the dosing (after the completion of the ARX788 intravenous infusion), and 144 h (± 24 h) after the completion of the dosing (after the completion of the ARX788 intravenous infusion);

About 5 mL of blood sample will be collected at each blood collection point. For the collection, processing, storage and transportation of samples, see the relevant laboratory standard operating procedures or relevant clinical operation manual for details. The concentration of ARX788, total antibody and the metabolite pAF-AS269 of ARX788 in the blood samples will be tested at each time point.

1. The participant should enter the discontinuation follow-up period after discontinuation. The study doctor should conduct a telephone visit once a week to ask whether the participant has experienced adverse events or used other drugs or treatment methods. The time window for telephone visit is ±3 days. If the participant experiences any adverse event or use any concomitant medication during this period, he/she must report it to the Investigator in a timely manner, and go to the hospital to receive necessary intervention if necessary. During the long-term follow-up period, when it is determined that the participant has disease progression or the participant refuses to come to the hospital for the relevant examinations, the Investigator will conduct a telephone visit every 3 months (based on 30 days/month, with a time window of ±7 days) to learn whether the participant receives other anti-tumor treatments and the participant’s survival information, until the participant dies, withdraws informed consent, is lost to follow-up, refuses telephone follow-up or the trial ends (including completion of the trial and early discontinuation of the trial).
2. During the treatment period, it is necessary to dispense/recover the drugs (only applicable to participants in the control drug group) and the patient diary cards to/from the participants every time they come to the hospital.
3. During the treatment period, the frequency of examinations and procedures in Cycle 2 and thereafter is as follows:

- Weight measurement, vital signs, physical examination, blood routine, blood biochemistry, urinalysis and 12-lead ECG should be performed within 3 days before the administration of the investigational product in C2 and each subsequent cycle; in particular, participants in the investigational product group will also undergo one vital signs and 12-lead ECG test 2 h ± 30 min after each administration of the investigational product;
- Echocardiogram, coagulation, ECOG score, serum virology (hepatitis B five-item test ± HBV DNA), pregnancy test and immunogenicity sample collection (only applicable to participants in the investigational product group) should be performed within 3 days before the administration of the investigational product in C3 and every 2 cycles thereafter;
- Tumor imaging and efficacy evaluation will be performed every 6 weeks ± 3 days after randomization.

1. After the discontinuation follow-up period, the participants will enter the long-term follow-up period to provide sufficient data to support the analysis and assessment of long-term benefit endpoints. During the long-term follow-up period, unless the participant discontinues the drug due to IRC-assessed disease progression or death, the participant must come to the hospital for a tumor status check at the original scheduled time (every 6 weeks ± 3 days after randomization) until IRC-assessed disease progression or death or refusal to come to the hospital for follow-up or the end of the trial (including completion of the trial and early discontinuation of the trial).

Appendix 2 ECOG PS Score

| **Activity Status** | **Description** |
| --- | --- |
| **0** | Asymptomatic, fully active, and able to carry on all predisease performance without restrictions. |
| **1** | Symptomatic, fully ambulatory but restricted in physically strenuous activity and able to carry out performance of a light or sedentary nature, eg, light housework, office work. |
| **2** | Symptomatic, ambulatory and capable of all self-care but unable to carry out any work activities. Up and about more than 50% of waking bours: in bed less than 50% of day. |
| **3** | Symptomatic, capable of only limited self-care, confined to bed or chair more than 50% of waking hours, but not bedridden. |
| **4** | Completely disabled. Cannot carry on any self-care. Totally bedridden. |
| **5** | Death |

Appendix 3 Response Evaluation Criteria in Solid Tumors RECIST 1.1 Quick Reference

New Response Evaluation Criteria in Solid Tumors: Revised RECIST Guidelines (Version 1.1). Eur J Cancer 2009; 45: 228-47.

**Participant eligibility**

Only patients with measurable disease at baseline should be included in protocols where objective tumor response is the primary endpoint. Measurable disease is defined as the presence of at least one measurable lesion.

**Assessment methods**

The same method of assessment and the same technique should be used to characterize each identified and reported lesion at baseline and during follow-up.

- - CT is the best currently available and reproducible method to measure lesions selected for response assessment. MRI is also acceptable in certain situations (e.g., for body scans, not lung scans).
  - Lesions on chest X-ray are acceptable as measurable lesions when they are clearly defined and surrounded by aerated lung. However, CT is preferable.
  - Clinical lesions will only be considered measurable when they are superficial and ≥ 10 mm diameter as assessed using calipers. For the case of skin lesions, documentation by color photography including a ruler to estimate the size of the lesion is suggested.
  - Ultrasound should not be used to measure tumor lesions.
  - Tumor markers alone cannot be used to assess response. If markers are initially above the upper normal limit, they must normalize for a patient to be considered in complete clinical response when all lesions have disappeared.
  - Cytological and histological studies can be performed on rare cases (e.g., to evaluate residual masses to differentiate between partial response and complete response, or to evaluate new or enlarged effusion to identify disease progression and disease response/stabilization).
  - Endoscopy and laparoscopy are not recommended. However, they can be used to confirm complete pathological response.

**Baseline disease assessment**

All baseline evaluations should be performed as close as possible to the treatment start and never more than 4 weeks or longer before the beginning of the treatment.

**Measurable lesions**

Must be accurately measured in at least one dimension (longest diameter in the plane of measurement is to be recorded) with a minimum size of:

- - 10 mm by CT scan (CT scan slice thickness no greater than 5 mm; when CT scans have slice thickness greater than 5 mm, the minimum size for a measurable lesion should be twice the slice thickness).
  - 10 mm caliper measurement by clinical examination (lesions which cannot be accurately measured with calipers should be recorded as non-measurable).
  - 20 mm by chest X-ray.

Malignant lymph nodes: To be considered pathologically enlarged and measurable, a lymph node must be ≥ 15 mm in short axis when assessed by CT scan (CT scan slice thickness recommended to be no greater than 5 mm). At baseline and in follow-up, only the short axis will be measured and followed.

Lytic bone lesions or mixed lytic-hyperplastic lesions, with identifiable soft tissue components, that can be evaluated by cross-sectional imaging techniques such as CT or MRI can be considered as measurable lesions if the soft tissue component meets the definition of measurability described above.

“Cystic lesions” thought to represent cystic metastases can be considered as measurable lesions, if they meet the definition of measurability described above. However, if non-cystic lesions are present in the same patient, these are preferred for selection as target lesions.

**Non-measurable lesions**

Non-measurable lesions are all other lesions, including small lesions (longest diameter < 10 mm or pathological lymph nodes with ≥ 10 to < 15 mm short axis) as well as truly non-measurable lesions. Lesions considered truly non-measurable include: leptomeningeal disease, ascites, pleural or pericardial effusion, inflammatory breast disease, lymphangitic involvement of skin or lung, abdominal masses, and abdominal organomegaly identified by physical examination that is not measurable by reproducible imaging techniques. Hyperplastic bone lesions are non-measurable.

Lesions that have been are previously treated locally (e.g., lesions situated in a previously irradiated area, or in an area participanted to other loco-regional therapy), are usually not considered measurable unless there has been demonstrated progression in the lesion. Study protocols should detail the conditions under which such lesions would be considered measurable.

**Target lesions**

- - All measurable lesions up to a maximum of two lesions per organ and five lesions in total, representative of all involved organs should be identified as target lesions and recorded and measured at baseline.
  - Target lesions should be selected on the basis of their size (lesions with the longest diameter), be representative of all involved organs, but in addition should be those that lend themselves to reproducible repeated measurements.
  - Calipers are used for clinical evaluations, and all measurements should be recorded in meters.

A sum of the diameters (longest for non-nodal lesions, short axis for nodal lesions) for all target lesions will be calculated and reported as the baseline sum diameters. The baseline sum diameters will be used as reference to further characterize any objective tumor regression in the measurable dimension of the disease. If lymph nodes are to be included in the sum, only the short axis is added into the sum.

**Non-target lesions**

All other lesions (or sites of disease) that are not identified as target lesions, including pathological lymph nodes and all non-measurable lesions, should be identified as non-target lesions and should also be recorded at baseline. Measurements are not required and these lesions should be followed as “present”, “absent”, or in rare cases “unequivocal progression”.

**Response criteria**

**Evaluation of target lesions**

Complete response (CR):

Disappearance of all target lesions. Any pathological lymph nodes (whether target or non-target) must have reduction in short axis to < 10 mm.

Partial response (PR):

At least a 30% decrease in the sum of diameters of target lesions, taking as reference the baseline sum diameters.

Progressive disease (PD):

At least a 20% increase in the sum of diameters of target lesions, taking as reference the smallest sum on study (this includes the baseline sum). In addition, the sum must also demonstrate an absolute increase of at least 5 mm (appearance of one or more new lesions is also considered progression).

Stable disease (SD):

Neither sufficient shrinkage to qualify for PR nor sufficient increase to qualify for PD.

**Special notes on the assessment of target lesions:**

Lymph nodes identified as target lesions should always have the actual short axis measurement recorded, even if the nodes regress to below 10 mm on study. When lymph nodes are included as target lesions, the sum of lesions may not be zero even if complete response criteria are met, since a normal lymph node is defined as having a short axis of <10 mm.

Target lesions that become “too small to measure”: While on study, all lesions (nodal and non-nodal) recorded at baseline should have their actual measurements recorded at each subsequent evaluation, even when very small. However, sometimes lesions or lymph nodes become so faint on CT scan that the radiologist may not feel comfortable assigning an exact measure and may report them as being “too small to measure”. In this case, a default value of 5 mm should be assigned.

Lesions that split or coalesce on treatment: When non-nodal lesions are “fragment”, the longest diameters of the fragmented portions should be added together to calculate the target lesion sum. Similarly, as lesions coalesce, a plane between them may be maintained that would aid in obtaining maximal diameter measurements of each individual lesion. If the lesions have truly coalesced such that they are no longer separable, the vector of the longest diameter in this instance should be the maximal longest diameter for the coalesced lesion.

**Evaluation of non-target lesions**

Complete response (CR):

Disappearance of all non-target lesions and normalization of tumor marker level. All lymph nodes must be non-pathological in size (< 10 mm short axis).

Non-CR/Non-PD:

Persistence of one or more non-target lesion(s) and/or maintenance of tumor marker level above the normal limits.

Progressive disease (PD):

Unequivocal progression of existing non-target lesions.

When the patient has measurable disease, to achieve “unequivocal progression” on the basis of the non-target disease, there must be an overall level of substantial worsening in non-target disease such that, even in presence of SD or PR in target disease, the overall tumor burden has increased sufficiently to merit discontinuation of therapy. A modest increase in the size of one or more non-target lesions is usually not sufficient to quality for “unequivocal progression” status.

When the patient has only non-measurable disease, there is no measurable disease assessment to factor into the interpretation of an increase in non-measurable disease burden. Because worsening in non-target disease cannot be easily quantified, a useful test that can be applied when assessing patients for unequivocal progression is to consider if the increase in overall disease burden based on the change in non-measurable disease is comparable in magnitude to the increase that would be required to declare PD for measurable disease. Examples include an increase in a pleural effusion from “trace” to “large”, or an increase in lymphangitic disease from localized to widespread.

**New lesions**

The appearance of new malignant lesions denotes disease progression

- - - The finding of a new lesion should be unequivocal. (For example, not attributable to differences in scanning technique, change in imaging modality or findings thought to represent something other than tumor. This is particularly important when the patient’s baseline lesions show partial or complete response)
    - If a new lesion is equivocal, for example because of its small size, continued therapy and follow-up evaluation will clarify if it represents truly new disease. If repeat scans confirm there is definitely a new lesion, then progression should be declared using the date of the initial scan.
    - A lesion identified on a follow-up study in an anatomical location that was not scanned at baseline is considered a new lesion and will indicate disease progression.

It is sometimes reasonable to incorporate the use of FDG-PET scanning to complement CT scanning in assessment of progression (particularly possible new disease). New lesions on the basis of FDG-PET imaging can be identified according to the following algorithm:

Negative FDG-PET at baseline, with a positive FDG-PET at follow-up is a sign of PD based on a new lesion. No FDG-PET at baseline and a positive FDG-PET at follow-up:

- If the positive FDG-PET at follow-up corresponds to anew site of disease confirmed by CT, this is PD.
- If the positive FDG-PET at follow-up is not confirmed as a new site of disease on CT, additional follow-up CT scans are needed to determine if there is truly progression occurring at that site (if so, the date of PD will be the date of the initial abnormal FDG-PET scan).
- If the positive FDG-PET at follow-up corresponds to a pre-existing site of disease on CT that is not progressing on the basis of the anatomic images, this is not PD.

**Time point response**

The following table provides a summary of the overall response status calculation at each time point for patients who have measurable disease at baseline: patients with target (+/– non-target) lesions.

| **Target lesions** | **Non-target lesions** | **New lesions** | **Overall response** |
| --- | --- | --- | --- |
| CR | CR | No | CR |
| CR | Non-CR/non-PD | No | PR |
| CR | NE | No | PR |
| PR | Non-PD or not all evaluated | No | PR |
| SD | Non-PD or not all evaluated | No | SD |
| Not all evaluated | Non-PD | No | NE |
| PD | Any | Yes or No | PD |
| Any | PD | Yes or No | PD |
| Any | Any | Yes | PD |

Note: CR = complete response; PR = partial response; SD = stable disease; PD = progressive disease; NE = not evaluable

**Confirmation**

In non-randomized trials where response is the primary endpoint, confirmation of PR and CR is required to ensure responses identified are not the result of measurement error. This will also permit appropriate interpretation of results in the context of historical data where response has traditionally required confirmation in such trials. However, in all other circumstances (i.e., in randomized trials (phase II or III) or studies where stable disease or progression are the primary endpoints), confirmation of response is not required since it will not add value to the interpretation of trial results. However, elimination of the requirement for response confirmation may increase the importance of central review to protect against bias, in particular in studies which are not blinded.

In the case of SD, measurements must have met the SD criteria at least once after study entry at a minimum interval (in general not less than 6–8 weeks) that is defined in the study protocol.

**Missing assessments and non-evaluable designation**

When no imaging/measurement is done at all at a particular time point, the patient is not evaluable (NE) at that time point. If only a part of lesions is measured at an assessment, usually the case is also considered NE at that time point, unless a convincing argument can be made that the contribution of the individual missing lesion(s) would not change the assigned time point response. This would be most likely to happen in the case of PD.

**Reporting of results**

- All patients included in the study must be assessed for response to treatment, even if there are major protocol treatment deviations or if they are ineligible. Each patient will be assigned one of the following categories: 1) complete response, 2) partial response, 3) stable disease, 4) progressive disease, 5) early death from malignant disease, 6) early death from toxicity, 7) early death because of other cause, or 9) unknown (not assessable, insufficient data).
- All of the patients who met the eligibility criteria should be included in the main analysis of the response rate. Patients in response categories 4-9 should be considered as failing to respond to treatment (disease progression). Thus, an incorrect treatment schedule or drug administration does not result in exclusion from the analysis of the response rate. Precise definitions for categories 4-9 will be protocol specific.
- All conclusions should be based on all eligible patients.
- Sub-analyses may then be performed on the basis of a subset of patients, excluding those for whom major protocol deviations have been identified (e.g., early death due to other reasons, early discontinuation of treatment, major protocol violations, etc.). However, these sub-analyses may not serve as the basis for drawing conclusions concerning treatment efficacy, and the reasons for excluding patients from the analysis should be clearly reported.
- The 95% confidence intervals should be provided.

Appendix 4 Management of Infusion-Related Reactions

Symptoms of infusion-related reactions include fever, chills, stiffness, sweating and headache. In order to prevent participants from experiencing influenza-like infusion reactions, routine prophylactic infusion medications (such as phenergan and/or indomethacin) can be administered 30 minutes before the start of the infusion of the investigational product upon the investigator’s judgment. Infusion reaction symptoms caused by the investigational product can be treated by referring to the following options:

- Grade 1 - Mild:
  - Mild temporary reaction;
  - Infusion interruption is not required;
  - No intervention is required.

At this time, the infusion rate of the investigational product should be reduced to about 50% of the current infusion rate, and any deterioration should be closely monitored. The total infusion time of the investigational product should not exceed 180 min as much as possible. If the infusion is not completed in 180 min and the drug preparation has been completed for less than 6 hours, the infusion of the drug can continue. Otherwise, the remaining drug solution should be discarded and the actual dose should be recorded.

- Grade 2 - Moderate:
  - Interruption of treatment or infusion is required, which works immediately after symptomatic treatment (e.g., antihistamines, non-steroidal anti-inflammatory drugs, anesthetics, IV fluids);
  - Cases where prophylactic medication is required for ≤ 24 hours.

At this time, the infusion of investigational product should be stopped. Once the study infusion reaction has resolved or has decreased in severity to grade 1 or less, the infusion can be restarted at approximately 50% of the previous rate, and any deterioration should be monitored closely.

- Grade 3 or 4: Moderate or life-threatening
  - Grade 3:

1. Delayed onset of action after treatment (e.g., no rapid onset of action after symptomatic medication and/or temporary interruption of infusion);
2. Recurrence of symptoms after initial improvement;
3. Clinical sequelae requiring hospitalization for treatment.
   - Grade 4: It has life-threatening consequences and requires emergency intervention;

At this time, the participant must immediately stop the infusion of the investigational product and permanently stop the investigational product treatment.

If the infusion rate of the investigational product is reduced to 50% of the previous rate due to infusion-related reactions, the infusion rate must also remain at 50% of the previous infusion rate for the rest of the treatment.

Appendix 5 New York Heart Association Functional Classification of Heart Failure

| **Classification** | **Symptoms** |
| --- | --- |
| Class 1 | No limitation of physical activity. Ordinary physical activity does not cause undue fatigue, palpitation, dyspnea. |
| Class 2 | Slight limitation of physical activity. Comfortable at rest. Ordinary physical activity results in fatigue, palpitation, dyspnea. |
| Class 3 | Marked limitation of physical activity. Comfortable at rest. Less than ordinary activity causes fatigue, palpitation, or dyspnea. |
| Class 4 | Unable to carry on any physical activity without discomfort. Symptoms of heart failure at rest. If any physical activity is undertaken, discomfort increases. |
| Note: New York Heart Association Standards Committee, Nomenclature and criteria for diagnosis of diseases of the heart and great vessels. 9th ed. Boston, MA: Little, Brown & Co, 1994: 25. | |
